# Discovery of an exquisitely selective WDR5 chemical probe accelerated by a high-quality DEL–ML Hit†

**DOI:** 10.1039/d5cb00109a

**Published:** 2025-07-17

**Authors:** Lasse Hoffmann, Christopher Lenz, Frederic Farges, Serah W. Kimani, Johannes Dopfer, Sabrina Keller, Martin Peter Schwalm, Hanna Holzmann, Andreas Kraemer, Aiping Dong, Fengling Li, Irene Chau, Levon Halabelian, Matthias Gstaiger, Susanne Müller, Stefan Knapp, Václav Němec

**Affiliations:** a Institute for Pharmaceutical Chemistry, Johann Wolfgang Goethe-University Max-von-Laue-Str. 9 D-60438 Frankfurt am Main Germany nemec@pharmchem.uni-frankfurt.de knapp@pharmchem.uni-franfurt.de; b Structural Genomics Consortium, Buchmann Institute for Molecular Life Sciences, Johann Wolfgang Goethe-University Max-von-Laue-Str. 15 D-60438 Frankfurt am Main Germany; c Structural Genomics Consortium, University of Toronto Toronto ON Canada; d Inst. f. Molekulare Systembiologie, Otto-Stern-Weg 3 8093 Zürich Switzerland; e German Cancer Consortium (DKTK), German Cancer Research Center (DKFZ) DKTK site Frankfurt-Mainz 69120 Heidelberg Germany; f Department of Pharmacology and Toxicology, University of Toronto Toronto ON Canada; g Princess Margaret Cancer Centre, University Health Network Toronto ON Canada

## Abstract

Herein we present the rapid development of LH168, a potent and highly selective chemical probe for WDR5, streamlined by utilizing a DEL–ML (DNA encoded library–machine learning) hit as the chemical starting point. LH168 was comprehensively characterized in bioassays and demonstrated potent *in cellulo* target engagement at the WIN-site pocket of WDR5, with an EC_50_ of approximately 10 nM, a long residence time, and exceptional proteome-wide selectivity for WDR5. In addition, we present the X-ray co-crystal structure and provide insights into the structure–activity relationships (SAR). In parallel, we developed a matched negative control compound as well as an alkyne analog (compound 16) to facilitate the development of bifunctional molecules. Taken together, we provide the scientific community with a well-characterized chemical probe to enable studies and functional manipulation of WDR5 in a cellular context, as this protein represents a therapeutically relevant target with scaffolding functions that influence multiple cellular processes.

## Abstract 2

LH168, a potent and highly selective chemical probe for WDR5, developed based on DEL–ML (DNA encoded library–machine learning) hit.

## Graphical abstract

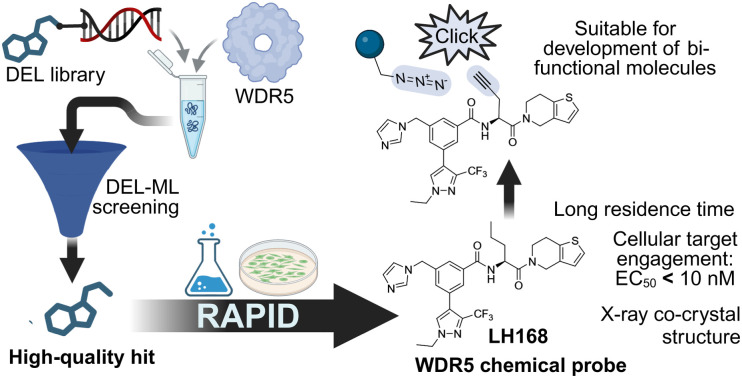

## Introduction

WDR5 is a highly conserved WD40-repeat protein that plays a central role in numerous biological processes through its function as a molecular scaffold. Structurally, WDR5 is composed of a WD40 domain with an N-terminal extension and features a distinctive 7-bladed β-propeller architecture.^1^ Its primary function is to mediate the assembly of large protein complexes, such as the non-specific lethal (NSL) acetyltransferase complex, the nucleosome remodeling and deacetylase (NuRD) complex, and the mixed-lineage leukemia protein (SET1/MLL) methyltransferase complexes.^2^ Through these interactions, WDR5 regulates chromatin modification and gene expression, including the recruitment of c-MYC to chromatin, a key process implicated in the pathogenesis of c-MYC-dependent cancers such as acute myeloid leukaemia.^2^ Overexpression of WDR5 has been correlated with poor prognosis in a range of cancers, including neuroblastoma, breast, bladder, and colorectal cancers, further underscoring its potential as a therapeutic target.^3,4^

Two distinct binding sites have been identified on the WDR5 WD40 domain: (I) the WDR5-interacting (WIN) site, which engages with SET-family methyltransferases and has been successfully targeted by small-molecule inhibitors such as OICR-9429^5^ and MM-598^6^ or degraders^4,7^ and (II) the WDR5-binding motif (WBM) site, which is responsible for c-MYC recruitment and features a shallower binding surface that has also been targeted by small-molecule inhibitors.^2,8^ The WIN site contains a characteristic arginine-binding cavity which mediates the interaction with proteins containing an arginine-containing WIN motif.^1^

The chemical space of drug-like compounds is estimated to include around 10^60^ possible molecules,^9,10^ offering immense opportunities for medicinal chemistry. However, efficient mapping of this vast chemical space to identify suitable hits remains one of the biggest challenges in drug discovery. Traditional high-throughput screening methods, while effective, often struggle to navigate this vast diversity efficiently. In response to this challenge, DNA-encoded library (DEL) technology has emerged over the past decade as a revolutionary approach in lead discovery.^11^ DEL technology enables the simultaneous interrogation of millions to billions of compounds in a single, multiplexed experiment. This method not only dramatically reduces the cost of high-throughput screening (HTS) but it also facilitates the exploration of unprecedented chemical diversity in minute reaction volumes.^11^

By integrating DEL with machine learning, the sensitivity and throughput of hit identification can be significantly enlarged by mining also commercially available compounds that may be difficult to synthesize in the presence of DNA. This approach significantly expands the exploration of chemical space, potentially increasing the probability for identification of high-quality hits and accelerating the progression from screening to potent biologically active compounds.^12^

In this article we demonstrate the rapid development of a potent and exquisitely selective WDR5 chemical probe, which was streamlined by use of a high-quality DEL–ML hit which we have identified and disclosed recently in a WDR domain focused DEL–ML effort.^13^ This includes comprehensive profiling in a variety of *in vitro* and cellular assays, selectivity assessment using chemoproteomics and binding mode elucidation by crystallographic methods.

## Results and discussion

MR43378 was identified using DEL–ML screening as a small molecule WDR5 ligand.^13^ Given its drug-like structure, synthetic tractability and double digit nanomolar potency, MR43378 represented an excellent starting point for further optimization towards a selective chemical probe or lead for drug discovery.

**Fig. 1:**
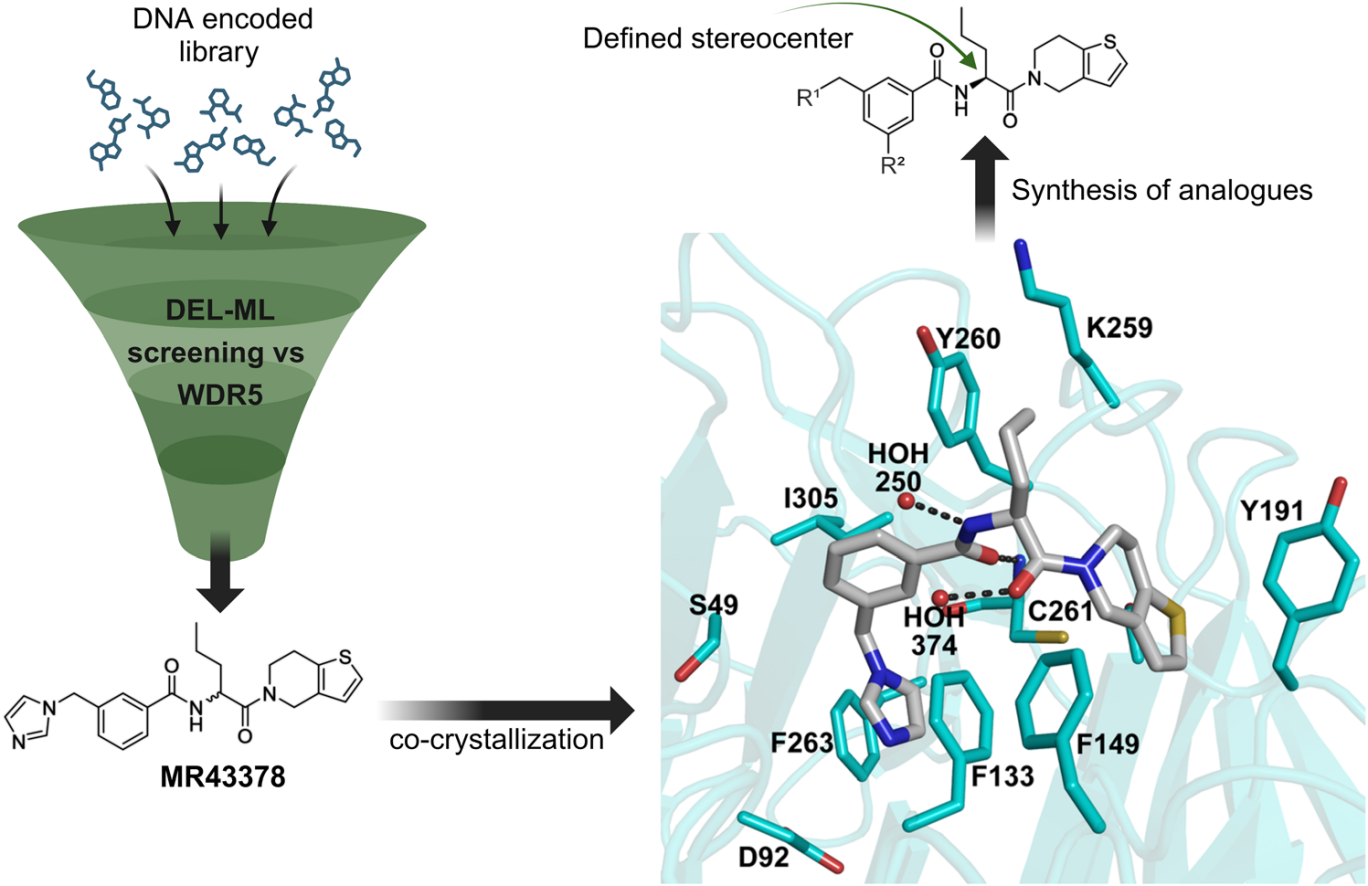
Based on a DEL selection, 66 compounds were predicted by machine learning (ML), of which 11 demonstrated binding affinities in the 0.069–87 μM *K*_D_ range, as assessed by SPR. MR43378 emerged as a promising starting point for further optimization. Protein crystallography revealed its binding mode, indicating that the *S*-enantiomer ((*S*)-MR43378) was bound in the WIN-site pocket.

Medicinal chemistry optimization of MR43378 was further facilitated by the obtained co-crystal X-ray structure with WDR5 (PDB ID: 8T5I), revealing its binding to the WIN-site pocket. Mining publicly available structural information and binding interactions of other known WIN-site WDR5 ligands (Fig. S1, ESI†)^5,14,15^ led us to hypothesize that further decoration of the central phenyl ring (Fig. 1, substituent R^2^) and structural modification of the imidazole moiety (Fig. 1, substituent R^1^) might be an efficient approach for improving biological activity, aiming to develop a high-quality chemical probe.

**Scheme 1:**
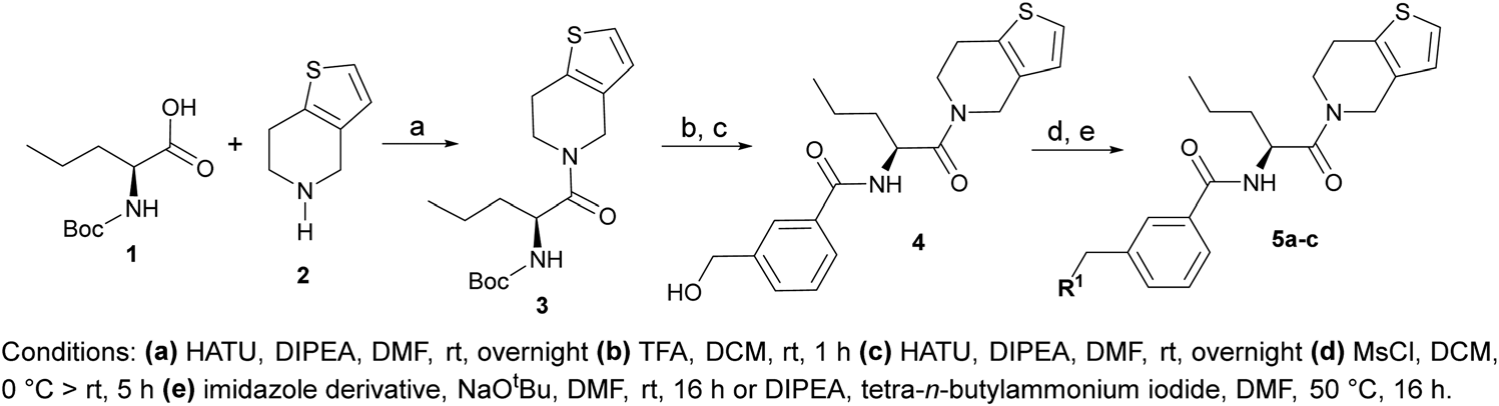
Synthetic route allowing manipulation of position R^1^.

Prior to incorporating the additional moiety on the phenyl ring, we decided to develop a relatively straightforward synthetic route (Scheme 1) to explore variations of the imidazole moiety (SAR Table 1), which mimics a conserved arginine residue present in SET1 protein family members, natural binding partners of WDR5, forming important binding interactions.^16^ Inspired by published SAR data for other WDR5 ligands,^14^ we assumed that introduction of additional amine (or imine as there is a tautomeric equilibrium) might contribute to stronger affinity. In addition, we also re-synthesized the DEL–ML hit (*S*)-MR43378 (5a) as a pure *S*-enantiomer.

**Table 1:** SAR for the position R^1^. Activities of compounds 5a–c against WDR5. SPR *K*_D_ was obtained using kinetic fit

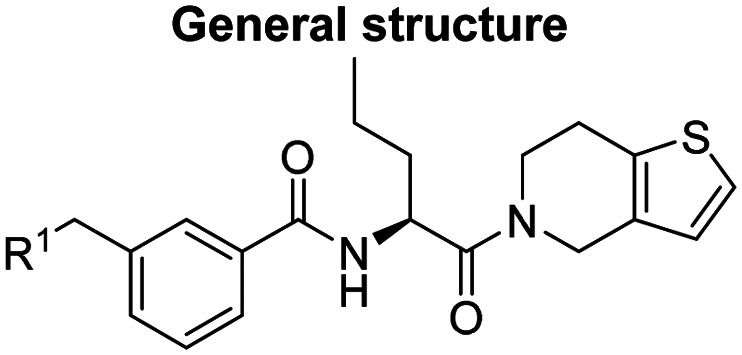						
Compound	R^1^	EC_50_ NanoBRET [nM]		*K*_D_ SPR [nM]	Residence time [s]	DSF Δ*T*_m_ [°C]
		Intact	Permeabilized			
(*S*)-MR43378 (5a)	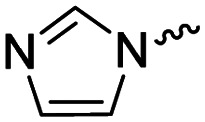	>10 000	>10 000	77.3	15	17.7
5b	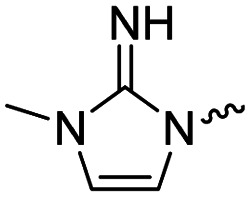	>10 000	7276	49.5	26	22.1
5c	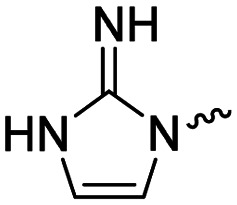	ND	>10 000	ND	ND	15.0

**Scheme 2:**
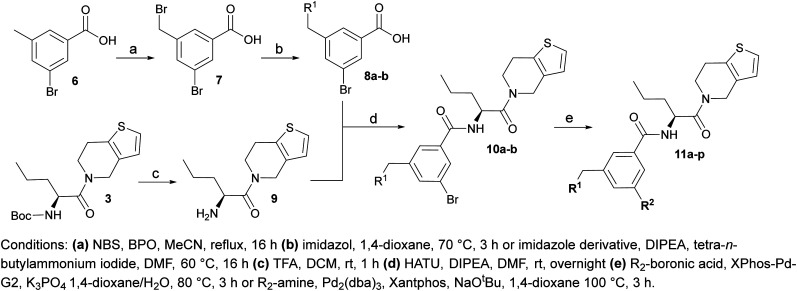
Synthetic route allowing manipulation of positions R^1^ and R^2^.

To establish SAR for our chemical series, selected compounds were profiled using surface plasmon resonance (SPR), differential scanning fluorimetry (DSF) and NanoBRET target engagement to monitor the interaction with WDR5 in cellular environment. SPR and DSF assays indeed confirmed that introduction of 1-methyl-1*H*-imidazol-2-amine moiety led to more potent compound (5b) *in vitro*. Therefore, we have established a modified synthetic sequence allowing for modification of the R^2^ substituent in the last synthetic step and convenient preparation of 12 analogues 11a-l (Scheme 2 and Table 2).

Structure–activity relationships (SAR) for R^1^ and R^2^ substitutions. Activities of compounds 11a–p against WDR5 are shown. SPR *K*_D_ values were determined by kinetic fitting

**Table:** 

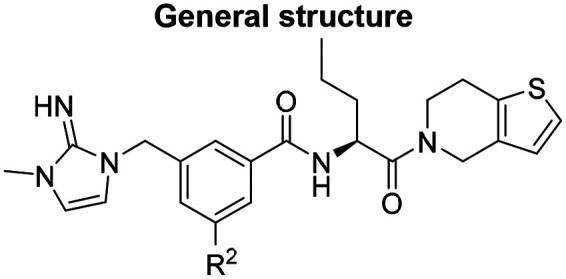					
Compound	R^2^	EC_50_ NanoBRET [nM]		*K*_D_ SPR [nM]	Residence time [s]
		Intact	Permeabilized		
5b	H	>10 000	7276	49.5	26
11a	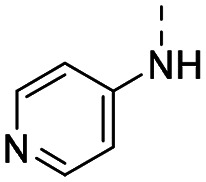	>10 000	870	ND	ND
11b	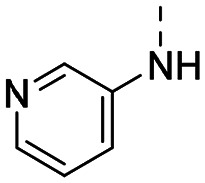	>10 000	1038	ND	ND
11c	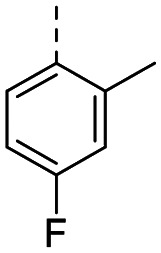	725	93	1.3	1233^a^
11d	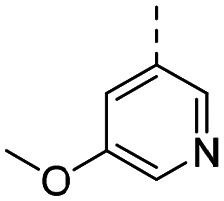	>10 000	424	ND	ND
11e	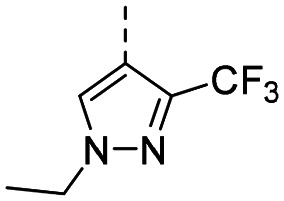	667	28	<1	2580^a^
11f	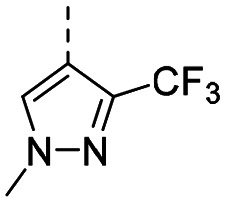	1106	23	<1	1830^a^
11g	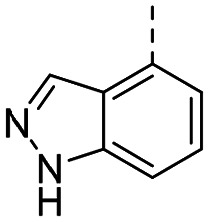	>10 000	109	ND	ND
11h	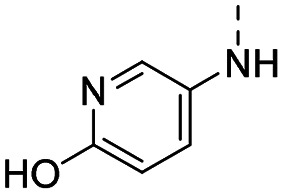	>10 000	3562	ND	ND
11i	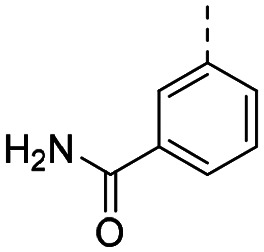	>10 000	91	<1	282
11j	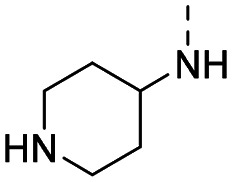	>10 000	>10 000	ND	ND
11k	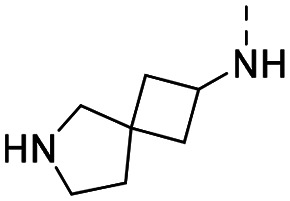	>10 000	8293	ND	ND
11l	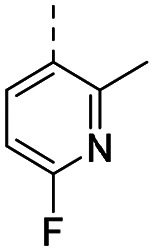	2265	41	<1	1060^a^

a Values marked with an asterisk were obtained from dissociation curves that did not reach baseline due to a slow off-rate, which may affect the accuracy of residence time calculations.

**Table:** 

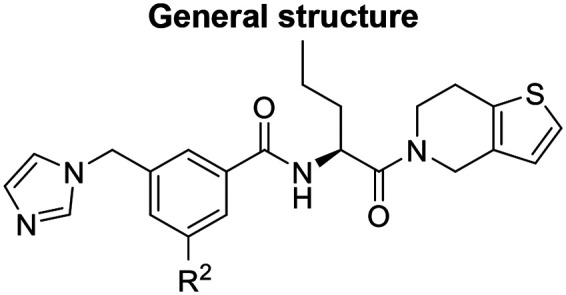					
Compound	R^2^	EC_50_ NanoBRET [nM]		K_D_ SPR [nM]	Residence time [s]
		Intact	Permeabilized		
(*S*)-MR43378 (5a)	H	>10 000	>10 000	77.3	15
11m	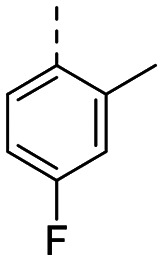	255	674	5.4	396^a^
11n	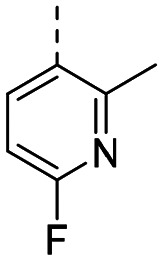	169	115	1.8	456^a^
LH168 (11o)	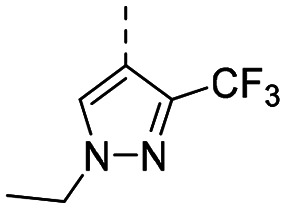	10	154	1.9	714
11p	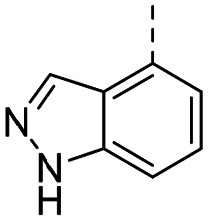	ND	2617	ND	ND
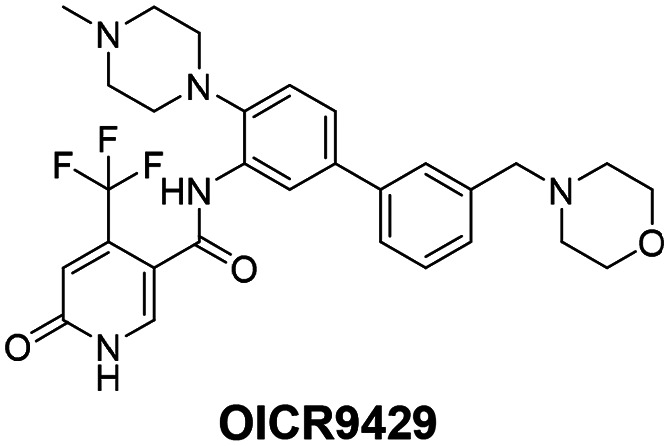		596	1486	8.6	44

However, the aminoimidazole series suffered from poor cellular activity, likely due to poor cell membrane penetration, as determined by NanoBRET experiments comparing target engagement activity in intact *versus* digitonin-permeabilized cells. Therefore, we returned to the imidazole moiety and synthesized four additional analogues (compounds 11m–p), which demonstrated improved potency in live cells despite lower *in vitro* potency.

Notably, NanoBRET EC_50_ values measured in permeabilized cells were, in some cases (including LH168), significantly higher than in intact cells. This phenomenon has been observed consistently across several biological replicates and different chemical series targeting WDR5, including published ligands.^7,17^ We hypothesize that this may result from complex kinetic effects related to the formation of multi-component complexes in the cellular environment. Upon rupture of the cell membrane by digitonin, kinetics governing the formation and dissociation of these complexes might be disturbed by changes in concentrations, possibly affecting interactions with the WDR5 WIN site.

**Fig. 2:**
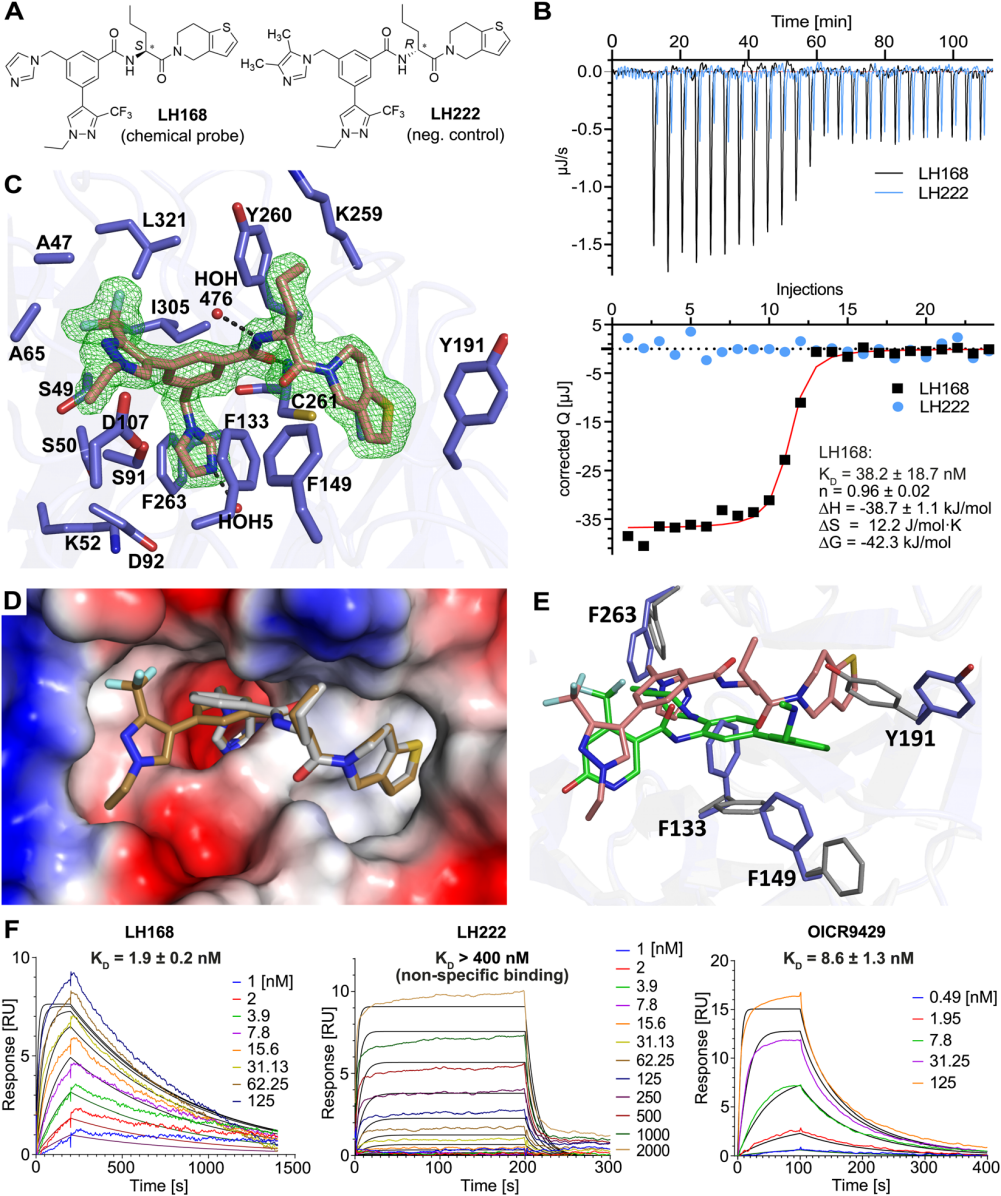
(A) Chemical structures of chemical probe LH168 and negative control compound LH222 (B) ITC of LH168 and LH222 binding to WDR5. Thermodynamic parameters: enthalpy change (Δ*H*), entropy change (Δ*S*), dissociation constant (*K*_D_), free Gibbs energy change (Δ*G*) and binding stoichiometry (*n*). Confidence intervals were calculated at a significance level (*α*) of 0.05. (C) Co-crystal structure of LH168 (beige sticks) and WDR5 (blue sticks show selected residues of the WIN site), electron density of LH168 in green, PDB ID: 9D5Z. (D) Comparison of binding modes of (*S*)-MR43378 (violet sticks, PDB ID: 8T5I) and LH168 (blue sticks, PDB ID: 9D5Z) in WDR5 (surface representation). (E) Comparison of binding modes of OICR9429 (ligand in green sticks, AA residues in grey sticks) and LH168 (ligand in beige sticks, AA residues in blue sticks, PDB ID: 9D5Z) bound to WDR5 in two distinct conformations as highlighted by the different orientation of selected AA residues. (F) Exemplary SPR sensorgram overlay plots of chemical probe LH168, negative control LH222 and known ligand OICR9429. *K*_D_ values were determined using a kinetic 1 : 1 Langmuir interaction fit and are depicted as mean ± SD (*n* = 3).

Based on its favorable *in cellulo* potency, LH168 was selected for further characterization as our chemical probe candidate. Subsequently, we aimed to develop a structurally related negative control compound by introducing a slight modification to the LH168 structure. Inversion of the configuration at the chiral center, or introduction of the dimethyl imidazole moiety alone, did not sufficiently reduce WDR5 binding affinity (Fig. S2, S5 and S6, ESI†). However, the combination of both modifications resulted in LH222 (Fig. 2), showing sufficiently low binding activity.

**Fig. 3:**
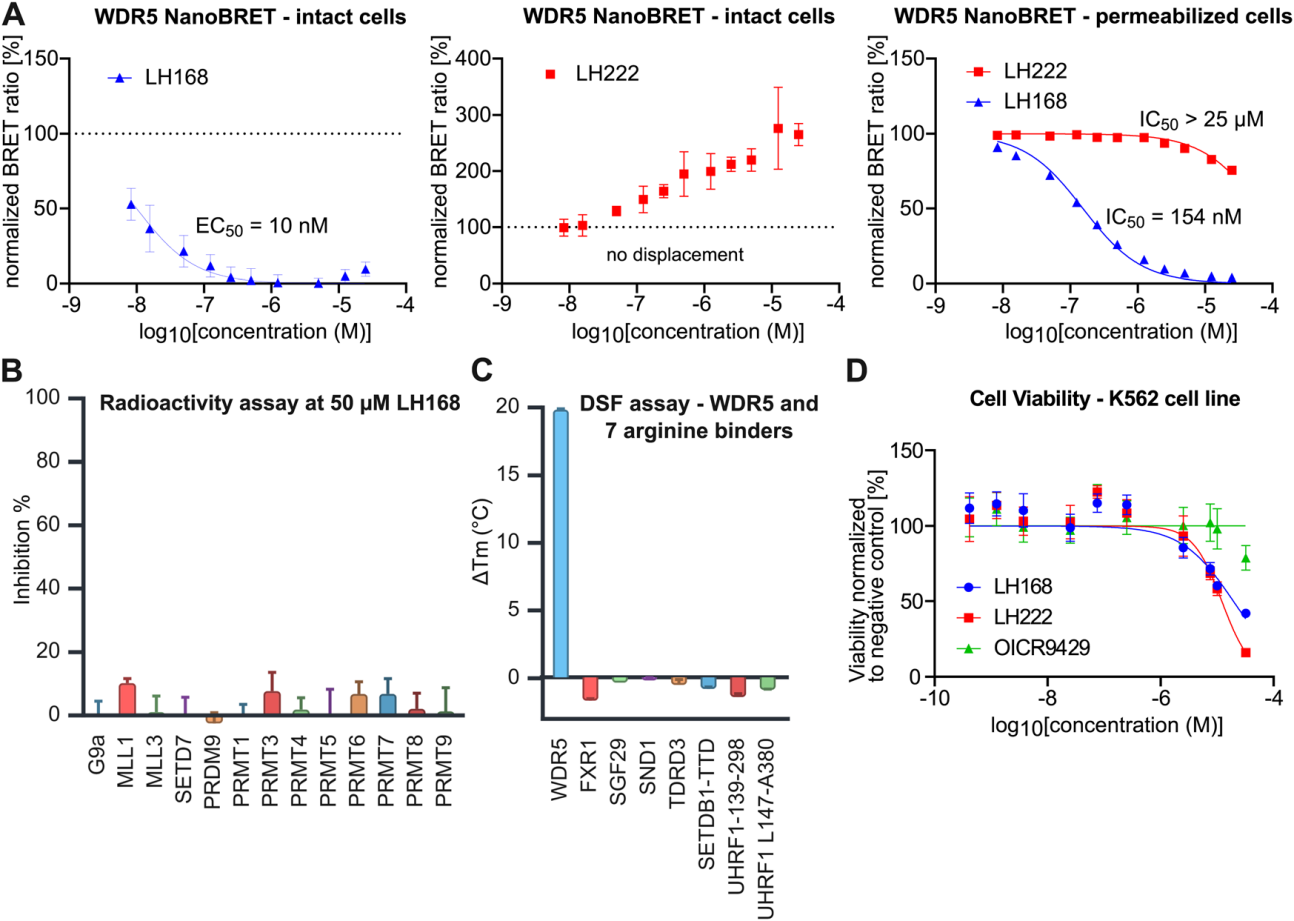
Profiling of LH168 and negative control LH222. (A) NanoBRET target engagement profiling in HEK293 cells, measured in intact cells and in digitonin-permeabilized cells. (B) Radioactivity assay at 50 μM conc. of LH168. (C) DSF assay against seven arginine binders and WDR5. (D) CellTiterGlo – cell viability assay. LH168, LH222 and OICR8429 were incubated with K562 cells for 5 days.

**Fig. 4:**
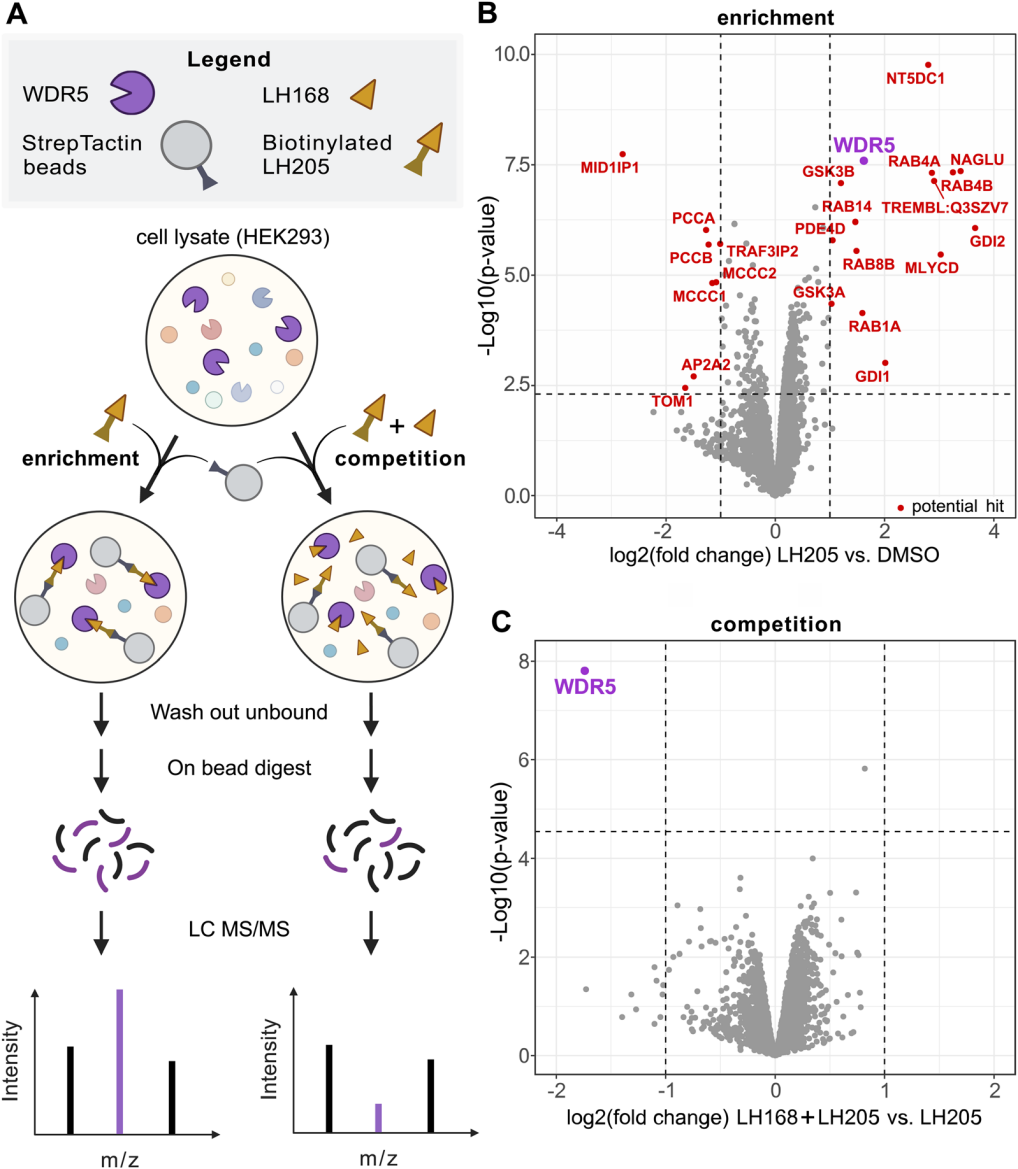
(A) Schematic representation of the chemoproteomics workflow. LH205 is used to enrich WDR5 from HEK293 lysate on Streptactin beads. In the competition setup an excess of LH168 is used to compete the binding, confirming the specificity of the interaction. (B) Assessment of the selectivity of LH168 through a chemoproteomics approach. LH205, a biotin-adduct of LH168 was used to enrich interaction partners (interaction partners = red, WDR5 = purple) of LH168 from HEK293 lysate. (C) Competition of LH205 by LH168 underscores the specific binding of LH168 to WDR5 (purple). The differential abundance was calculated using R and significance was determined across the replicates (*n* = 4, independent experiments) by using a two-tailed moderated *t*-test with multiple testing correction based on the Benjamini Hochberg method.

The probe candidate LH168 and the negative control LH222 were evaluated side by side in a comprehensive set of bioassays (Fig. 3 and 4), in some cases alongside the known WDR5 ligand OICR9429 as a positive control and reference compound. SPR and isothermal titration calorimetry (ITC) assays consistently confirmed the potent binding of LH168, with low nanomolar *K*_D_ values (*K*_D_ = 38 nM by ITC and 1.9 nM by SPR), whereas the negative control was inactive or exhibited only very weak binding. The thermodynamic parameters obtained from ITC experiment indicate that the LH168-WDR5 binding interaction is mainly driven by enthalpy (Δ*H* = −38.7 kJ mol^−1^) with a minor contribution of the entropy (*T*Δ*S* = 3.64 kJ mol^−1^ at 298 K). Interestingly, LH168, along with several other analogues containing the R^2^-substituent, displayed a long residence time, as demonstrated by kinetic SPR measurements. Strikingly, this contrasted with the less potent analogues (*S*)-MR43378 and 5b lacking the R^2^-substituent, as well as the published WDR5 ligand OICR9429, which belongs to a distinct chemotype (Table 2 and Fig. S2, ESI†).

To gain further insights into the WDR5-LH168 binding interaction and potential explanations for its slow binding behavior (slow *k*_off_ and *k*_on_ rates), we resolved the corresponding X-ray co-crystal structure (Table S2, ESI†) and compared it with both the original DEL–ML hit (*S*)-MR43378 and the known WDR5 inhibitor OICR9429 (Fig. 2). (*S*)-MR43378 and LH168 adopt similar binding pose, with the primary difference being a slight distortion of the central phenyl ring in LH168. This distortion is likely caused by an additional substituent that extends into the binding pocket, forming additional interactions. Notably, the overall shape of the binding pocket remains nearly unchanged. In contrast, there is a dramatic difference in the binding pose between LH168 and OICR9429, since they bind to very distinct WDR5 conformations (as illustrated by the Video in ESI†). We hypothesize that the prolonged residence time of LH168 is due to its ability to induce a more closed conformation of the protein, effectively filling the binding pocket, and forming a tight interaction with the WDR5 binding pocket. Unlike (*S*)-MR43378 or OICR9429, the dissociation of LH168 appears to require conformational rearrangement of the binding site, which presumably results in slow on- and off-rates.

LH168 and its negative control compound, LH222, were additionally profiled in the NanoBRET cell-based target engagement assay (Fig. 3). Gratifyingly, LH168 showed potent target engagement with an EC_50_ value of 10 nM (*n* = 12) in intact cells, demonstrating excellent cell membrane penetration. Consistent with the previous data, the negative control LH222 did not show any measurable binding to WDR5 in the NanoBRET assay with intact cells, while in permeabilized cells it showed more than a 150-fold difference in binding affinity (EC_50_) compared to the chemical probe compound LH168.

To probe the selectivity, LH168 was tested against a panel of methyltransferases and methyl-lysine and methyl-arginine binders. Specifically, as depicted in Fig. 3, LH168 was profiled at 50 μM against 13 histone/arginine methyltransferases using radioactivity-based assay as well as against WDR5 and seven arginine binders in DSF assay. Both assays revealed high selectivity of LH168 for WDR5.

The effect of LH168, LH222 and OICR9429 on cell viability of MV4-11 (frequently found to be sensitive to WDR5 loss) and K562 (not sensitive to WDR5 loss) cells was assessed using the CellTiterGlo assay. In contrast to previously reported WIN-site WDR5 inhibitors that reduce viability of MV4-11 cells,^15^ we did not observe any cytotoxity up to a concentration of 1 μM (Fig. S3, ESI†). The cytotoxicity observed at higher concentrations of LH168 in both MV4-11 and K562 cells was likely due to unspecific effects unrelated to WDR5 inhibition, as a similar toxicity was observed with the negative control LH222. In comparison, the chemical probe OICR9429, which served as a control in these assays also showed minimal toxicity across the tested concentration range. Notably, MV4-11 and K562 cells exhibited comparable sensitivity profiles, with MV4-11 displaying only slightly increased sensitivity to inhibitor treatment.

**Scheme 3:**
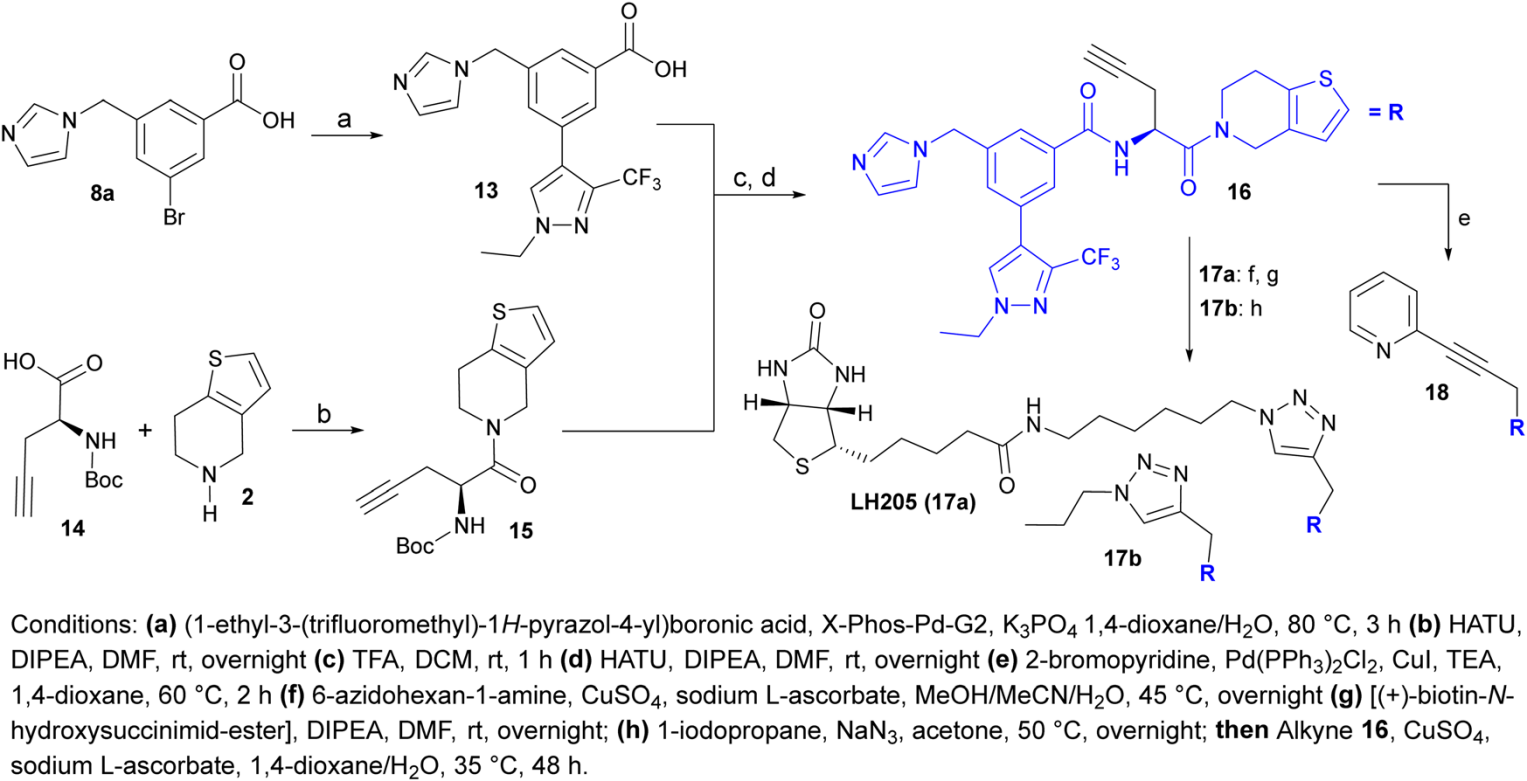
Synthesis of alkyne 16 and its further derivatization providing biotinylated compound LH205 (17a), 17b and 18.

Encouraged by these results, we decided to synthesize a biotinylated LH168 analogue that would allow assessment of proteome-wide selectivity by MS-proteomics. Exploiting the structural insights into the binding mode from the co-crystal structure, we synthesized analogue 16 bearing a solvent exposed alkyne functionality instead of aliphatic chain (Scheme 3). The alkyne analogue 16 proved to be readily accessible from inexpensive chemicals and was envisioned to be a convenient synthetic building block, for instance for click chemistry, and therefore well suited for attachment of linkers and development of bifunctional modalities.

*Via* Cu-catalyzed click reaction and Sonogashira coupling we synthesized compounds 17a (LH205), 17b and 18 to probe the chemistry as well as the effect of the attached moieties on the *in vitro* binding affinity and cellular activity. While the binding potency of 17b and 18 to isolated WDR5 protein is only slightly lower than for LH168, as determined by SPR, the *in cellulo* binding is diminished which is likely due to reduced cell penetration (Table 3 and Scheme 3). Due to the presence of biotin moiety, compound 17a was not evaluated in the SPR experiment as it would likely result in interference due to biotin binding to the chip.

**Table 3:** Potency of compounds 17a, 17b and 18 as determined by NanoBRET and SPR (kinetic fit)

Compound	EC_50_ NanoBRET [nM]		*K*_D_ SPR [nM]	Residence time [s]
	Intact	Permeabilized		
LH205 (17a)	6151	2854	ND	ND
17b	659	792	3.9	385^a^
18	743	1674	19.9	174

a Value with asterisk was obtained from dissociation curves that didn’t reach baseline due to slow off-rate, which might negatively affect precise calculation of residence time.

To further investigate the selectivity of LH168 in the cellular context, the biotinylated compound LH205 was incubated with HEK293 lysate and interacting proteins were pulled down using Streptactin beads and analyzed by label-free proteomics. In the presence of LH205, endogenously biotinylated proteins such as PCCA were depleted, indicating a successful immobilization on the Streptactin matrix. WDR5 was found to be one of the most strongly enriched proteins. No significant enrichment of other components of WDR5 containing complexes was observed, suggesting that only WDR5 protein that was not associated with chromatin was enriched or that the complex was not sufficiently stable to allow enrichment by this method. In addition, it is important to consider that LH168 acts as a protein–protein interaction inhibitor which further abrogated enrichment of intera ction partners dependent on binding to the WIN-site. Since several other proteins were significantly enriched alongside WDR5, we set up a competition experiment where the original probe LH168 was added in excess. In this setup, only specific interactors of LH168 are expected to be depleted. Gratifyingly, only WDR5 was significantly depleted in the protein fraction pulled down by the Streptactin beads, demonstrating the exquisite selectivity of LH168 for WDR5 (Fig. 4).

## Conclusions

While several small-molecule ligands targeting the druggable WIN and WBM pockets of WDR5 have been reported, the number of well-characterized tool compounds available for studying WDR5 biology remains limited.

Herein, we describe the rapid optimization of the DEL–ML hit MR43378 into LH168, a high-quality chemical probe that selectively targets the WIN-site pocket of the WDR5 protein. This work highlights the power of the DEL–ML platform in identifying small molecules that can serve as convenient starting points for developing tool compounds or leads in drug discovery.

LH168 exhibits an approximately 10-fold increase in cellular binding potency and a significantly longer residence time compared to the state-of-the-art WDR5 chemical probe OICR9429. X-ray structure reveals that both LH168 and OICR9429 bind to distinct conformations of WDR5 WIN site. In this respect, the combination of the chemically distinct LH168 and OICR9429 molecules provides not only chemical orthogonality but also complementarity *via* stabilization of the WIN site in different conformations. Importantly, LH168 is highly selective for WDR5, as demonstrated by pull-down experiments and screening against a panel of functionally related proteins. Based on these findings, we recommend using LH168 at concentrations up to 1 μM for cell-based experiments.

Additionally, the scientific community can take advantage of the alkyne derivative 16 for the design and synthesis of bifunctional molecules. Given the long residence time of LH168 and its analogues, development of degraders derived from LH168 represents an interesting direction for future research, enabling investigation kinetic effects on degradation potency.

In conclusion, the chemical probe LH168, the negative control LH222, and the alkyne analogue 16 represent a high-quality set of tool compounds that complement previously published chemical probes.

## Author contributions

LH performed the chemical synthesis and analytical characterization of compounds. CL analyzed final compounds by SPR. FF performed DSF measurements, pull-downs, and chemoproteomics. JD performed ITC experiments. S. Keller performed pull-downs and chemoproteomics. MPS performed the NanoBRET assay. HH conducted viability assays (CellTiter-Glo). AK expressed and purified WDR5 protein for *in vitro* assays. FL and IC performed radioactivity assay and DSF selectivity screening. SWK purified WDR5 protein for crystallographic experiments and crystallized. AD refined the structure and deposited. L Halabelian supervised crystallographic experiments. MG supervised pull-down and chemoproteomics experiments. SM supervised NanoBRET experiments. S. Knapp supervised the scientific project and edited the manuscript. VN supervised chemical design and synthesis, coordinated manuscript writing, biological testing and scientific collaboration. All authors contributed to the assembly of scientific data and ESI.†

## Conflicts of interest

The Authors declare no conflict of interest.

## Supplementary Material

CB-OLF-D5CB00109A-s001

CB-OLF-D5CB00109A-s002

## Data Availability

The data supporting this article have been included as part of the ESI.† Crystallographic data for LH168-WDR5 has been deposited at the PBD under PDB ID: 9D5Z and can be obtained from https://doi.org/10.2210/pdb9D5Z/pdb. The mass spectrometry proteomics data have been deposited to the ProteomeXchange Consortium (https://proteomecentral.proteomexchange.org) *via* the PRIDE partner repository with the dataset identifier PXD063455.

## References

[cit1] Trievel R. C., Shilatifard A. (2009). Nat. Struct. Mol. Biol..

[cit2] Thomas L. R., Wang Q., Grieb B. C., Phan J., Foshage A. M., Sun Q., Olejniczak E. T., Clark T., Dey S., Lorey S., Alicie B., Howard G. C., Cawthon B., Ess K. C., Eischen C. M., Zhao Z., Fesik S. W., Tansey W. P. (2015). Mol. Cell.

[cit3] Mitchell K., Sprowls S. A., Arora S., Shakya S., Silver D. J., Goins C. M., Wallace L., Roversi G., Schafer R. E., Kay K., Miller T. E., Lauko A., Bassett J., Kashyap A., D’Amato Kass J., Mulkearns-Hubert E. E., Johnson S., Alvarado J., Rich J. N., Holland E. C., Paddison P. J., Patel A. P., Stauffer S. R., Hubert C. G., Lathia J. D. (2023). Genes Dev..

[cit4] Yu X., Li D., Kottur J., Shen Y., Kim H. S., Park K.-S., Tsai Y.-H., Gong W., Wang J., Suzuki K., Parker J., Herring L., Kaniskan H. Ü., Cai L., Jain R., Liu J., Aggarwal A. K., Wang G. G., Jin J. (2021). Sci. Transl. Med..

[cit5] Grebien F., Vedadi M., Getlik M., Giambruno R., Grover A., Avellino R., Skucha A., Vittori S., Kuznetsova E., Smil D., Barsyte-Lovejoy D., Li F., Poda G., Schapira M., Wu H., Dong A., Senisterra G., Stukalov A., Huber K. V. M., Schönegger A., Marcellus R., Bilban M., Bock C., Brown P. J., Zuber J., Bennett K. L., Al-awar R., Delwel R., Nerlov C., Arrowsmith C. H., Superti-Furga G. (2015). Nat. Chem. Biol..

[cit6] Karatas H., Li Y., Liu L., Ji J., Lee S., Chen Y., Yang J., Huang L., Bernard D., Xu J., Townsend E. C., Cao F., Ran X., Li X., Wen B., Sun D., Stuckey J. A., Lei M., Dou Y., Wang S. (2017). J. Med. Chem..

[cit7] Dölle A., Adhikari B., Krämer A., Weckesser J., Berner N., Berger L.-M., Diebold M., Szewczyk M. M., Barsyte-Lovejoy D., Arrowsmith C. H., Gebel J., Löhr F., Dötsch V., Eilers M., Heinzlmeir S., Kuster B., Sotriffer C., Wolf E., Knapp S. (2021). J. Med. Chem..

[cit8] Ding J., Li G., Liu H., Liu L., Lin Y., Gao J., Zhou G., Shen L., Zhao M., Yu Y., Guo W., Hommel U., Ottl J., Blank J., Aubin N., Wei Y., He H., Sage D. R., Atadja P. W., Li E., Jain R. K., Tallarico J. A., Canham S. M., Chiang Y.-L., Wang H. (2023). ACS Chem. Biol..

[cit9] Samanipour S., Barron L. P., Van Herwerden D., Praetorius A., Thomas K. V., O’Brien J. W. (2024). JACS Au.

[cit10] Lipinski C., Hopkins A. (2004). Nature.

[cit11] Gironda-Martínez A., Donckele E. J., Samain F., Neri D. (2021). ACS Pharmacol. Transl. Sci..

[cit12] Suo Y., Qian X., Xiong Z., Liu X., Wang C., Mu B., Wu X., Lu W., Cui M., Liu J., Chen Y., Zheng M., Lu X. (2024). J. Med. Chem..

[cit13] Ackloo S., Li F., Szewczyk M., Seitova A., Loppnau P., Zeng H., Xu J., Ahmad S., Arnautova Y. A., Baghaie A. J., Beldar S., Bolotokova A., Centrella P. A., Chau I., Clark M. A., Cuozzo J. W., Dehghani-Tafti S., Disch J. S., Dong A., Dumas A., Feng J. A., Ghiabi P., Gibson E., Gilmer J., Goldman B., Green S. R., Guié M.-A., Guilinger J. P., Harms N., Herasymenko O., Houliston S., Hutchinson A., Kearnes S., Keefe A. D., Kimani S. W., Kramer T., Kutera M., Kwak H. A., Lento C., Li Y., Liu J., Loup J., Machado R. A. C., Mulhern C. J., Perveen S., Righetto G. L., Riley P., Shrestha S., Sigel E. A., Silva M., Sintchak M. D., Slakman B. L., Taylor R. D., Thompson J., Torng W., Underkoffler C., Von Rechenberg M., Walsh R. T., Watson I., Wilson D. J., Wolf E., Yadav M., Yazdi A. K., Zhang J., Zhang Y., Santhakumar V., Edwards A. M., Barsyte-Lovejoy D., Schapira M., Brown P. J., Halabelian L., Arrowsmith C. H. (2025). J. Med. Chem..

[cit14] Tian J., Teuscher K. B., Aho E. R., Alvarado J. R., Mills J. J., Meyers K. M., Gogliotti R. D., Han C., Macdonald J. D., Sai J., Shaw J. G., Sensintaffar J. L., Zhao B., Rietz T. A., Thomas L. R., Payne W. G., Moore W. J., Stott G. M., Kondo J., Inoue M., Coffey R. J., Tansey W. P., Stauffer S. R., Lee T., Fesik S. W. (2020). J. Med. Chem..

[cit15] Teuscher K. B., Chowdhury S., Meyers K. M., Tian J., Sai J., Van Meveren M., South T. M., Sensintaffar J. L., Rietz T. A., Goswami S., Wang J., Grieb B. C., Lorey S. L., Howard G. C., Liu Q., Moore W. J., Stott G. M., Tansey W. P., Lee T., Fesik S. W. (2023). Proc. Natl. Acad. Sci. U. S. A..

[cit16] Patel A., Vought V. E., Dharmarajan V., Cosgrove M. S. (2008). J. Biol. Chem..

[cit17] Schwalm M. P., Krämer A., Dölle A., Weckesser J., Yu X., Jin J., Saxena K., Knapp S. (2023). Cell Chem. Biol..

