# Supplementary material for: Discovery of an exquisitely selective WDR5 chemical probe accelerated by a high-quality DEL–ML Hit
Source: RSC Chem Biol. 2025 Jul 17;6(10):1585–94. doi: 10.1039/d5cb00109a (PMC12415533; doi:10.1039/d5cb00109a)
Supplement: CB-006-D5CB00109A-s001 [file CB-006-D5CB00109A-s001.pdf]

Supplementary Information (SI) for RSC Chemical Biology.
This journal is © The Royal Society of Chemistry 2025

Supporting Information

Discovery of an Exquisitely Selective WDR5 Chemical Probe Accelerated by a High-Quality DEL-ML Hit

Lasse Hoffmann[1,2], Christopher Lenz[1,2], Frederic Farges[1,2], Serah W. Kimani[3], Johannes Dopfer[1,2], Sabrina Keller[4], Martin Peter Schwalm[1,2,5], Hanna Holzmann[1,2], Andreas Kraemer[1,2], Aiping Dong[3], Fengling Li[3], Irene Chau[6], Levon Halabelian[3,7,8], Matthias Gstaiger[4], Susanne Müller[1,2,5], Stefan Knapp[1,2,5*], Václav Němec[1,2*]

[1]Institute for Pharmaceutical Chemistry, Johann Wolfgang Goethe-University, Max-von-Laue-Str. 9, D-60438 Frankfurt am Main, Germany

[2]Structural Genomics Consortium, Buchmann Institute for Molecular Life Sciences, Johann Wolfgang Goethe-University, Max-von-Laue-Str. 15, D-60438 Frankfurt am Main, Germany

[3]Structural Genomics Consortium, University of Toronto, Toronto, ON, Canada

[4]Inst. f. Molekulare Systembiologie, Otto-Stern-Weg 3, 8093 Zürich, Switzerland

[5]German Cancer Consortium (DKTK), German Cancer Research Center (DKFZ), DKTK site Frankfurt-Mainz, 69120 Heidelberg, Germany

[6]Radiant Biotherapeutics Inc., 686 Bay St PGCRL, 21 9430.X, Toronto, ON, Canada

[7]Department of Pharmacology and Toxicology, University of Toronto, Toronto, ON, Canada

[8]Princess Margaret Cancer Centre, University Health Network, Toronto, ON, Canada

*Authors for correspondence: nemec@pharmchem.uni-frankfurt.de or knapp@pharmchem.uni-franfurt.de

1. Supplementary Tables and Figures

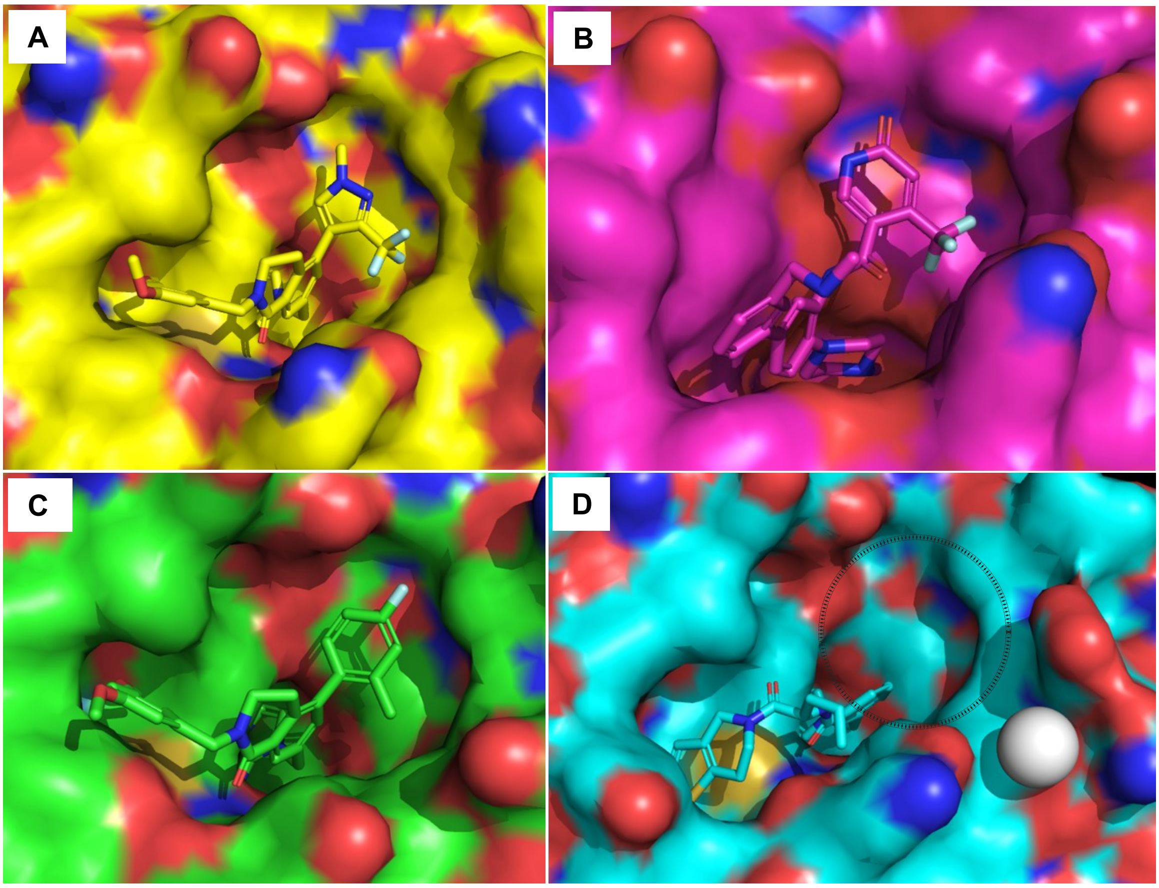

Figure S1: Comparison of the binding modes of three published WDR5 binders (A-C) and DEL-ML hit MR43378 (D). PDB ID: 7UpY (A), 4QL1 (B), 6UCS (C), 8T5I (D). The pocket which was targeted by the R^2-substituent is highlighted by a black circle.

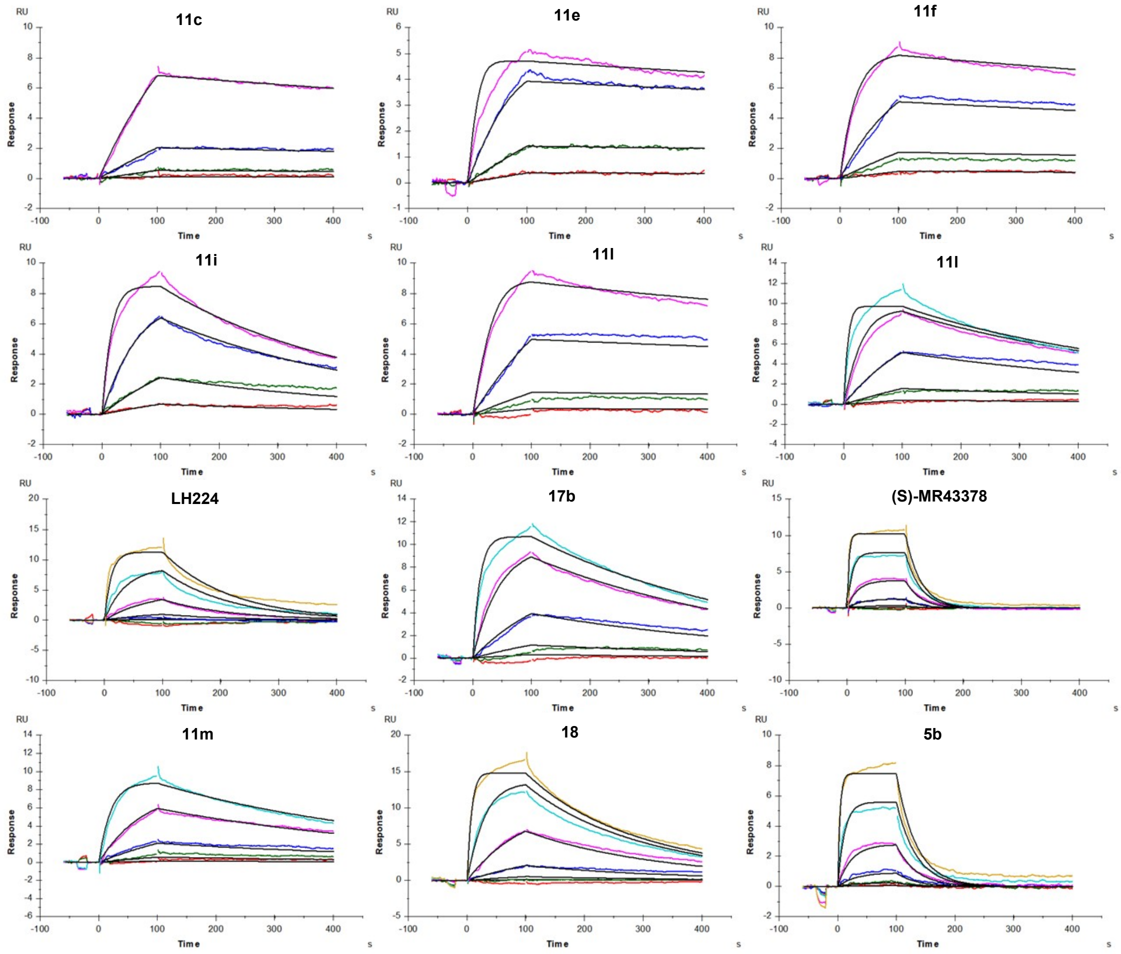

Figure S2: Representative SPR sensorgram overlay plots for compounds **11c**, **11e**, **11f**, **11i**, **11l**, **LH224**, **17b**, **(S)-MR43378**, **11m**, **18** and **5b** injected at increasing concentrations over immobilized WDR5. The data shown are referenced, blank-subtracted, and solvent-corrected. Kinetic fits are overlaid as black curves.

Compound	Average	SD ka	Average kd	SD kd	Average KD	SD KD	Residence time

	ka (1/Ms)	(1/Ms)	(1/s)	(1/s)	(M)	(M)	(s)
LH224	1.45E+05	2.67E+04	8.80E-03	3.32E-04	6.28E-08	1.25E-08	114
17b*	6.71E+05	4.34E+04	2.60E-03	1.07E-04	3.90E-09	4.31E-10	385
11n*	1.30E+06	3.02E+05	2.19E-03	1.19E-04	1.78E-09	4.29E-10	456
18	2.95E+05	3.68E+04	5.74E-03	2.30E-04	1.99E-08	3.40E-09	174
5b	8.31E+05	2.83E+05	3.85E-02	7.72E-03	4.95E-08	9.80E-09	26
(S)-MR43378	8.10E+05	1.78E+05	6.56E-02	2.98E-02	7.73E-08	1.82E-08	15
OICR9429	2.69E+06	2.83E+05	2.28E-02	9.42E-04	8.60E-09	1.28E-09	44
11m*	4.68E+05	2.74E+04	2.53E-03	2.41E-04	5.39E-09	3.70E-10	396
11c*	1.05E+06	6.51E+05	8.11E-04	2.74E-04	1.34E-09	9.53E-10	1233
11i	4.44E+06	1.55E+06	3.54E-03	5.03E-04	8.89E-10	2.69E-10	282
11l*	8.54E+06	7.62E+06	9.43E-04	3.60E-04	1.83E-10	8.29E-11	1060
11f*	6.11E+06	5.13E+06	5.46E-04	1.36E-04	1.71E-10	1.17E-10	1830
11e*	2.71E+06	7.85E+05	3.88E-04	4.03E-05	1.64E-10	7.37E-11	2580
LH168	7.49E+05	3.97E+04	1.40E-03	1.36E-04	1.87E-09	1.68E-10	714

Table S1: Results from SPR measurements. KD values were determined using a kinetic 1:1 Langmuir interaction fit and are depicted as mean ± SD (n = 3). Confidence interval at α = 0.05. *Values for compounds marked with an asterisk were obtained from dissociation curves that did not reach baseline due to a slow off-rate, which may affect the accuracy of calculations.

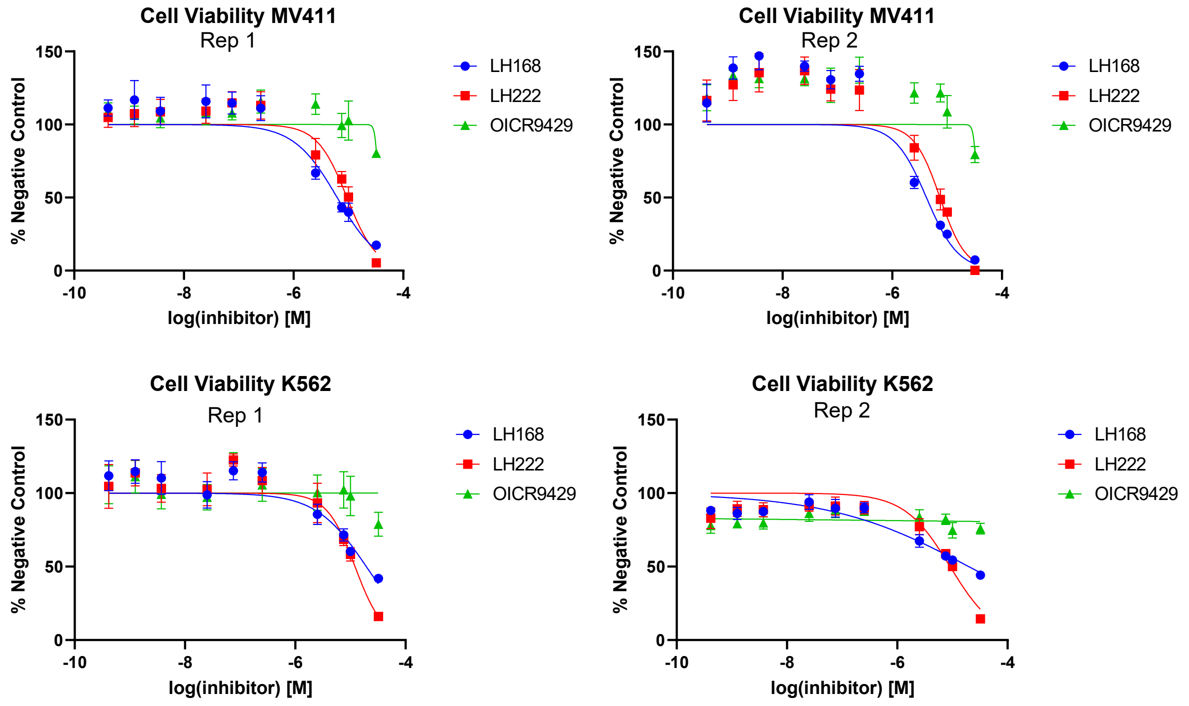

Figure S3: CellTiterGlo – cell viability assay. LH168, LH222 and OICR8429 were incubated with K562 or MV4-11 cells for 5 days.

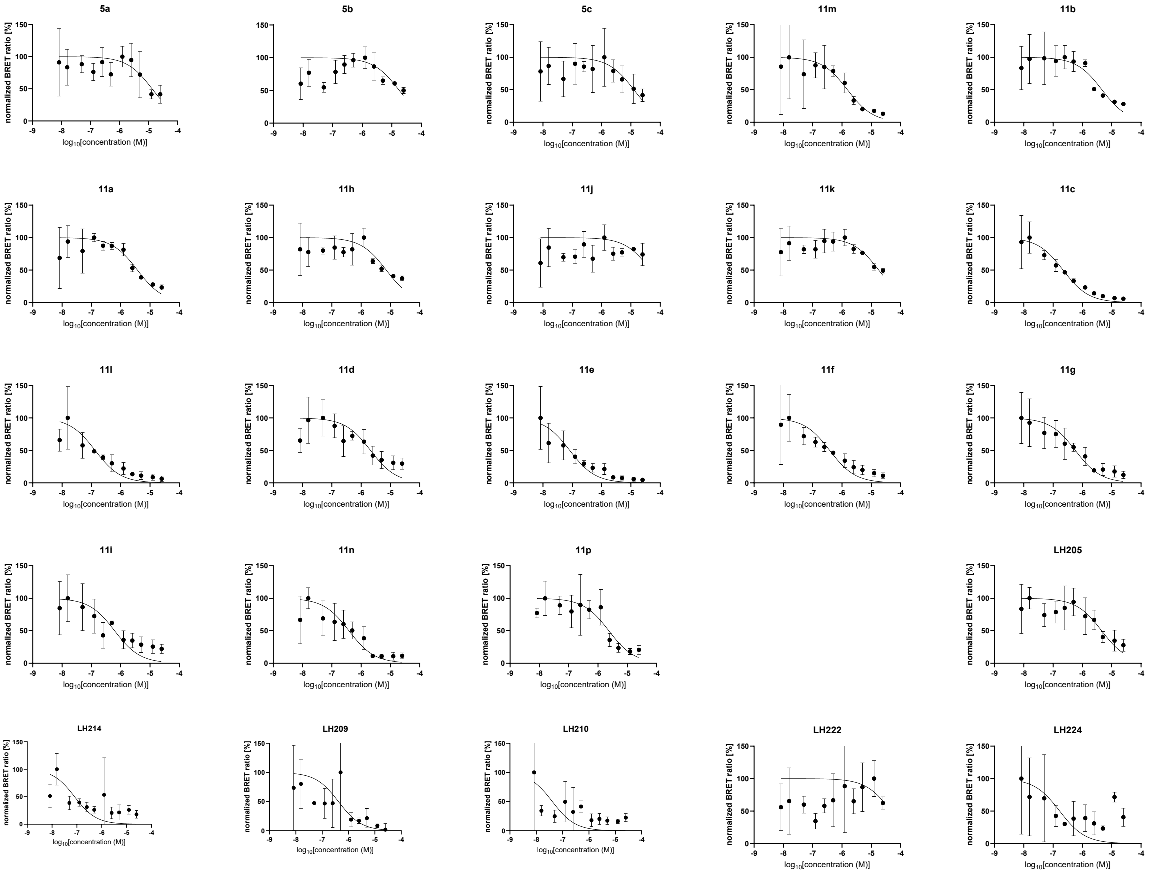

Figure S4: NanoBRET target engagement profiling in HEK293 cells, measured in intact cells (n=3).

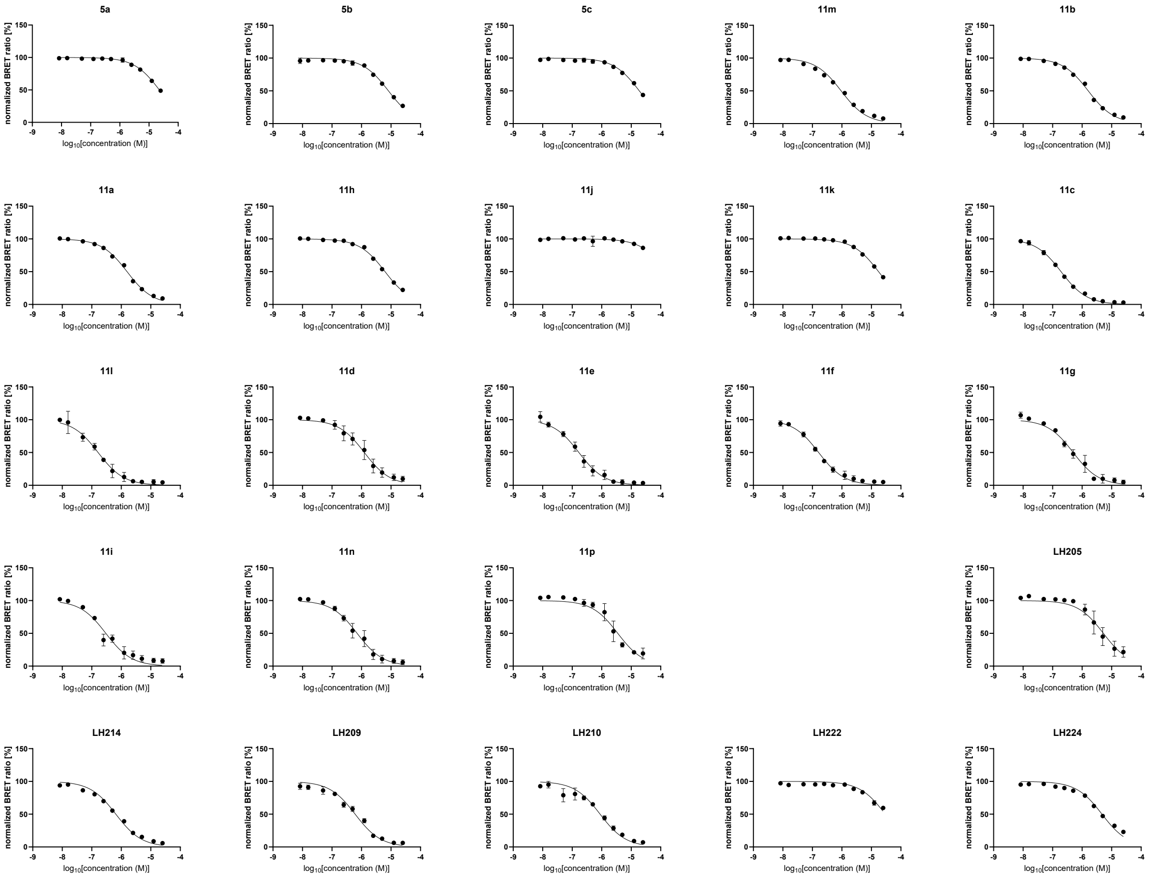

Figure S5: NanoBRET target engagement profiling in HEK293 cells, measured in digitonin-permeabilized cells (n=3).

Compound	NanoBRET $logEC_{50}$	
	Intact	Perm
**5a**	-5.257 ± 0.159	-4.644 ± 0.013
**5b**	-4.85 ± 0.06	-5.087 ± 0.014
**5c**	-5.107 ± 0.123	-4.726 ± 0.019
**11m**	-5.956 ± 0.283	-6.027 ± 0.022
**11b**	-5.596 ± 0.086	-5.794 ± 0.016
**11a**	-5.577 ± 0.182	-5.803 ± 0.014
**11h**	-5.284 ± 0.128	-5.2 ± 0.014
**11j**	-4.457 ± 0.294	-3.805 ± 0.03
**11k**	-4.806 ± 0.058	-4.757 ± 0.019
**11c**	-6.711 ± 0.246	-6.714 ± 0.025
**11l**	-6.659 ± 0.483	-6.783 ± 0.022
**11d**	-5.79 ± 0.465	-5.896 ± 0.16
**11e**	-6.815 ± 0.556	-6.77 ± 0.086
**11f**	-6.507 ± 0.459	-6.786 ± 0.057
**11g**	-6.282 ± 0.306	-6.298 ± 0.06
**11i**	-5.934 ± 0.411	-6.521 ± 0.018
**11n**	-6.512 ± 0.277	-6.142 ± 0.123
**LH168 (n=12)**	-8.001 ± 0.068	-6.813 ± 0.027
**11p**	-5.743 ± 0.135	-5.457 ± 0.12

**17a**	-5.425 ± 0.169	-5.274 ± 0.139
**LH214**	-7.464 ± 0.271	-6.183 ± 0.024
**17b**	-7.297 ± 0.812	-6.215 ± 0.046
**18**	-6.6 ± 1.35	-6.054 ± 0.011
**LH222**	-5.015 ± 1.27	-4.528 ± 0.035
**LH224**	-7.452 ± 0.371	-5.327 ± 0.01

Table S2: NanoBRET $\log_{10}EC_{50}$ calculated using data depicted in Figures S4 and S5. For LH168: n=12, for other compounds: n=3. Confidence interval at α = 0.05.

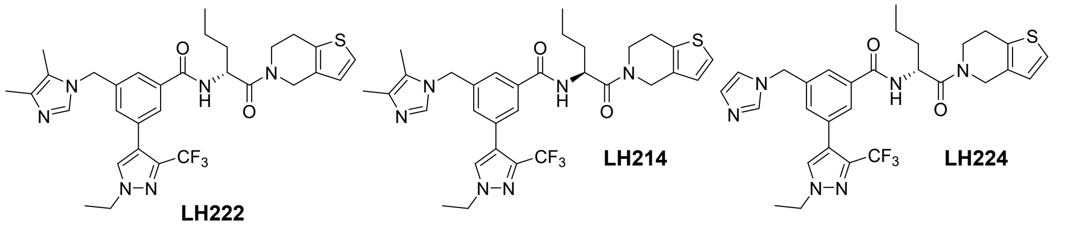

LH214 **LH224**

LH222
(negative control compound)

Figure S6: To find a negative control compound which is structurally similar to LH168, we synthesized compounds LH214, LH222 and LH224. Only LH222 was sufficiently inactive (Table S1 and S2).

2. Material and Methods

Isothermal titration calorimetry (ITC)

Isothermal titration calorimetry experiments were performed using a NanoITC instrument (TA Instruments, New Castle, USA) at 25 °C with gel filtration buffer (20 mM HEPES, pH 7.5, 150 mM NaCl and 1 mM TCEP). To minimise non-specific thermal effects of dilution, the DMSO concentration in both protein and compound samples was adjusted to 1% in all experiments. LH168 was measured at a concentration of 15 µM against 120 µM protein and LH222 was measured at 15 µM against 225 µM protein using a reverse titration setup. In this protocol, the sample chamber was pre-equilibrated with the compound while WDR5-avi protein was titrated into the chamber and the exothermic heat evolution was continuously monitored. The binding heat was integrated, baseline-corrected, and analysed using a single-site binding model following the manufacturer's guidelines. Thermodynamic parameters, including the enthalpy change (ΔH), entropy change (TΔS), equilibrium association constant (KA), dissociation constant (KD), and binding stoichiometry (n), were calculated with TA Instruments NanoAnalyze software. Graphical data visualizations were generated using GraphPad Prism version 9.3.

Surface plasmon resonance (SPR)

SPR analyses were conducted using a Biacore T200 instrument at 25°C. The running buffer consisted of 30 mM HEPES (pH 7.4), 250 mM NaCl, 0.5 mM TCEP, 0.05% (v/v) Tween 20, and 2.6% (v/v) DMSO. Recombinant biotinylated WDR5-Avi protein was diluted to 10 µg/mL in the running buffer and immobilized onto flow cells 2–4 of a Series S Sensor Chip CM5 (Cytiva) via the standard amine coupling protocol, at a flow rate of 10 µL/min. Surface activation was performed using 483 mM EDC and 10 mM NHS, followed by deactivation with 1 M ethanolamine. Protein immobilization achieved final response units (RU) of 1800–1900. Flow cell 1 was left as an empty reference surface.

Compounds were analyzed as triplicates under multi-cycle conditions at a flow rate of 30 µL/min. Serial injections included 4 – 11 concentrations ranging from 1 nM – 2 µM. Sensorgrams were referenced, blank-subtracted and solvent-corrected using the Biacore T200 Evaluation Software version 3.2.1. Final sensorgrams for LH168, OICR9429 and LH222 were replotted using GraphPad Prism 8.0.1.

Differential scanning fluorimetry (compounds 5a-c)

To measure changes in the melting temperature of WDR5 in the presence of compounds, differential scanning fluorimetry was performed. For this, recombinantly expressed WDR5 was diluted to 10 µM in assay buffer (25 mM HEPES pH 7.5, 150 mM NaCl, 2 mM TCEP) containing SYPRO orange (Thermo Fisher, final concentration 5x). Then, compounds were added to a final concentration of 50 µM (2% DMSO). Additionally, a DMSO control was included for referencing. Measurements were performed in a 384-well format with a QuantStudio 5 Real-Time PCR system (applied biosystems) and fluorescence was measured at temperatures ranging from 25°C to 95°C (filter settings: excitation 465 nm and emission 590 nm). The resulting data were analysed in the Protein Thermal Shift software (applied biosystems) and the melting point was determined through Boltzmann fitting.

Differential scanning fluorimetry (selectivity panel)

WDR5 and seven other proteins containing PHD or Tudor domains (FXR1, SGF29, SND1, TDRD3, SETDB1-TTD, UHRF1-139-298 and UHRF1-147-380) were diluted to 0.1 mg/mL in assay buffer (100 mM HEPES, 150 mM NaCl, pH 7.5) containing 5× SYPRO Orange dye (Life Technologies, S-6650). Reactions were assembled in a final volume of 20 µL in white 384-well polypropylene plates, containing either 50 µM LH168 or serial dilutions of LH168 up to 50 µM. DSF was conducted using a LightCycler 480 II instrument (Roche Applied Science, Penzberg, Germany) with a temperature gradient of 4°C/min from 20°C to 95°C. Fluorescence data were collected at 0.5°C intervals. Melting temperatures (T_m) were determined by fitting the fluorescence data to a Boltzmann sigmoid function, as previously described.[1]

RA Selectivity Assay

The effect of LH168 on the methyltransferase activity of G9a, SETD7, PRDM9, the MLL1 and MLL3 pentameric complexes, PRMT1, PRMT3, PRMT4, the PRMT5–MEP50 complex, PRMT6, PRMT7, PRMT8, and PRMT9 was assessed by monitoring the incorporation of tritium-labeled methyl groups into lysine or arginine residues of peptide substrates using the Scintillation Proximity Assay (SPA) as previously described.[2]

Chemoproteomics

HEK293 cells (ATCC CRL-1573) were grown to confluency in 15 cm dishes. The cells were detached with 10 mL phosphate buffered saline (1x PBS) containing 1 mM EDTA. After washing the cells once with 1x PBS (1 mM EDTA) and removing the supernatant, they were snap frozen in liquid nitrogen and stored at -80°C. Cells were lysed by resuspension in HNN lysis buffer (50 mM HEPES pH 8.0, 150 mM NaCl, 50 mM NaF, 0.5% IGPAL-630, 400 nM Na_3VO_4, 1 mM PMSF and 0.2% protease inhibitors (Sigma). Following 10 minutes of incubation on ice, the lysate was centrifuged for 30 minutes at 18.000*g*. After pooling the cleared lysate, it was distributed into 1900 µL aliquots per sample (equivalent to 2x 15 cm dishes). 50 µM of LH168 or DMSO together with 5 µM LH205 or DMSO were added to the individual samples with 4 replicates per condition and incubated for 1 hour at 4°C on a rotating wheel. Next, the treated lysate was added to 80 µL of Strep-Tactin beads (50% slurry, IBA Lifesciences) previously modified to be resistant to tryptic cleavage[3] followed by incubation of the samples on a rotating wheel for 1 hour at 4°C. After the transfer of the beads to a 96-well 1 µm glass filter plate (Pall corporation), the residual lysate was filtered away and the beads washed with two times HNN lysis buffer without protease inhibitors and PMSF, twice with 1 mL of HNN lysis buffer without protease inhibitors, PMSF and IGPAL-630 and twice with 1 mL of 100 mM ammonium bicarbonate (ABC). The beads were resuspended in 100 mM ABC and transferred to a 10 kDa cutoff plate (Pall corporation). The supernatant was removed via centrifugation at 1500*g* for 15 minutes. The samples were resuspended in 8 M Urea in 100 mM ABC, reduced with 10 mM TCEP (40 minutes, 37°C and 200 rpm) and alkylated with 20 mM Iodoacetamide (30 minutes, 37°C and 200 rpm). The 8 M Urea was removed by centrifugation at 1500g for 15 minutes, followed by two wash steps with 100 mM ABC, where the centrifugation time was increased to 30 minutes after the last wash. The beads were resuspended in 203 µL 100 mM ABC containing 1 µg of trypsin (Promega) and 0.5 µg of LysC (FUJIFLM Wako) for overnight digestion (37°C, 200 rpm). The supernatant containing the peptides was collected via centrifugation at 1500*g* for 20 minutes and remaining peptides were eluted in 100 µL of 100 mM ABC. Both solutions were pooled and acidified through the addition of formic acid (FA) to a final concentration of 5%. The peptides were loaded on a equilibrated 96-well C18 plate (Nest group, HNFR S18V) via centrifugation (1500*g*, 1 min), washed three times with 200 µL of 5% Acetonitrile (ACN) with 0.1% FA and finally eluted with 2x 100 µL of 50% ACN with 0.1% FA into a fresh collection plate. Peptides were dried in the speed vac and resuspended in 20 µL of 2% ACN with 0.1% FA and 0.2x iRT standard (Biognosys). Samples were injected into a Waters nanoACQUITY coupled to an Orbitrap Fusion Lumos. In the samples, where the intensity in the chromatogram deviated from the other replicate, the injection volume was adjusted (for injection volumes see table S4). The peptides were separated on a 30 cm column packed with 3 µm C18 resin (Dr. Maisch) by a 120 min gradient from 3% to 35% buffer B (100% ACN containing 0.1% FA) at a flow rate of 300 nL/min. Each sample was measured in data-independent acquisition (DIA) and data-dependent acquisition (DDA) mode. The DDA mode was performed with the following parameters: the full scan range was 350 – 1'150m/z at 120'000 resolutions. The data dependent scans were acquired within a cycle time of 3 sec. Only charge states between or equal to 2 – 7 were included. Fragmentation was obtained with HCD collision energy 30%. The MS2 spectra were measured with an Orbitrap resolution of 30'000 with isolation windows of 1.6 m/z. The normalized AGC target was set to 200% with maximum injection time of 54 msec. The DIA mode was performed with the following parameters: the scan range was 350 – 1400 at 120'000 resolutions. The normalized AGC target was set to 50% with a maximum injection time of 100 msec. The RF lens was set to 30%. The targeted MS2 spectra for the desired masses in the variable isolation windows together with the normalized AGC target percentage listed below were acquired by fragmentation with a HCD of 28%. The Orbitrap resolution was 30'000 with variable scan ranges. The maximum injection time was set to 54 msec and the RF lens to 30%. A hybrid spectral library was generated from all DDA and DIA runs, using Pulsar search engine in Biognosys Spectronaut v.19.1. The standard settings were used except that LysC/P was added to the cleavage rules. The DIA runs were searched in Biognosys Spectronaut v.19.1 against the generated library with the default setting with the following changes: Cross-Run Normalization was unselected and Used Biognosys' iRT Kit was selected. The precursor-level data were exported and further processed in protti version 0.9 R version 4.4.1. Data normalization was performed on the precursor level and protein abundances were calculated for proteins with at least three observed precursors in each sample. Following differential abundance calculation, significance was determined using a moderated t-test with Benjamini-

Hochberg multiple testing correction. Changes in protein abundances were considered significant, if the absolute fold change (log_2) is greater than one unit and the adjusted p values are below 0.05.

Number	Mass to charge ratio (m/z)	Charge (z)	Isolation window width ((m/z)
1	358	2	16
2	373	2	16
3	388	2	16
4	403	2	16
5	418	2	16
6	433	2	16
7	448	2	16
8	463	2	16
9	478	2	16
10	493	2	16
11	508	2	16
12	523	2	16
13	538	2	16
14	553	2	16
15	568	2	16
16	583	2	16
17	598	2	16
18	613	2	16
19	628	2	16
20	643	2	16
21	659	2	18
22	676	2	18
23	693	2	18
24	710	2	18
25	727	2	18
26	744	2	18
27	761	2	18
28	778	2	18
29	795	2	18
30	813	2	20
31	832	2	20
32	851	2	20
33	870	2	20
34	889	2	20
35	908	2	20
36	929.5	2	25
37	953.5	2	25
38	977.5	2	25
39	1006.5	2	35
40	1048	2	50
41	1111	2	78

Table S3: DIA isolation window settings used for the chemoproteomics experiments.

**Sample Name**	**Inj Vol**
Blank	1
HEK293 treated with DMSO Replicate 1	3
HEK293 treated with DMSO Replicate 2	3
HEK293 treated with DMSO Replicate 3	3
HEK293 treated with DMSO Replicate 4	3
Blank	1
HEK293 treated with LH205 Replicate 1	3
HEK293 treated with LH205 Replicate 2	3
HEK293 treated with LH205 Replicate 3	3
HEK293 treated with LH205 Replicate 4	3
Blank	1
HEK293 treated with LH205+LH168 Replicate 1	3
HEK293 treated with LH205+LH168 Replicate 2	3
HEK293 treated with LH205+LH168 Replicate 3	3
HEK293 treated with LH205+LH168 Replicate 3	3
Blank	1
HEK293 treated with DMSO Replicate 1	3
HEK293 treated with DMSO Replicate 2	3
HEK293 treated with DMSO Replicate 3	3
HEK293 treated with DMSO Replicate 4	3
Blank	1
HEK293 treated with LH205 Replicate 1	3
HEK293 treated with LH205 Replicate 2	1.7
HEK293 treated with LH205 Replicate 3	1.8
HEK293 treated with LH205 Replicate 4	3
Blank	1
HEK293 treated with LH205+LH168 Replicate 1	3
HEK293 treated with LH205+LH168 Replicate 2	5
HEK293 treated with LH205+LH168 Replicate 3	3
HEK293 treated with LH205+LH168 Replicate 3	3
Blank	1
HEK293 treated with LH205 Replicate 2	1.7
HEK293 treated with LH205 Replicate 3	1.8

Blank	1
HEK293 treated with LH205+LH168 Replicate 2	5
Blank	1
HEK293 treated with LH205 Replicate 1	4.5
HEK293 treated with LH205 Replicate 4	4.5
Blank	1
Blank	1

Table S4: Injection volumes across the different samples measured in DDA and DIA mode. Volumes were adjusted to obtain similar overall chromatogram intensities

Cell Viability Assessment

K562 and MV411 cell lines were seeded at a density of 2000 cells per well in a 384 well plate (Greiner: 781207) and left overnight at 37°C and 5% CO_2. The following day LH168, LH222 and OICR9429 were titrated at various concentrations using the ECHO 550 Acoustic Liquid Handler (Labcyte) and incubated for 5 days at 37°C and 5% CO_2. Cell viability was assessed using the CellTiter-Glo® 2.0 Assay (Promega) in accordance to manufacturer's instructions, luminescence was measured via PHERAstar plate reader (BMG Labtech). Technical quadruplicates were averaged and background corrected. Averages were normalised against DMSO (100%) and wells containing no cells (0%). Values were plotted as "% Negative Control" against compound concentration and IC50s were calculated via non-linear regression, log(inhibitor) vs normalised response with variable slope, using the equation Y=100/(1+10^((LogIC50-X)*HillSlope)) (Graphpad Prism 9).

BRET assays for cellular target engagement[4]

Full-length WDR5 was obtained as plasmid cloned in frame with a terminal NanoLuc-fusion. Plasmids were transfected into HEK293T cells using FuGENE HD (Promega, E2312), and proteins were allowed to express for 20 h. Serially diluted inhibitor and WDR5 Tracer (TracerDB ID: T000006) at the Tracer K_D concentration taken from TracerDB (tracerdb.org)[5] was pipetted into white 384-well plates (Greiner 781207) using an ECHO acoustic dispenser (Labcyte). The corresponding protein-transfected cells were added and reseeded at a density of 2×10^5 cells/mL after trypsinization and resuspending in Opti-MEM without phenol red (Life Technologies). The system was allowed to equilibrate for 2 h at 37 °C at 5 % CO_2 prior to bioluminescence resonance energy transfer (BRET) measurements. To measure BRET, NanoBRET NanoGlo Substrate was added as per the manufacturer's protocol, and filtered luminescence was measured on a PHERAstar plate reader (BMG Labtech) equipped with a luminescence filter pair (450 nm BP filter (donor) and 610 nm LP filter (acceptor)). Competitive displacement data were then graphed using GraphPad Prism 9 software using a normalized 3-parameter curve fit with the following equation: Y = 100/(1 + 10(X − log IC50)).

Protein crystallography of the WDR5-LH168 complex

Protein expression and purification for crystallization:

The human WDR5 protein was expressed in *E. coli* using a fragment (residues 24-324) of the gene (UniProt ID: P61964), cloned into an in-house pET28-derived expression vector, pET28-MHL. This construct encoded an N-terminal His_6-tag followed by a TEV protease cleavage site. Protein expression was carried out overnight at 16 ˚C in *E. coli* BL21 (DE3) pRARE2 cells. The protein was purified by immobilized nickel ion affinity chromatography. Eluted fractions were incubated with the TEV protease overnight at 4 ˚ C to remove the His-tag, followed by reverse nickel affinity purification. The resulting protein was further purified by size exclusion chromatography using a HiLoad^{TM} 16/60 Superdex^{TM} 75 column (GE Healthcare) equilibrated in a buffer containing 20 mM Tris-HCl (pH 7.5), 150 mM NaCl and 2 mM TCEP. Fractions containing pure WDR5 were pooled and concentrated using 10 kDa cutoff spin concentrators (Millipore). The final protein concentration was determined using a NanoDrop UV-

Vis spectrophotometer (Thermo Scientific), using an extinction coefficient of 68,410 M^{-1} cm^{-1} calculated from the amino acid sequence with the Expasy ProtParam tool (https://web.expasy.org/protparam/).

Co-crystallization and structural determination:

To generate WDR5-LH168 co-crystals, purified WDR5 at 17.2 mg/mL (0.502 mM) was mixed with a 5-fold molar excess of LH168 (2.51 mM) and incubated at room temperature for 15 minutes before setting up crystallization trays. Crystallization was performed using the sitting drop vapor-diffusion method by mixing 1.5 µL of the protein-compound complex with 1.5 µL of reservoir solution, equilibrated over 1,000 µL of the same reservoir solution. Crystals appeared within 3 days at 18 °C in a solution containing 20% PEG3350 and 0.2 M ammonium formate.

Crystals were cryoprotected by briefly soaking in mother liquor supplemented with 10% ethylene glycol and 1 mM ligand, then flash-frozen in liquid nitrogen. X-ray diffraction data were collected at the CLSI beamline 08B1-1 at the Canadian Light Source. Diffraction data were processed with HKL-3000[6] and the structure was determined by molecular replacement in Phaser,[7] using a previously reported WDR5 structure (PDB ID: 3SMR) as the search model. Model building and refinement were carried out through iterative cycles of manual rebuilding in Coot[8] and refinement with REFMAC[9] within the CCP4 software suite.[10] The final structure was validated using MolProbity,[11] and molecular graphics were prepared with PyMOL (DeLano, W. & Schrödinger, L. PyMOL: *http://www.pymol.org/pymol*).

The structure has been deposited in the RCSB protein data bank under accession code PDB ID: 9D5Z.

Table S5. **Data collection and refinement statistics**

	WDR5-LH168
PDB code	9D5Z
**Data collection**	
Space group	P1
Cell dimensions□□	
a, *b*, *c* (Å)	46.95, 61.19, 64.61
□□□□□□□□□□□□□ (°)	110.4, 91.2, 112.2
Resolution (Å)	50.0-1.70(1.73-1.70) *
R_{sym} or R_{merge}	0.046 (0.316)
CC1/2	0.994(0.936)
I / □I	22.6(2.9)
Completeness (%)	94.1(80.6)
Redundancy	3.4(3.2)
**Refinement**	
Resolution (Å)	42.9-1.70(2.37-2.31)
No. reflections	26469
R_{work} / R_{free}(%)	13.9/18.9
No. atoms	5271
Protein	4693
Ligand/ion	82
Water	476
B-factors	26.3
Protein	25.3
Ligand/ion	25.4
Water	35.8
R.m.s. deviations	
Bond lengths (Å)	0.005
Bond angles (°)	1.305

*Values in parentheses are for highest-resolution shell.

3. Chemistry section

All commercial chemicals and solvents were used without further purification. All reactions were performed under inert atmosphere (Argon balloon). Reactions were monitored by thin layer chromatography (TLC) using silica-coated ALUGRAM® Xtra SIL G UV254 plates from Macherey Nagel as well as by liquid chromatography–mass spectrometry (LCMS). Synthesized compounds were purified on a PuriFlash Flash Column Chromatography System from Interchim using prepacked silica columns (PF-30SIHP-JP (30 µm), PF-50SIHP-JP (50 µm), PF-15C18HP (15 µm), or PF-30C18HP (30 µm)) or by preparative HPLC which was carried out on an Agilent 1260

Infinity II device equipped with an Eclipse XDB-C18 (Agilent, 21.2 x 250 mm, 7 µm) reversed phase column. A suitable gradient (flow rate 21 mL/min.) was used, with 0.1 % TFA in water (A) and 0.1 % TFA in acetonitrile (B), as a mobile phase. Microwave assisted reactions were carried out on Biotage Initiator+ system using 2 mL or 5 mL Microwave Reaction Vials.

The synthesized compounds were characterized by ^{1}H NMR and ^{13}C-NMR spectrometry. NMR spectra were measured in DMSO-d_6, $CDCl_3$ or MeOH-d_4 on a Bruker AV300, AV400, AV400HD, AV500 or DPX600 spectrometer. Solvent signals were calibrated and are reported relative to residual nondeuterated solvent signals DMSO-d_6 (^{1}H NMR = 2.500 ppm; ^{13}C NMR = 39.520 ppm), $CDCl_3$ (^{1}H NMR = 7.260 ppm; ^{13}C NMR =77.160 ppm) or MeOH-d_4 (^{1}H NMR = 4.870 ppm; ^{13}C NMR =49.000 ppm). Chemical shifts (δ) are reported in parts per million (ppm).

LCMS was carried out on Agilent 1260 Infinity II device (methods A-D) with a 1260 DAD HS detector (G7117C; 310 nm or 320 nm) and a LC/MSD device (G6125B, ESI pos. 100-1000). The compounds were analyzed on Poroshell 120 EC-C18 (Agilent, 3 x 150 mm, 2.7 µm) reversed phase column using 0.1 % formic acid in water (A) and 0.1 % formic acid in acetonitrile (B) with a 1260 MWD detector (G7165A; 320 nm).

LCMS-Methods:
Method A: 0 min. 5 % B - 2 min. 80 % B - 5 min. 95 % B - 7 min. 95 % B (flow rate of 0.6 mL/min.).
Method B: 0 min. 5 % B – 2.8 min. 75 % B – 7.2 min. 100 % B – 7 min. 100 % B (flow rate of 0.6 mL/min.).
Method C: 0 min. 5 % B – 0.4 min. 5 % B – 8 min. 100 % B – 10 min. 100 % B (flow rate of 0.6 mL/min.).
Method D: 0 min. 5 % B – 0.6 min. 5 % B – 4.2 min. 55 % B – 7.4 min. 90 % B – 9 min. 100 % B – 11 min. 100 % B (flow rate of 0.6 mL/min.).
UV-detection was performed at absorption band in the range 235-395 nm (320 +- 75 nm).

All final compounds which were profiled in bioassays had purity ≥ 95 % unless stated otherwise, as determined by LCMS or NMR.

Synthetic procedures:

General Procedure 1; Amide Coupling: Carboxylic acid (1.0 equiv.) was dissolved in DMF and then O-(7-Azabenzotriazol-1-yl)-N,N,N′,N′-tetramethyluronium-hexafluorphosphat (1.0 equiv.) and N,N-diisopropylethylamine (4.0 equiv.) were added, after stirring at room temperature for 15 min amine (1.0 equiv.) was added. After stirring over night at room temperature. The mixture was concentrated in vacuo and purified by RP-flash chromatography.

General Procedure 2; Suzuki Cross-Coupling: The representative aryl halide (1 equiv.), aryl boronic acid or pinacol ester (2 equiv.), K_3PO_3 (2.5 equiv.) and Pd catalyst (X-Phos Pd Gen2) (0.05 equiv.) were dissolved in 1,4-dioxane/H_2O (4:1). The reaction mixture was stirred under an argon atmosphere at 80 °C for 3 hours. Then, the mixture was mixed with water and extracted with CH_2Cl_2. The combined organic layers were washed with brine, dried over $MgSO_4$, filtered, and concentrated under reduced pressure. Intermediate compounds were purified by RP-flash chromatography. Compounds were purified by flash chromatography, followed by RP-flash chromatography.

General Procedure 3; Buchwald–Hartwig amination: The representative aryl halide (1 equiv), amine (1.2 equiv), Pd catalyst ($Pd_2(dba)_3$) (0.1 equiv), ligand (4,5-Bis-(diphenylphosphino)-9,9-dimethylxanthen) (0.1 equiv) and *t*-BuOK (2.5 equiv) were suspended in 1,4-dioxane. The mixture was placed under argon and was heated to 100 °C in microwave (intensity = high) for 150 min. The mixture was concentrated in vacuo and purified by flash chromatography followed by RP-flash chromatography.

tert-butyl (_S_)-(1-(6,7-dihydrothieno[3,2-_c_]pyridin-5(_4H_)-yl)-1-oxopentan-2-yl)carbamate [3]

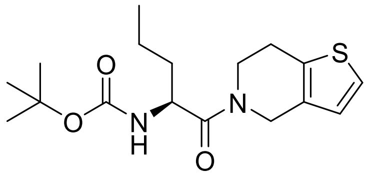

(*S*)-2-((*tert*-butoxycarbonyl)amino)pentanoic acid (1470 mg, 6.77 mmol) was reacted with 4,5,6,7-tetrahydrothieno[3,2-*c*]pyridine hydrochloride (942 mg, 6.77mmol) following **General Procedure 1** in DMF (30 mL) affording the title compound as a brownish solid (1580 mg, 4.67 mmol, 69 % yield).

^{1}H NMR (600 MHz, DMSO): δ 7.34 (d, *J* = 5.0 Hz, 1H), 7.01 (d, *J* = 8.2 Hz, 1H), 6.89 (t, *J* = 6.6 Hz, 1H), 4.70 – 4.63 (m, 1H), 4.57 (d, *J* = 15.7 Hz, 1H), 4.50 – 4.27 (m, 1H), 3.89 – 3.67 (m, 1H), 2.85 (d, *J* = 4.1 Hz, 1H), 2.75 (s, 1H), 1.60 – 1.17 (m, 11H), 1.36 (d, *J* = 8.6 Hz, 5H), 0.85 (dt, *J* = 15.8, 7.3 Hz, 2H).

LCMS (method B, ESI): R_t = 5.608 min, m/z = 361.1 [M + Na]$^+$.

(*S*)-*N*-(1-(6,7-dihydrothieno[3,2-*c*]pyridin-5(*4H*)-yl)-1-oxopentan-2-yl)-3-(hydroxymethyl)benzamide [4]

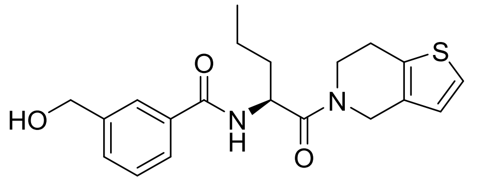

TFA (1.0 mL) was added to a solution of **3** (67 mg, 0.2 mmol) in DCM (2.3 mL). The reaction mixture was stirred at room temperature for 1 h, then was concentrated in vacuo. With no further purification **General Procedure 1** in DMF (8 mL) was followed using 3-(hydroxymethyl)benzoic acid (30 mg, 0.2 mmol), affording the title compound as a brownish solid (60 mg, 0.16 mmol, 82 % yield).

^{1}H NMR (400 MHz, CDCl3): δ 7.77 (s, 1H), 7.66 (d, J = 7.7 Hz, 1H), 7.50 (dd, J = 26.9, 7.7 Hz, 2H), 7.35 (dt, J = 13.1, 6.6 Hz, 1H), 7.16 (t, J = 5.0 Hz, 1H), 6.82 (dd, J = 5.1, 2.1 Hz, 1H), 5.31 – 5.18 (m, 1H), 2.04 (s, 1H), 1.91 – 1.80 (m, 1H), 1.79 – 1.64 (m, 2H), 1.54 – 1.36 (m, 2H), 1.00 – 0.85 (m, 3H).

LCMS (method A, ESI): R_t = 4.518 min, m/z = 373.1 [M + H]$^+$.

(*S*)-3-((1*H*-imidazol-1-yl)methyl)-*N*-(1-(6,7-dihydrothieno[3,2-*c*]pyridin-5(*4H*)-yl)-1-oxopentan-2-yl)benzamide [5a]

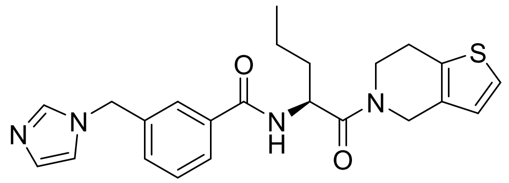

4 (51 mg, 0.137 mmol) and triethylamine (0.0275 mL, 0.199 mmol) were dissolved in DCM (3.5 mL) and the solution was cooled down to 0 °C. Methanesulfonyl chloride (14.31 mL, 0.185 mmol) in DCM (3 mL) was added dropwise over 30 minutes. Then, the reaction mixture was stirred for 1.5 hour at 0 °C and for further 3 hours at room temperature. The mixture was washed with sat. aq. NH_4Cl (5 mL) and brine (5 mL), dried and afterwards concentrated in vacuo.
The crude material was dissolved in DMF (4 mL), imidazole (8.25 mg, 0.279 mmol) and *t*-BuOK (10.4 mg, 0.249 mmol) was added and stirred overnight. The mixture was concentrated in vacuo and purified by flash chromatography followed by RP-flash chromatography affording the title compound as a brownish solid (17 mg, 86 µmol, 46 % yield).

^{1}H NMR (600 MHz, DMSO): δ 8.62 (dd, *J* = 60.5, 8.0 Hz, 1H), 7.80 (dt, *J* = 17.2, 9.7 Hz, 3H), 7.47 – 7.30 (m, 3H), 7.20 (s, 1H), 6.90 (dd, *J* = 10.5, 5.5 Hz, 2H), 5.24 (s, 2H), 4.98 (ddd, *J* = 31.6, 13.9, 8.2 Hz, 1H), 4.63 (ddd, *J* = 159.5, 84.2, 16.4 Hz, 2H), 3.97 – 3.63 (m, 2H), 2.95 – 2.73 (m, 2H), 1.79 – 1.54 (m, 2H), 1.46 – 1.17 (m, 2H), 0.95 – 0,85 (m, 3H).

^{13}C NMR (151 MHz, DMSO): δ 170.64 (s), 165.85 (d, *J* = 8.8 Hz), 137.89 (s), 137.40 (s), 134.32 (d, *J* = 17.5 Hz), 133.20 (s), 132.48 (d, *J* = 7.7 Hz), 132.03 (s), 130.41 (s), 128.63 (d, *J* = 7.6 Hz), 126.79 (d, *J* = 10.0 Hz), 125.38 (s), 125.11 (s), 123.62 (s), 119.54 (s), 49.42 (d, *J* = 55.1 Hz), 45.00 (s), 42.64 (d, *J* = 72.7 Hz), 33.39 (d, *J* = 41.0 Hz), 25.25 (s), 24.20 (s), 18.71 (d, *J* = 6.5 Hz), 13.72 (s).

LCMS (method A, ESI): R_t = 3.324 min, m/z = 423.1 [M + H]$^+$.

HRMS: calculated =423,18492 [M + H]$^+$, found = 423,1856 [M + H]$^+$.

(*S*)-N-(1-(6,7-dihydrothieno[3,2-*c*]pyridin-5(*4H*)-yl)-1-oxopentan-2-yl)-3-((2-imino-3-methyl-2,3-dihydro-1H-imidazol-1-yl)methyl)benzamide [5b]

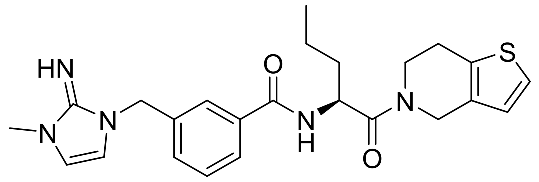

4 (51 mg, 0.137 mmol) and triethylamine (0.0275 mL, 0.199 mmol) were dissolved in DCM (3.5 mL) and the solution was cooled down to 0 °C. Methanesulfonyl chloride (14.31 mL, 0.185 mmol) in DCM (3 mL) was added dropwise over 30 minutes. Then, the reaction mixture was stirred for 1.5 hour at 0 °C and for further 3 hours at room temperature. The mixture was washed with sat. aq. NH_4Cl (5 mL) and brine (5 mL), dried and afterwards concentrated in vacuo.
The crude material was dissolved in DMF (4 mL), 1-methyl-*1H*-imidazol-2-amine hydrochloride (34.7mg, 0.260 mmol), *N,N*-diisopropylethylamine (46.6 mL, 260 µmol) and Tetra-*n*-butylammonium iodide (0.3 mg, 0 mmol) was added. The mixture was stirred for 72 h at 80 °C, subsequently concentrated in vacuo and purified by RP-flash chromatography and preparative reversed phase HPLC obtaining the title compound as a light-yellow solid (7 mg, 15 µmol, 18 % yield).

^{1}H NMR (500 MHz, DMSO): δ 8.62 (d, *J* = 47.5 Hz, 1H), 8.04 – 7.73 (m, 4H), 7.48 (dd, *J* = 14.1, 6.6 Hz, 1H), 7.43 (s, 1H), 7.34 (d, *J* = 4.9 Hz, 1H), 7.08 (d, *J* = 15.9 Hz, 2H), 6.90 (d, *J* = 5.1 Hz, 1H), 5.16 (s, 2H), 5.06 – 4.88 (m, 1H), 4.61 (ddd, *J* = 129.9, 58.1, 17.1 Hz, 2H), 3.88 (dd, *J* = 56.3, 14.7 Hz, 3H), 3.47 (s, 4H), 2.97 – 2.70 (m, 2H), 1.72 (s, *J* = 19.2 Hz, 2H), 1.46 – 1.29 (m, 2H), 0.96 – 0.83 (m, 3H).

^{13}C NMR (126 MHz, DMSO): δ 170.74 (s), 165.83 (s), 158.58 – 157.61 (m), 145.77 (s), 135.37 (s), 134.39 (s), 133.27 – 133.04 (m), 132.52 (d, *J* = 5.6 Hz), 132.16 – 131.66 (m), 130.44 (s), 128.78 (s), 127.09 (s), 125.29 (d, *J* = 36.1 Hz), 123.68 (s), 117.42 (s), 115.57 (s), 49.43 (s), 47.91 (s), 45.14 – 44.88 (m), 42.70 (d, *J* = 65.1 Hz), 32.72 (s), 25.29 (s), 18.80 (s), 13.72 (s).

LCMS (method A, ESI): R_t = 3.153 min, m/z = 452.2 $[M + H]^+$.

HRMS: calculated = 452,21147 $[M + H]^+$, found = 452,2105 $[M + H]^+$.

(*S*)-N-(1-(6,7-dihydrothieno[3,2-*c*]pyridin-5(*4H*)-yl)-1-oxopentan-2-yl)-3-((2-imino-2,3-dihydro-*1H*-imidazol-1-yl)methyl)benzamide [5c]

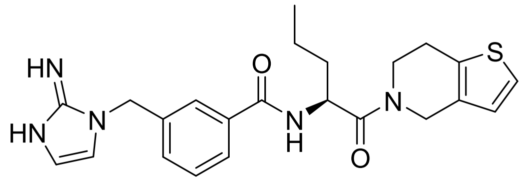

4 (51 mg, 0.137 mmol) and triethylamine (0.0275 mL, 0.199 mmol) were dissolved in DCM (3.5 mL) and the solution was cooled down to 0 °C. Methanesulfonyl chloride (14.31 mL, 0.185 mmol) in DCM (3 mL) was added dropwise over 30 minutes. Then, the reaction mixture was stirred for 1.5 hour at 0 °C and for further 3 hours at room temperature. The mixture was washed with sat. aq. NH_4Cl (5 mL) and brine (5 mL), dried and afterwards concentrated in vacuo.
The crude material was dissolved in DMF (4 mL), *1H*-imidazol-2-amine (5.55 mg, 0,669 mmol), *N,N*-diisopropylethylamine (77.6 mL, 260 µmol) and Tetra-*n*-butylammonium iodide (0,8 mg, 2.22 mmol) was added. The mixture was stirred for 48 h at 70 °C subsequently concentrated in vacuo and purified by RP-flash chromatography and preparative reversed phase HPLC obtaining the title compound as a light brown solid (9 mg, 20 µmol, 9 % yield).

^{1}H NMR (300 MHz, DMSO): δ 8.77 – 8.51 (m, 1H), 7.92 – 7.68 (m, 4H), 7.44 (dd, *J* = 20.8, 7.3 Hz, 2H), 7.34 (d, *J* = 5.1 Hz, 1H), 7.01 (d, *J* = 18.6 Hz, 2H), 6.90 (d, *J* = 5.1 Hz, 1H), 5.15 (s, 2H), 5.00 (d, *J* = 7.2 Hz, 1H), 4.56 (dd, *J* = 91.9, 16.7 Hz, 2H), 3.86 (d, *J* = 30.2 Hz, 2H), 2.83 (d, *J* = 30.8 Hz, 2H), 1.80 – 1.59 (m, 2H), 1.46 – 1.29 (m, 2H), 0.92 – 0.84 (m, 3H).

^{13}C NMR (126 MHz, DMSO): δ 170.81 – 170.68 (m), 165.91 – 165.79 (m), 146.43 – 146.28 (m), 135.58 – 135.45 (m), 134.45 – 134.36 (m), 133.30 – 133.23 (m), 132.55 (s), 130.40 – 130.29 (m), 128.83 (s), 127.05 (s), 125.45 (s), 125.18 – 125.15 (m), 123.70 (s), 116.48 (s), 113.27 (s), 49.41 (s), 47.38 (s), 42.47 (s), 33.54 – 33.44 (m), 25.30 (s), 24.34 – 24.19 (m), 18.81 (s), 13.74 (s).

LCMS (method A, ESI): R_t = 3.156 min, m/z = 438.15 [M + H]$^+$.

HRMS: calculated = 438,19582 [M + H]$^+$, found = 438,1955 [M + H]$^+$.

3-bromo-5-(bromomethyl)benzoic acid [7]

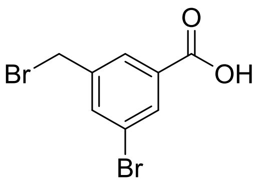

3-bromo-5-methylbenzoic acid (2.0 g, 9.3 mmol), *N*-bromosuccinimide (3.15 g, 17.67 mmol) and benzoyl peroxide (0.11 g, 0.465 mmol) were suspended in MeCN (120 mL) and stirred at reflux for 18 hours. Upon cooling, the mixture was concentrated in vacuo and subsequently purified by RP-flash chromatography. The product was obtained as a white solid (1.63 g, 5.55 mmol, 60 % yield).

^{1}H NMR (300 MHz, DMSO): δ 8.01 (t, *J* = 1.5 Hz, 1H), 7.95 (dt, *J* = 7.0, 1.9 Hz, 2H), 4.78 (s, 2H).

LCMS (method A, ESI): R_t = 4.332 min, *m/z = mass cannot be observed*

3-((*1H*-imidazol-1-yl)methyl)-5-bromobenzoic acid [8a]

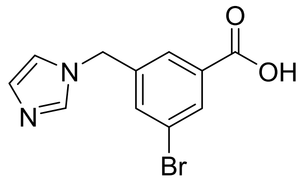

Imidazole (40,3 mg, 0.592 mmol) was dissolved in dioxane (5 mL). Then, a solution of **7** (87 mg, 0.296 mmol) in dioxane (4 mL) was added dropwise. After stirring for 3 hours at 75 °C, the mixture was concentrated in vacuo and purified by RP-flash chromatography. The product was obtained as a white solid. (61 mg, 0.217 mmol, 61 % yield).

^{1}H NMR (400 MHz, DMSO): δ 9.12 (s, 1H), 8.03 (d, *J* = 1.4 Hz, 1H), 7.97 (dt, *J* = 11.7, 1.6 Hz, 2H), 7.71 (d, *J* = 64.1 Hz, 2H), 5.49 (s, 2H).

LCMS (method A, ESI): R_t = 3.002 min, m/z = 282.95 [M + H]$^+$.

(*S*)-3-((*1H*-imidazol-1-yl)methyl)-5-bromo-*N*-(1-(6,7-dihydrothieno[3,2-c]pyridin-5(*4H*)-yl)-1-oxopentan-2-yl)benzamide [10a]

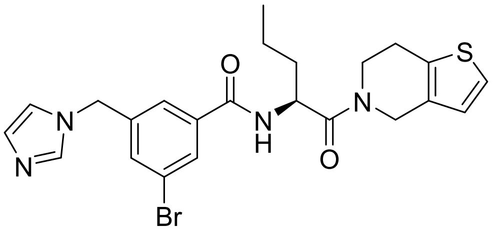

3 (409.5 mg, 1.21 mmol) was dissolved in DCM (10.5 mL) and TFA (4.5 mL). The resulting mixture was stirred for 1 hour at room temperature. Then the mixture was concentrated in vacuo. Without further purification **General Procedure 1** in DMF (16 mL) was followed using **8a** (0.340 g, 1.21 mmol) obtaining the title compound as a light brown solid (392 mg, 0.79 mmol, 65 % yield).

^{1}H NMR (600 MHz, DMSO): δ 9.17 (s, 1H), 8.82 (dd, *J* = 45.6, 7.9 Hz, 1H), 8.19 – 8.04 (m, 1H), 7.92 – 7.76 (m, 3H), 7.67 (d, *J* = 1.5 Hz, 1H), 7.35 (t, *J* = 5.5 Hz, 1H), 6.89 (dd, *J* = 7.5, 5.2 Hz, 1H), 5.46 (d, *J* = 6.0 Hz, 2H), 4.96 (ddd, *J* = 30.5, 13.9, 8.5 Hz, 1H), 4.79 – 4.34 (m, 2H), 3.96 – 3.68 (m, 2H), 2.95 – 2.74 (m, 2H), 1.76 – 1.57 (m, 2H), 1.47 – 1.27 (m, 2H), 0.95 – 0.82 (m, 3H).

LCMS (method A, ESI): R_t = 3.786 min, m/z = 503.10 [M + H]$^+$.

(S)-5-((1H-imidazol-1-yl)methyl)-N-(1-(6,7-dihydrothieno[3,2-c]pyridin-5(4H)-yl)-1-oxopentan-2-yl)-4'-fluoro-2'-methyl-[1,1'-biphenyl]-3-carboxamide [11m]

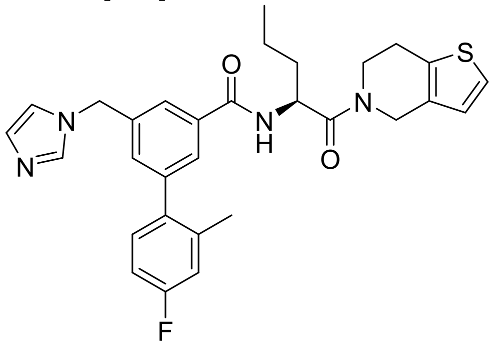

General Procedure 2 was followed using **10a** (32 mg, 64 µmol) and (4-fluoro-2-methylphenyl)boronic acid (19.7 mg, 128 µmol) in 1,4-dioxane/H_2O (4:1) (2 mL) to obtain the title compound (27.0 mg, 50.8 µmol, 80 % yield).

^{1}H NMR (500 MHz, $CDCl_3$): δ 7.82 (d, *J* = 6.9 Hz, 1H), 7.73 (d, *J* = 8.1 Hz, 1H), 7.66 (d, *J* = 5.7 Hz, 1H), 7.29 (dd, *J* = 7.6, 3.2 Hz, 1H), 7.19 – 7.08 (m, 4H), 7.00 – 6.90 (m, 3H), 6.84 – 6.79 (m, 1H), 5.29 – 5.15 (m, 3H), 4.80 – 4.57 (m, 3H), 4.01 – 3.85 (m, 2H), 3.07 – 2.81 (m, 3H), 2.16 (s, 3H), 1.91 – 1.59 (m, 2H), 1.52 – 1.31 (m, 2H), 1.04 – 0.87 (m, 3H).

^{13}C NMR (126 MHz, $CDCl_3$): δ 171.21 (s), 166.14 (d, *J* = 5.4 Hz), 162.48 (d, *J* = 246.8 Hz), 142.69 (s), 137.78 (d, *J* = 8.0 Hz), 137.33 (s), 136.55 (s), 136.13 (s), 135.33 (d, *J* = 9.0 Hz), 134.24 (s), 131.88 (s), 131.34 (d, *J* = 4.1 Hz), 131.24 (d, *J* = 8.5 Hz), 130.73 (s), 127.93 (d, *J* = 5.6 Hz), 125.26 (d, *J* = 15.9 Hz), 124.60 (s), 124.06 (d, *J* = 17.6 Hz), 119.51 (s), 117.19 (d, *J* = 21.1 Hz), 112.99 (d, *J* = 21.1 Hz), 50.94 (d, *J* = 9.9 Hz), 49.86 (d, *J* = 31.3 Hz), 43.51 (d, *J* = 50.8 Hz), 40.82 (s), 35.63 (d, *J* = 35.9 Hz), 25.97 (s), 20.69 (s), 18.74 (s), 14.06 (d, *J* = 6.1 Hz).

LCMS (method B, ESI): R_t = 4.182 min, m/z = 531.10 $[M + H]^+$.

HRMS: calculated = 531,22245 $[M + H]^+$, found = 531,2241 $[M + H]^+$.

(*S*)-3-((*1H*-imidazol-1-yl)methyl)-*N*-(1-(6,7-dihydrothieno[3,2-*c*]pyridin-5(*4H*)-yl)-1-oxopentan-2-yl)-5-(6-fluoro-2-methylpyridin-3-yl)benzamide 11n

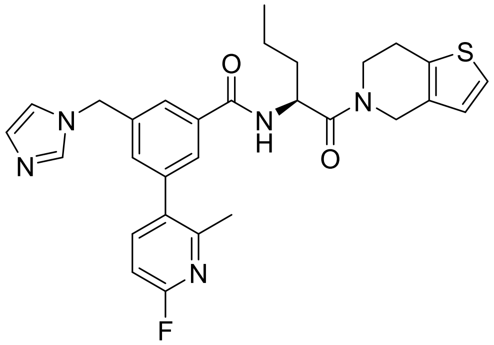

General Procedure 2 was followed using **10a** (50 mg, 99.7 µmol) and (6-fluoro-2-methylpyridin-3-yl)boronic acid (30.9 mg, 199 µmol)) in 1,4-dioxane/H_2O (4:1) (2 mL) to obtain the title compound (19 mg, 35.7 µmol, 36 % yield).

^{1}H NMR (600 MHz, DMSO): δ 8.85 – 8.58 (m, 1H), 7.82 (dd, *J* = 32.1, 15.4 Hz, 5H), 7.46 (d, *J* = 7.9 Hz, 1H), 7.32 (dd, *J* = 14.9, 4.5 Hz, 1H), 7.25 (s, 2H), 7.11 (d, *J* = 7.2 Hz, 1H), 6.96 – 6.85 (m, 2H), 5.30 (s, 2H), 5.01 (dd, *J* = 33.1, 6.3 Hz, 1H), 4.79 – 4.38 (m, 2H), 3.97 – 3.69 (m, 2H), 2.84 (dd, *J* = 46.8, 30.6 Hz, 2H), 2.33 (s, 3H), 1.72 – 1.63 (m, 2H), 1.44 – 1.28 (m, 2H), 0.94 – 0.84 (m, 3H).

^{13}C NMR (151 MHz, DMSO): δ 170.58 (s), 165.48 (d, *J* = 10.8 Hz), 162.25 (s), 160.69 (s), 154.12 (d, *J* = 14.7 Hz), 143.23 (d, *J* = 7.8 Hz), 138.55 – 138.24 (m), 137.47 (s), 134.67 (d, *J* = 19.0 Hz), 133.29 (d, *J* = 27.7 Hz), 132.47 (d, *J* = 9.4 Hz), 132.02 (s), 131.10 (s), 128.85 (s), 127.23 (s), 126.33 (d, *J* = 11.3 Hz), 125.37 (s), 125.07 (s), 123.62 (s), 119.53 (s), 106.63 (d, *J* = 37.7 Hz), 49.16 (d, *J* = 26.5 Hz), 45.02 (s), 42.66 (d, *J* = 76.9 Hz), 33.44 (d, *J* = 39.0 Hz), 25.27 (s), 24.19 (s), 22.53 (s), 18.73 (s), 13.71 (s).

LCMS (method B, ESI): R_t = 4.692 min, m/z = 532.20 $[M + H]^+$.

HRMS: calculated = 532,21770 $[M + H]^+$, found = 532,2187 $[M + H]^+$.

(*S*)-3-((*1H*-imidazol-1-yl)methyl)-*N*-(1-(6,7-dihydrothieno[3,2-*c*]pyridin-5(*4H*)-yl)-1-oxopentan-2-yl)-5-(1-ethyl-3-(trifluoromethyl)-*1H*-pyrazol-4-yl)benzamide [11o] (LH168)

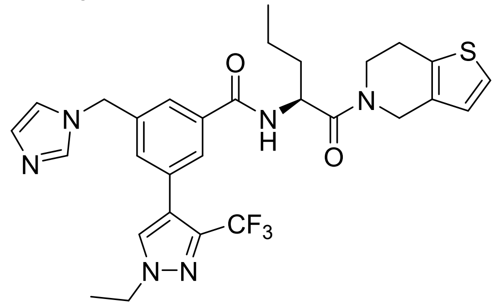

General procedure 2 was followed using **10a** (50 mg, 99.7 µmol) and 1-ethyl-3-(trifluoromethyl)-1H-pyrazol-4-yl)boronic acid (41.5 mg, 199 µmol) in 1,4-dioxane/H_2O (4:1) (2 mL) to obtain the title compound (28 mg, 47.9 µmol, 48 % yield).

^{1}H NMR (600 MHz, DMSO): δ 8.82 – 8.59 (m, 1H), 8.22 (s, 1H), 7.91 – 7.70 (m, 3H), 7.34 (dd, *J* = 17.5, 12.4 Hz, 2H), 7.20 (s, 1H), 6.95 – 6.84 (m, 2H), 5.28 (s, 2H), 5.08 – 4.91 (m, 1H), 4.78 – 4.36 (m, 2H), 4.25 (q, *J* = 7.2 Hz, 2H), 3.98 – 3.72 (m, 2H), 2.94 – 2.70 (m, 2H), 1.77 – 1.61 (m, 2H), 1.48 – 1.27 (m, 5H), 0.96 – 0.84 (m, 3H).

^{13}C NMR (151 MHz, DMSO): δ 170.62 (d, *J* = 8.6 Hz), 165.60 (d, *J* = 6.5 Hz), 138.40 (d, *J* = 9.1 Hz), 137.44 (s), 136.33 (d, *J* = 35.9 Hz), 134.81 (d, *J* = 15.1 Hz), 133.23 (s), 132.49 (d, *J* = 12.8 Hz), 131.51 (s), 131.01 (s), 129.97 (s), 128.84 (s), 126.81 (s), 125.39 (s), 125.11 (s), 123.65 (s), 121.71 (d, *J* = 269.0 Hz), 119.95 (s), 119.48 (s), 49.31 (s), 49.08 (s), 47.26 (s), 42.92 (s), 42.43 (s), 33.60 (s), 25.28 (s), 18.71 (d, *J* = 6.3 Hz), 15.07 (s), 13.73 (s).

LCMS (method A, ESI): R_t = 3.621 min, m/z = 585.30 $[M + H]^+$.

HRMS: calculated = 585,22541 $[M + H]^+$, found = 585,2265 $[M + H]^+$.

(*S*)-3-((*1H*-imidazol-1-yl)methyl)-*N*-(1-(6,7-dihydrothieno[3,2-*c*]pyridin-5(*4H)*-yl)-1-oxopentan-2-yl)-5-(*1H*-indazol-4-yl)benzamide 11p

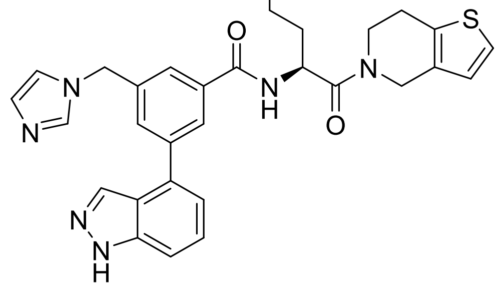

General procedure 2 was followed using **10a** (60 mg, 119.6 µmol) and (*1H*-indazol-4-yl)boronic acid (38.7 mg, 239.3 µmol) in 1,4-dioxane/H_2O (4:1) (2.4 mL) to obtain the title compound (31 mg, 57.5 µmol, 48 % yield).

^{1}H NMR (500 MHz, DMSO): δ 8.84 (dd, *J* = 45.9, 8.0 Hz, 1H), 8.11 (t, *J* = 17.4 Hz, 2H), 7.87 – 7.70 (m, 3H), 7.58 (d, *J* = 8.3 Hz, 1H), 7.46 (t, *J* = 7.7 Hz, 1H), 7.37 – 7.26 (m, 3H), 6.90 (m, 2H), 5.37 (s, 2H), 5.08 – 4.95 (m, 1H), 4.64 (ddd, *J* = 132.8, 70.8, 16.4 Hz, 2H), 3.98 – 3.70 (m, 2H), 2.86 (dd, *J* = 49.4, 31.0 Hz, 2H), 1.77 – 1.66 (m, 2H), 1.43 – 1.33 (m, 2H), 0.88 (ddd, *J* = 25.7, 16.5, 8.9 Hz, 3H).

δ 171.07 (s), 166.31 (d, *J* = 10.7 Hz), 140.92 (s), 140.22 (s), 139.42 (d, *J* = 9.4 Hz), 138.07 (s), 135.66 (d, *J* = 15.8 Hz), 133.67 (s), 133.23 (s), 132.97 (d, *J* = 6.5 Hz), 132.53 (s), 130.28 (d, *J* = 11.6 Hz), 129.34 (s), 126.72 (s), 126.62 (d, *J* = 7.3 Hz), 125.87 (s), 125.57 (s), 124.12 (s), 121.30 (s), 120.28 (s, *J* = 17.1 Hz), 120.15 (s), 110.34 (s), 49.80 (s), 49.62 (s), 43.39 (s), 42.91 (s), 33.91 (d, *J* = 35.2 Hz), 25.77 (s), 19.25 (s), 14.24 (s).

LCMS (method B, ESI): R_t = 4.458 min, m/z = 539.20 $[M + H]^+$.

HRMS: calculated = 539,22237 $[M + H]^+$, found = 539,2243 $[M + H]^+$.

3-bromo-5-((2-imino-3-methyl-2,3-dihydro-1H-imidazol-1-yl)methyl)benzoic acid [8b]

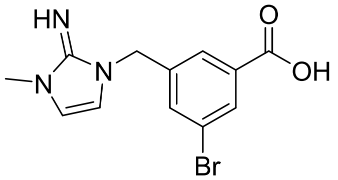

7 (36.7 mg 0.125 mmol), 1-methyl-*1H*-imidazol-2-amine hydrochloride (20 mg, 0.15 mmol), tetra-*n*-butylammonium iodide (0,8 mg, 0.002 mmol) and *N,N*-diisopropylethylamine (54.33 mL, 0.312 µmol) were suspended in 2.5 mL MeCN and stirred at 60 °C for 18 h. Upon cooling, the mixture was concentrated in vacuo, Purification by RP-flash chromatography afforded a light brown solid (15.5 mg, 0.05 mmol, 40 % yield).

^{1}H NMR (400 MHz, DMSO): δ 8.04 – 7.96 (m,3), 7.85 (d, *J* = 24.1 Hz, 2H), 7.14 (dd, *J* = 41.0, 2.4 Hz, 2H), 5.18 (s, 2H), 3.47 (s, 3H).

LCMS (method A, ESI): R_t = 3.086 min, m/z = 312.0 $[M + H]^+$.

(*S*)-3-bromo-*N*-(1-(6,7-dihydrothieno[3,2-*c*]pyridin-5(*4H*)-yl)-1-oxopentan-2-yl)-5-((2-imino-3-methyl-2,3-dihydro-*1H*-imidazol-1-yl)methyl)benzamide [10b]

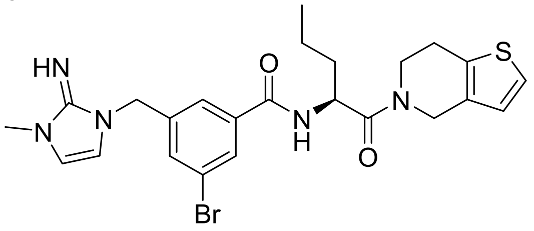

3 (114.6 mg, 0.339 mmol) were dissolved in DCM (4 mL) and TFA (1.7 mL) were added to the solution. After stirring for 1 hour at room temperature the mixture was concentrated in vacuo. Without further purification, **general procedure 1** was followed using **8b** (105 mg, 0.339 mmol) in DMF (10 mL) obtaining the title compound as a light brown solid (84 mg, 0,79 mmol, 65 % yield).

^{1}H NMR (400 MHz, DMSO): δ 8.80 (dd, *J* = 30.1, 7.9 Hz, 1H), 8.11 (d, *J* = 27.7 Hz, 1H), 7.92 (s, 2H), 7.81 – 7.67 (m, 2H), 7.35 (d, *J* = 5.0 Hz, 1H), 7.15 (d, *J* = 2.3 Hz, 1H), 7.08 (s, 1H), 6.90 (d, *J* = 5.0 Hz, 1H), 5.13 (s, 2H), 5.02 – 4.88 (m, *J* = 6.2 Hz, 1H), 4.63 (m,4.76 – 4.38, 2H), 3.99 – 3.68 (m, 2H), 3.46 (s, 3H), 2.91 – 2.74 (m, 2H), 1.74 – 1.59 (m, 2H), 1.46 – 1.30 (m, 2H), 0.94 – 0.82 (m, 3H).

LCMS (method A, ESI): R_t = 3.734 min, m/z = 532.1 $[M + H]^+$.

(*S*)-*N*-(1-(6,7-dihydrothieno[3,2-*c*]pyridin-5(*4H*)-yl)-1-oxopentan-2-yl)-3-((2-imino-3-methyl-2,3-dihydro-*1H*-imidazol-1-yl)methyl)-5-(pyridin-4-ylamino)benzamide [11a]

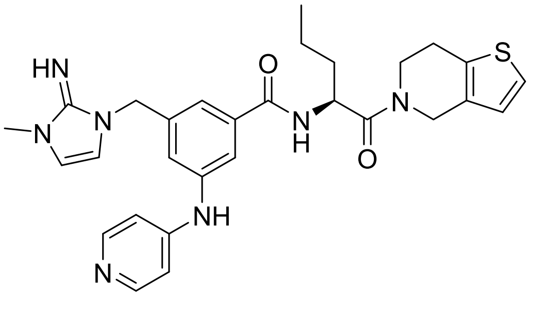

General procedure 3 was followed using **10b** (25 mg, 47.1 µmol) and pyridin-4-amine (4.7 mg, 49.5 µmol) in 1,4-dioxane (1.5 mL) to obtain the title compound (6 mg, 11 µmol, 23 % yield).

^{1}H NMR (600 MHz, DMSO): δ 9.05 (s, 1H), 8.62 (dd, *J* = 64.0, 7.9 Hz, 1H), 8.23 (s, 2H), 7.91 (d, *J* = 15.1 Hz, 2H), 7.66 – 7.57 (m, 1H), 7.45 (d, *J* = 19.2 Hz, 1H), 7.35 (d, *J* = 5.0 Hz, 1H), 7.22 (s, 1H), 7.15 (d, *J* = 2.2 Hz, 1H), 7.09 (s, 1H), 6.97 – 6.86 (m, 3H), 5.13 (s, 2H), 4.98 (dt, *J* = 20.5, 6.3 Hz, 1H), 4.78 – 4.36 (m, 2H), 3.98 – 3.70 (m, 2H), 3.47 (s, 3H), 3.03 – 2.73 (m, 2H), 1.76 – 1.62 (m, 2H), 1.46 – 1.28 (m, 2H), 1.00 – 0.73 (m, 3H).

^{13}C NMR (151 MHz, DMSO): δ 170.62 (s), 165.70 (s), 149.95 (s), 149.79 – 149.66 (m), 145.75 (s), 141.00 (s), 136.75 – 136.35 (m), 135.88 (s), 133.32 – 133.09 (m), 132.50 (d, *J* = 5.4 Hz), 132.09 – 131.86 (m), 125.44 (s),

125.28 – 125.02 (m), 123.71 (s), 120.97 – 120.90 (m), 120.82 – 120.75 (m), 118.44 (s), 117.43 (s), 115.70 (s), 109.57 (s), 49.39 (s), 47.89 (s), 42.93 (s), 42.44 (s), 33.49 (s), 32.74 (s), 25.28 (s), 18.76 (s), 13.75 (s).

LCMS (method C, ESI): R_t = 5.816 min, m/z = = 544.25 $[M + H]^+$.

HRMS: calculated = 544,24892 $[M + H]^+$, found = 544,2500 $[M + H]^+$.

(*S*)-*N*-(1-(6,7-dihydrothieno[3,2-*c*]pyridin-5(*4H*)-yl)-1-oxopentan-2-yl)-3-((2-imino-3-methyl-2,3-dihydro-*1H*-imidazol-1-yl)methyl)-5-(pyridin-3-ylamino)benzamide [11b]

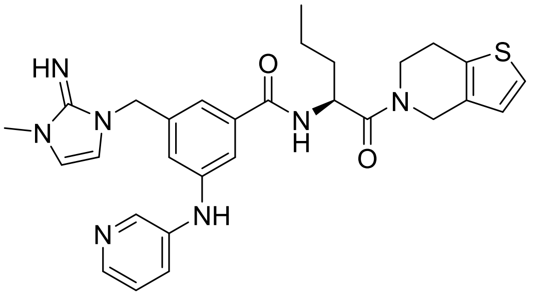

General procedure 3 was followed using **10b** (32 mg, 60.3 µmol) and pyridin-4-amine (6.8 mg, 72.4 µmol) in 1,4-dioxane (1.5 mL) to obtain the title compound (4 mg, 7 µmol, 12 % yield).

^{1}H NMR (600 MHz, DMSO): δ 8.65 – 8.49 (m, 2H), 8.39 (d, *J* = 2.4 Hz, 1H), 8.10 (d, *J* = 4.0 Hz, 1H), 7.92 (s, 2H), 7.48 (dd, *J* = 17.9, 9.6 Hz, 2H), 7.36 (d, *J* = 5.1 Hz, 1H), 7.30 – 7.25 (m, 2H), 7.13 – 7.07 (m, 3H), 6.90 (t, *J* = 6.0 Hz, 1H), 5.09 (s, 2H), 5.01 – 4.92 (m, 1H), 4.60 (ddd, *J* = 148.9, 47.6, 14.2 Hz, 2H), 3.99 – 3.71 (m, 2H), 3.47 (s, 3H), 2.96 – 2.74 (m, 2H), 1.74 – 1.61 (m, 2H), 1.44 – 1.30 (m, 2H), 0.93 – 0.87 (m, 3H).

LCMS (method A, ESI): R_t = 3.379 min, m/z = 544.25 $[M + H]^+$.

HRMS: calculated = 544,24892 $[M + H]^+$, found = 544,2481 $[M + H]^+$.

(*S*)-*N*-(1-(6,7-dihydrothieno[3,2-*c*]pyridin-5(*4H*)-yl)-1-oxopentan-2-yl)-4'-fluoro-5-((2-imino-3-methyl-2,3-dihydro-*1H*-imidazol-1-yl)methyl)-2'-methyl-[1,1'-biphenyl]-3-carboxamide [11c]

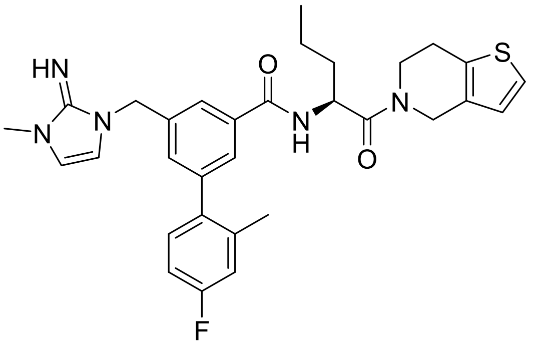

General procedure 2 was followed using **10b** (33 mg, 62.2 µmol) and (4-fluoro-2-methylphenyl)boronic acid (19.1 mg, 124.4 µmol) in 1,4-dioxane/H_2O (4:1) (3 mL) to obtain the title compound (11 mg, 19.7 µmol, 31 % yield).

^{1}H NMR (600 MHz, DMSO): δ 8.72 (dd, *J* = 50.5, 7.9 Hz, 1H), 7.94 – 7.78 (m, 4H), 7.44 (d, *J* = 8.1 Hz, 1H), 7.34 (t, *J* = 5.8 Hz, 1H), 7.29 (dt, *J* = 10.7, 5.4 Hz, 1H), 7.21 (dd, *J* = 10.1, 2.7 Hz, 1H), 7.17 (d, *J* = 2.3 Hz, 1H), 7.14 (t, *J* = 8.5 Hz, 1H), 7.07 (d, *J* = 2.2 Hz, 1H), 6.89 (t, *J* = 4.9 Hz, 1H), 5.18 (s, 2H), 5.04 – 4.95 (m, 1H), 4.78 – 4.36 (m, 2H), 3.98 – 3.70 (m, 2H), 3.46 (d, *J* = 9.3 Hz, 3H), 2.96 – 2.73 (m, 2H), 2.21 (s, 3H), 1.74 – 1.61 (m, 2H), 1.45 – 1.25 (m, 2H), 0.94 – 0.83 (m, 3H).

^{13}C NMR (151 MHz, DMSO): δ 165.52 – 165.47 (m), 162.41 – 162.31 (m), 160.79 – 160.69 (m), 145.67 (s), 140.62 (s), 137.80 (s), 136.53 – 136.34 (m), 135.41 – 135.14 (m), 134.65 – 134.48 (m), 133.25 – 133.12 (m), 132.52 – 132.39 (m), 131.47 (s), 131.23 – 131.06 (m), 127.51 (s), 126.04 (s), 125.40 (s), 123.65 (s), 117.44 (s), 116.97 – 116.60 (m), 115.52 (s), 112.73 (d, *J* = 21.1 Hz), 48.56 (s), 47.82 (s), 42.91 (s), 42.38 (s), 33.43 (s), 32.69 (s), 25.25 (s), 20.13 (s), 18.74 (s), 13.69 (s).

LCMS (method A, ESI): R_t =3.958 min, m/z = 560.25 $[M + H]^+$.

HRMS: calculated = 560,24900 [M + H]$^+$, found = 560,2505 [M + H]$^+$.

(*S*)-*N*-(1-(6,7-dihydrothieno[3,2-*c*]pyridin-5(*4H*)-yl)-1-oxopentan-2-yl)-3-((2-imino-3-methyl-2,3-dihydro-*1H*-imidazol-1-yl)methyl)-5-(5-methoxypyridin-3-yl)benzamide [11d]

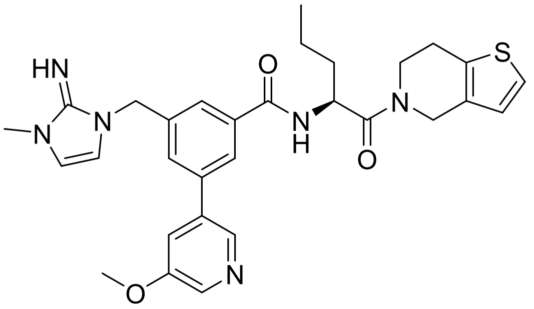

General procedure 2 was followed using **10b** (40 mg, 75.4 µmol) and (5-methoxypyridin-3-yl)boronic acid) (23.1 mg, 150.7 µmol) in 1,4-dioxane/H_2O (4:1) (3.75 mL) to obtain the title compound (22 mg, 39.4 µmol, 52 % yield).

^{1}H NMR (600 MHz, DMSO): δ 8.83 (dd, *J* = 47.2, 8.0 Hz, 1H), 8.61 – 8.59 (m, 1H), 8.38 – 8.36 (m, 1H), 8.27 – 8.17 (m, 1H), 7.93 – 7.91 (m, 3H), 7.88 – 7.79 (m, 1H), 7.71 (d, *J* = 2.2 Hz, 1H), 7.35 – 7.32 (m, 1H), 7.20 (d, *J* = 2.3 Hz, 1H), 7.07 (d, *J* = 2.3 Hz, 1H), 6.93 – 6.86 (m, 1H), 5.20 (s, 2H), 5.02 (ddd, *J* = 29.3, 14.5, 7.9 Hz, 1H), 4.80 – 4.38 (m, 2H), 4.00 – 3.70 (m, 5H), 3.46 (s, 3H), 2.86 (dq, *J* = 63.1, 17.0 Hz, 2H), 1.80 – 1.61 (m, 2H), 1.51 – 1.22 (m, 2H), 0.95 – 0.83 (m, 3H).

^{13}C NMR (151 MHz, DMSO): δ 170.69 (s), 165.43 (d, *J* = 6.6 Hz), 155.63 (s), 145.68 (s), 140.21 (s), 137.38 (s), 136.48 (s), 136.29 (d, *J* = 6.9 Hz), 135.51 – 134.87 (m), 133.23 (s), 132.51 (d, *J* = 7.2 Hz), 132.02 (s), 129.54 (d, *J* = 14.3 Hz), 127.16 (s), 125.47 (d, *J* = 15.4 Hz), 125.09 (s), 123.67 (s), 119.13 (s), 117.48 (s), 115.58 (s), 55.83 (s), 49.55 (d, *J* = 49.0 Hz), 47.92 (s), 42.98 (s), 42.44 (s), 33.46 (d, *J* = 34.3 Hz), 32.73 (s), 25.29 (s), 18.83 (t, *J* = 17.1 Hz), 13.67 (d, *J* = 18.0 Hz).

LCMS (method A, ESI): R_t = 3.544 min, m/z = 559.25 [M + H]$^+$.

HRMS: calculated = 559,24859 [M + H]$^+$, found = 559,2503 [M + H]$^+$.

(*S*)-*N*-(1-(6,7-dihydrothieno[3,2-*c*]pyridin-5(*4H*)-yl)-1-oxopentan-2-yl)-3-(1-ethyl-3-(trifluoromethyl)-*1H*-pyrazol-4-yl)-5-((2-imino-3-methyl-2,3-dihydro-*1H*-imidazol-1-yl)methyl)benzamide [11e]

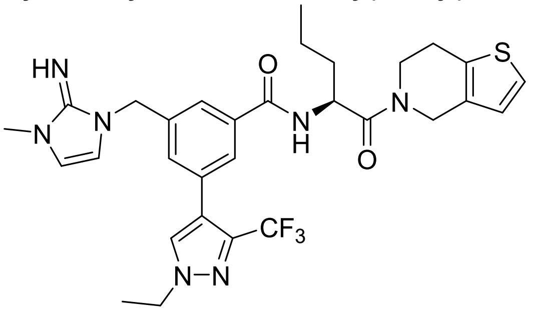

General procedure 2 was followed using **10b** (40 mg, 75.4 µmol) and (1-ethyl-3-(trifluoromethyl)-1H-pyrazol-4-yl)boronic acid (31.4 mg, 150.8 µmol) in 1,4-dioxane/H_2O (4:1) (3.75 mL) to obtain the title compound (23 mg, 37.5 µmol, 50 % yield).

^{1}H NMR (600 MHz, DMSO): δ 8.76 – 8.59 (m, 1H), 8.24 (d, *J* = 3.7 Hz, 1H), 8.03 – 7.77 (m, 4H), 7.36 – 7.29 (m, 2H), 7.10 (dd, *J* = 11.9, 2.3 Hz, 2H), 6.90 (t, *J* = 5.0 Hz, 1H), 5.20 (s, 2H), 5.06 – 4.94 (m, 1H), 4.79 – 4.38 (m, 2H), 4.26 (q, *J* = 7.3 Hz, 2H), 3.98 – 3.70 (m, 2H), 3.47 (s, 3H), 1.76 – 1.62 (m, 2H), 1.48 – 1.31 (m, 5H), 0.95 – 0.81 (m, 3H).

^{13}C NMR (151 MHz, DMSO): δ 170.64 (s), 165.54 – 165.32 (m), 145.82 (s), 137.77 (s), 136.20 (s), 135.85 (s), 134.88 (s), 132.51 (d, *J* = 10.7 Hz), 131.56 (s), 131.09 (s), 129.41 (s), 126.98 (s), 126.17 (s), 125.42 (s), 123.69 (s), 122.62 (s), 119.81 (s), 118.05 (s), 117.44 (s), 115.63 (s), 49.55 (d, *J* = 55.1 Hz), 47.75 (s), 47.31 (s), 42.96 (s), 42.43 (s), 33.43 (d, *J* = 38.4 Hz), 32.69 (s), 18.74 (s), 15.15 (d, *J* = 11.6 Hz), 13.72 (s).

LCMS (method A, ESI): R_t = 3.809 min, m/z = 614.25 [M + H]$^+$.

HRMS: calculated = 614,25196 [M + H]$^+$, found = 614,2527 [M + H]$^+$.

(*S*)-*N*-(1-(6,7-dihydrothieno[3,2-*c*]pyridin-5(*4H*)-yl)-1-oxopentan-2-yl)-3-((2-imino-3-methyl-2,3-dihydro-*1H*-imidazol-1-yl)methyl)-5-(1-methyl-3-(trifluoromethyl)-*1H*-pyrazol-4-yl)benzamide [11f]

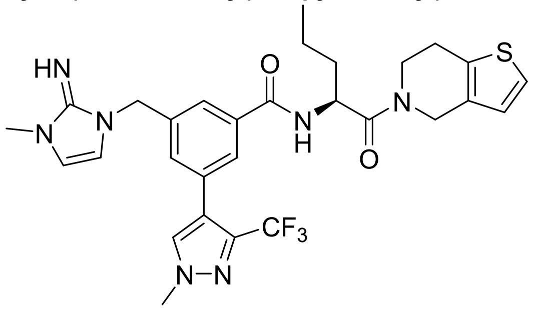

General procedure 2 was followed using **10b** (40 mg, 75.4 µmol) and 1-methyl-4-(4,4,5,5-tetramethyl-1,3,2-dioxaborolan-2-yl)-3-(trifluoromethyl)-1H-pyrazole (41.6 mg, 150.8 µmol) in 1,4-dioxane/H_2O (4:1) (3.75 mL) to obtain the title compound (24 mg, 40.2 µmol, 53 % yield).

^{1}H NMR (600 MHz, DMSO): δ 8.77 – 8.62 (m, 1H), 8.21 – 8.12 (m, 1H), 7.96 – 7.76 (m, 4H), 7.36 – 7.27 (m, 2H), 7.11 (dd, *J* = 12.6, 2.3 Hz, 2H), 6.90 (d, *J* = 5.2 Hz, 1H), 5.21 (s, 2H), 5.01 (ddd, *J* = 29.9, 14.2, 7.9 Hz, 1H), 4.79 – 4.38 (m, 2H), 4.01 – 3.72 (m, 5H), 3.47 (s, 3H), 2.96 – 2.74 (m, 2H), 1.76 – 1.62 (m, 2H), 1.47 – 1.31 (m, 2H), 0.94 – 0.84 (m, 3H).

^{13}C NMR (151 MHz, DMSO): δ 170.62 (s), 165.47 (s), 145.83 (s), 136.35 (d, *J* = 35.9 Hz), 135.89 (s), 134.87 (s), 132.93 (s), 132.50 (d, *J* = 10.3 Hz), 132.01 (s), 130.98 (s), 129.28 (s), 126.93 (s), 126.15 (s), 125.41 (s), 123.70 (d, *J* = 7.3 Hz), 122.58 (s), 119.95 (s), 117.43 (s), 115.63 (s), 49.36 (s), 47.75 (s), 45.05 (s), 42.95 (s), 42.43 (s), 33.44 (d, *J* = 38.3 Hz), 32.68 (s), 25.28 (s), 18.71 (d, *J* = 7.2 Hz), 13.65 (d, *J* = 18.3 Hz).

LCMS (method A, ESI): R_t = 3.726 min, m/z = 600.25 $[M + H]^+$.

HRMS: calculated = 600,23631 $[M + H]^+$, found = 600,2362 $[M + H]^+$.

(*S*)-*N*-(1-(6,7-dihydrothieno[3,2-*c*]pyridin-5(*4H*)-yl)-1-oxopentan-2-yl)-3-((2-imino-3-methyl-2,3-dihydro-*1H*-imidazol-1-yl)methyl)-5-(*1H*-indazol-4-yl)benzamide [11g]

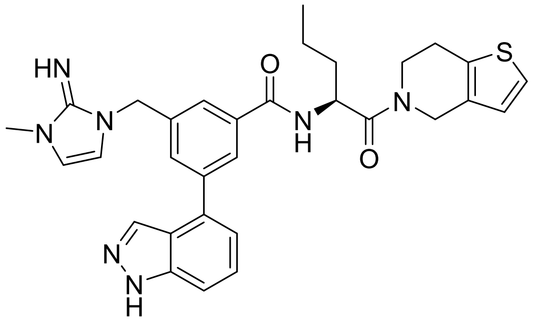

General procedure 2 was followed using **10b** (40 mg, 75.4 µmol) and (*1H*-indazol-4-yl)boronic acid (24.4 mg, 150.8 µmol) in 1,4-dioxane/H_2O (4:1) (3.75 mL) to obtain the title compound (20 mg, 35.2 µmol, 47 % yield).

^{1}H NMR (600 MHz, DMSO): δ 8.86 (dd, *J* = 51.6, 7.9 Hz, 1H), 8.24 – 8.13 (m, 2H), 7.98 (s, 2H), 7.86 (dd, *J* = 16.8, 7.0 Hz, 2H), 7.61 (d, *J* = 8.4 Hz, 1H), 7.49 (t, *J* = 7.7 Hz, 1H), 7.37 – 7.31 (m, 2H), 7.27 (d, *J* = 2.3 Hz, 1H), 7.09 (d, *J* = 2.1 Hz, 1H), 6.94 – 6.85 (m, 1H), 5.27 (s, 2H), 5.02 (ddd, *J* = 30.2, 13.9, 8.0 Hz, 1H), 4.81 – 4.38 (m, 2H), 4.01 – 3.69 (m, 2H), 3.47 (s, 3H), 2.98 – 2.73 (m, 2H), 1.80 – 1.57 (m, 2H), 1.51 – 1.20 (m, 3H), 0.97 – 0.85 (m, 3H).

^{13}C NMR (151 MHz, DMSO): δ 170.69 (s), 165.75 (s), 145.75 (s), 140.51 (s), 139.90 (s), 136.14 (s), 135.37 (d, *J* = 15.5 Hz), 133.24 (s), 132.62 (s), 132.52 (d, *J* = 5.6 Hz), 132.04 (s), 130.18 (s), 126.51 (s), 126.31 (s), 125.43 (s), 125.10 (s), 123.68 (s), 120.85 (s), 119.96 (s), 117.48 (s), 115.75 (s), 110.04 (s), 49.43 (s), 47.95 (s), 42.96 (s), 42.44 (s), 33.40 (d, *J* = 38.6 Hz), 32.74 (s), 25.30 (s), 18.79 (s), 13.67 (d, *J* = 20.0 Hz).

LCMS (method A, ESI): R_t = 3.711 min, m/z = 568.25 $[M + H]^+$.

HRMS: calculated = 568,24892 $[M + H]^+$, found = 568,2505 $[M + H]^+$.

(*S*)-*N*³-(1-(6,7-dihydrothieno[3,2-*c*]pyridin-5(*4H*)-yl)-1-oxopentan-2-yl)-5-((2-imino-3-methyl-2,3-dihydro-*1H*-imidazol-1-yl)methyl)-[1,1'-biphenyl]-3,3'-dicarboxamide [11i]

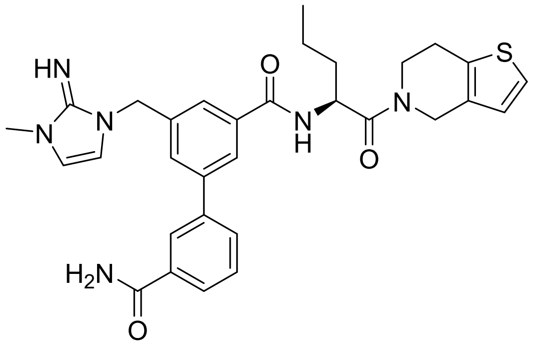

General procedure 2 was followed using **10b** (45 mg, 84.8 μmol) and (3-carbamoylphenyl)boronic acid (28.0 mg, 169.7 μmol) in 1,4-dioxane/H_2O (4:1) (3.75 mL) to obtain the title compound (29 mg, 50.8 μmol, 60 % yield).

^{1}H NMR (600 MHz, DMSO): δ 8.86 (dd, *J* = 49.9, 7.8 Hz, 1H), 8.26 – 8.08 (m, 3H), 8.00 – 7.76 (m, 5H), 7.60 (t, *J* = 7.7 Hz, 1H), 7.49 (s, 1H), 7.34 (t, *J* = 6.0 Hz, 1H), 7.20 (d, *J* = 2.4 Hz, 1H), 7.08 (d, *J* = 2.2 Hz, 1H), 6.90 (d, *J* = 5.0 Hz, 1H), 5.21 (s, 2H), 5.02 (ddd, *J* = 29.6, 14.1, 7.8 Hz, 1H), 4.80 – 4.39 (m, 2H), 4.00 – 3.71 (m, 2H), 3.46 (s, 3H), 2.98 – 2.74 (m, 2H), 1.80 – 1.67 (m, 2H), 1.52 – 1.31 (m, 2H), 0.93 – 0.85 (m, 3H).

^{13}C NMR (151 MHz, DMSO): δ 170.67 (s), 167.74 (s), 165.58 (s), 145.67 (s), 140.20 (s), 139.11 (s), 136.10 (s), 135.15 (s), 133.22 (s), 132.52 (s, *J* = 5.9 Hz), 132.48 (s), 132.14 – 131.84 (m), 129.74 (s), 129.41 (s), 128.93 (s), 126.93 (s), 126.62 (s), 126.15 (s), 125.41 (s), 123.66 (s), 117.45 (s), 115.58 (s), 49.36 (s), 47.96 (s), 42.96 (s), 42.42 (s), 33.45 (d, *J* = 36.0 Hz), 32.72 (s), 25.28 (s), 18.78 (s), 13.73 (s).

LCMS (method A, ESI): R_t = 3.620 min, m/z = 571.25 $[M + H]^+$.

HRMS: calculated = 571,24859 $[M + H]^+$, found = 571,2487 $[M + H]^+$.

(*S*)-*N*-(1-(6,7-dihydrothieno[3,2-*c*]pyridin-5(*4H*)-yl)-1-oxopentan-2-yl)-3-((6-hydroxypyridin-3-yl)amino)-5-((2-imino-3-methyl-2,3-dihydro-*1H*-imidazol-1-yl)methyl)benzamide [11h]

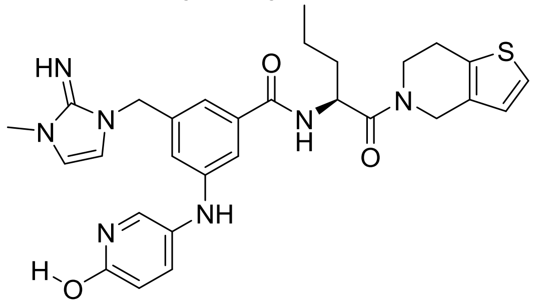

General procedure 3 was followed using **10b** (40 mg, 74.4 μmol) and 5-aminopyridin-2(*5H*)-one (10.0 mg, 90.5 μmol) in 1,4-dioxane (2.5 mL) to obtain the title compound (2 mg, 3.6 μmol, 5 % yield).

LCMS (method A, ESI): R_t = 3.074 min, m/z = 560.20 $[M + H]^+$.

HRMS: calculated = 560,24384 $[M + H]^+$, found = 560,245 $[M + H]^+$.

(*S*)-*N*-(1-(6,7-dihydrothieno[3,2-*c*]pyridin-5(*4H*)-yl)-1-oxopentan-2-yl)-3-((2-imino-3-methyl-2,3-dihydro-*1H*-imidazol-1-yl)methyl)-5-(piperidin-4-ylamino)benzamide [11j]

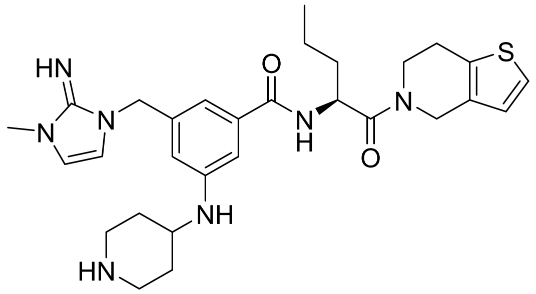

General procedure 3 was followed using **10b** (40 mg, 74.4 µmol) and *tert*-butyl 4-aminopiperidine-1-carboxylate (18.1 mg, 72.4 µmol) in 1,4-dioxane (1.5 mL) to obtain a brownish solid. It was suspended in 1,4-dioxane (0.6 mL) and HCl in 1,4-dioxane (1 mL, 4 M) was added and stirred for 2 h. All volatiles were removed under reduced pressure and the title compound could be obtained
(2 mg, 3.4 µmol, 3 % yield).

LCMS (method A, ESI): R_t = 3.376 min, m/z = 550.30 $[M + H]^+$.

HRMS: calculated = 550,29587 $[M + H]^+$, found = 550,2964 $[M + H]^+$.

(*S*)-3-((6-azaspiro[3.4]octan-2-yl)amino)-*N*-(1-(6,7-dihydrothieno[3,2-*c*]pyridin-5(*4H*)-yl)-1-oxopentan-2-yl)-5-((2-imino-3-methyl-2,3-dihydro-*1H*-imidazol-1-yl)methyl)benzamide [11k]

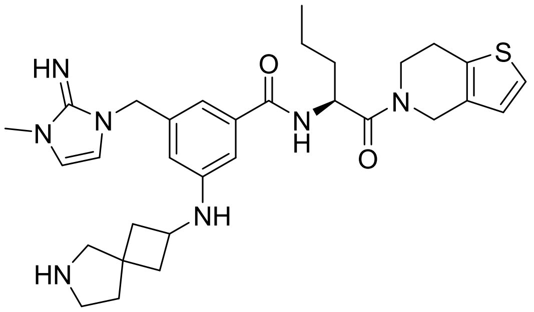

General procedure 3 was followed using **10b** (45 mg, 84.8 µmol) and *tert*-butyl 2-amino-6-azaspiro[3.4]octane-6-carboxylate (23.1 mg, 72.4 µmol) in 1,4-dioxane (1.5 mL) to obtain a brownish solid. It was suspended in 1,4-dioxane (0.6 mL) and HCl in 1,4-dioxane (1 mL, 4 M) was added and stirred for 2 h. All volatiles were removed under reduced pressure and the title compound could be obtained (3 mg, 4.9 µmol, 6 % yield).

LCMS (method A, ESI): R_t = 2.966 min, m/z = 576.30 $[M + H]^+$.

HRMS: calculated = 576,31152 $[M + H]^+$, found = 576,3109 $[M + H]^+$.

(*S*)-*N*-(1-(6,7-dihydrothieno[3,2-*c*]pyridin-5(*4H*)-yl)-1-oxopentan-2-yl)-3-(6-fluoro-2-methylpyridin-3-yl)-5-((2-imino-3-methyl-2,3-dihydro-*1H*-imidazol-1-yl)methyl)benzamide [11l]

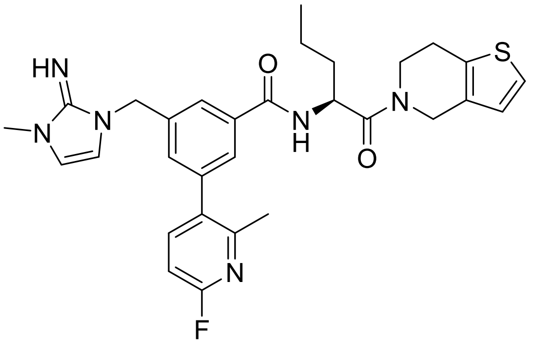

General procedure 2 was followed using **10b** (50 mg, 94.3 µmol) and (6-fluoro-2-methylpyridin-3-yl)boronic acid (29.2 mg, 188.5 µmol) in 1,4-dioxane/H_2O (4:1) (4 mL) to obtain the title compound (11 mg, 19.6 µmol, 21 % yield).

^{1}H NMR (600 MHz, DMSO): δ 8.79 – 8.65 (m, 1H), 7.98 – 7.80 (m, 5H), 7.54 (d, *J* = 8.9 Hz, 1H), 7.33 (dd, *J* = 11.4, 5.1 Hz, 1H), 7.15 (dd, *J* = 17.6, 5.2 Hz, 2H), 7.06 (d, *J* = 2.2 Hz, 1H), 6.89 (dd, *J* = 8.0, 5.2 Hz, 1H), 5.21 (s, 2H), 5.06 – 4.94 (m, 1H), 4.79 – 4.34 (m, 2H), 3.98 – 3.69 (m, 2H), 3.46 (s, 3H), 2.97 – 2.74 (m, 2H), 2.36 (s, 3H), 1.77 – 1.61 (m, 2H), 1.46 – 1.31 (m, 2H), 0.94 – 0.82 (m, 3H).

^{13}C NMR (151 MHz, DMSO): δ 170.67 (s), 165.46 (d, *J* = 6.3 Hz), 162.35 (s), 160.78 (s), 154.25 (d, *J* = 14.6 Hz), 145.76 (s), 143.32 (d, *J* = 8.1 Hz), 138.63 (s), 135.70 (s), 134.82 (d, *J* = 16.1 Hz), 133.28 (d, *J* = 4.3 Hz), 132.52 (d, *J* = 7.3 Hz), 131.17 (d, *J* = 14.0 Hz), 127.61 (s), 126.58 (d, *J* = 16.0 Hz), 125.44 (s), 123.68 (s), 117.50 (s), 115.31 (d, *J* = 67.4 Hz), 106.73 (d, *J* = 37.6 Hz), 49.40 (s), 47.83 (s), 42.98 (s), 42.44 (s), 33.41 (d, *J* = 36.1 Hz), 32.75 (s), 25.30 (s), 22.63 (s), 18.76 (d, *J* = 6.3 Hz), 13.72 (s).

LCMS (method A, ESI): R_t = 3.793 min, m/z = 576.30 $[M + H]^+$.

HRMS: calculated = 561,24425 [M + H]+, found = 561,2444 [M + H]+.

3-((*1H*-imidazol-1-yl)methyl)-5-(1-ethyl-3-(trifluoromethyl)-*1H*-pyrazol-4-yl)benzoic acid [13]

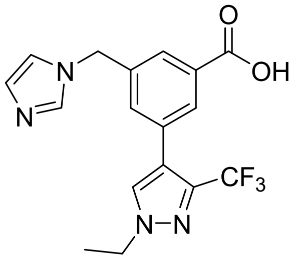

General procedure 2 was followed using **8a** (250 mg, 0.89 mmol) and (1-ethyl-3-(trifluoromethyl)-1H-pyrazol-4-yl)boronic acid (369.9 mg, 1.78 mmol) in 1,4-dioxane/H_2O (4:1) (20 mL) to obtain the title compound (301 mg, 826.2 µmol, 92 % yield, purity approx. 75 %).

^{1}H NMR (600 MHz, DMSO): δ 9.29 (s, 1H), 8.32 (s, 1H), 7.98 (dd, *J* = 7.6, 4.8 Hz, 2H), 7.84 (t, *J* = 1.7 Hz, 1H), 7.72 (t, *J* = 1.6 Hz, 1H), 7.66 (s, 1H), 5.56 (s, 2H), 4.26 (q, *J* = 7.3 Hz, 2H), 1.45 (t, *J* = 7.3 Hz, 3H).

LCMS (method A, ESI): R_t = 3.235 min, m/z = 365.05 [M + H]+.

***tert*-butyl (*S*)-(1-(6,7-dihydrothieno[3,2-*c*]pyridin-5(*4H*)-yl)-1-oxopent-4-yn-2-yl)carbamate [15]**

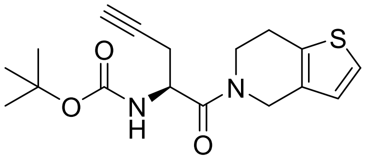

General procedure 1 was followed using (*S*)-2-((tert-butoxycarbonyl)amino)pent-4-ynoic acid (500 mg, 2.34 mmol) and 4,5,6,7-tetrahydrothieno[3,2-c]pyridine hydrochloride (412 mg, 2.34 mmol) in DMF (20 mL) to obtain the title compound (715 mg, 2.14 mmol, 91 % yield).

^{1}H NMR (400 MHz, $CDCl_3$): δ 7.14 (d, *J* = 5.2 Hz, 1H), 6.79 (dd, *J* = 13.1, 5.1 Hz, 1H), 5.47 (dd, *J* = 26.2, 8.8 Hz, 1H), 4.91 (s, 1H), 4.70 (ddd, *J* = 42.8, 26.5, 16.7 Hz, 2H), 4.10 – 3.75 (m, 2H), 2.90 (dd, *J* = 20.1, 14.6 Hz, 2H), 2.74 – 2.53 (m, 2H), 1.58 (s, 1H), 1.44 (s, *J* = 3.8 Hz, 9H).

LCMS (method A, ESI): R_t = 4.693 min, m/z = 357.05 [M + Na]+.

(*S*)-3-((*1H*-imidazol-1-yl)methyl)-*N*-(1-(6,7-dihydrothieno[3,2-*c*]pyridin-5(*4H*)-yl)-1-oxopent-4-yn-2-yl)-5-(1-ethyl-3-(trifluoromethyl)-*1H*-pyrazol-4-yl)benzamide [16]

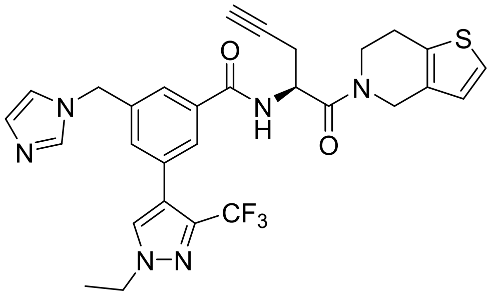

15 (333.2 mg, 0.996 mmol) were dissolved in DCM (14 mL) and TFA (6 mL) were added to the solution. After stirring for 1 hour at room temperature the mixture was concentrated in vacuo. With no further purification **general procedure 1** was followed using **13** (363. mg, 0.996 mmol) in DMF (20 mL) obtaining the title compound as a light brown solid (307 mg, 0,529 mmol, 53 % yield).

^{1}H NMR (600 MHz, DMSO): δ 8.93 (dd, *J* = 73.5, 8.4 Hz, 1H), 8.20 (d, *J* = 11.9 Hz, 1H), 7.89 – 7.71 (m, 3H), 7.41 – 7.27 (m, 2H), 7.19 (d, *J* = 6.3 Hz, 1H), 6.94 – 6.80 (m, 2H), 5.28 (d, *J* = 11.2 Hz, 2H), 5.18 (ddd, *J* = 44.8, 15.1, 8.0 Hz, 1H), 4.75 – 4.43 (m, 2H), 4.25 (q, *J* = 7.3 Hz, 2H), 3.94 – 3.69 (m, 2H), 2.92 – 2.58 (m, 5H), 1.43 (dd, *J* = 8.4, 6.2 Hz, 3H).

^{13}C NMR (151 MHz, DMSO): δ 168.50 (s), 165.23 (s), 138.39 (s), 136.32 (d, *J* = 36.0 Hz), 134.40 (s), 133.06 (s), 132.37 (d, *J* = 46.1 Hz), 131.89 (s), 131.48 (s), 131.04 (d, *J* = 13.2 Hz), 130.17 (s), 126.80 (s), 125.98 (s), 125.34

(s), 124.36 – 124.21 (m), 123.64 (s), 122.55 (s), 120.76 (s), 119.83 (s), 80.92 (s), 72.59 (s), 49.20 (s), 48.48 (s), 47.23 (s), 42.95 (s), 42.60 (s), 25.11 (s), 21.43 (d, *J* = 27.8 Hz), 15.04 (s).

LCMS (method A, ESI): R_t = 3.295 min, m/z = 581.15 $[M + H]^+$.

3-((*1H*-imidazol-1-yl)methyl)-*N*-((*S*)-1-(6,7-dihydrothieno[3,2-*c*]pyridin-5(*4H*)-yl)-1-oxo-3-(1-(6-(5-((3a*S*,4*S*,6a*R*)-2-oxohexahydro-*1H*-thieno[3,4-*d*]imidazol-4-yl)pentanamido)hexyl)-*1H*-1,2,3-triazol-4-yl)propan-2-yl)-5-(1-ethyl-3-(trifluoromethyl)-*1H*-pyrazol-4-yl)benzamide [17a]

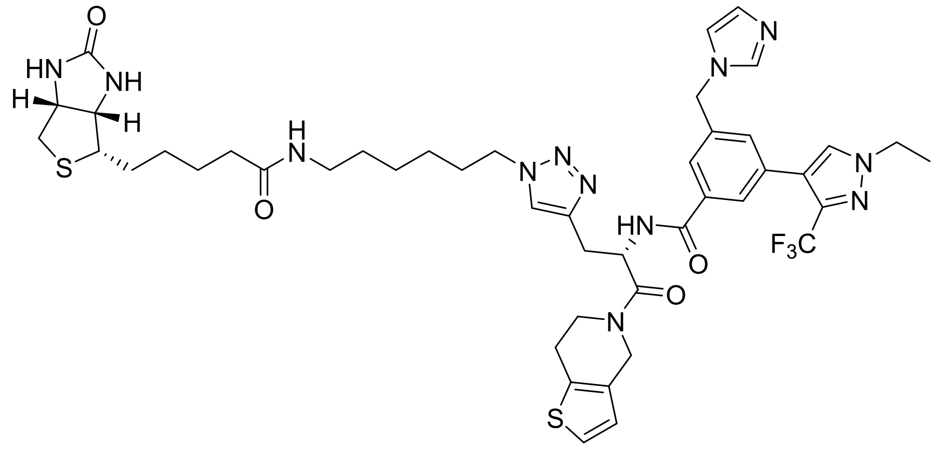

Copper sulphate pentahydrate (2.7 mg, 10.7 µmol) and sodium L-ascorbate (4.2 mg, 21.4 µmol) were suspended in Water (1 mL). **16** (31.0 mg; 53.4 µmol) and 6-azidohexan-1-amine (7.6 mg, 53.4 µmol) in acetonitrile (9 mL) and Methanol (6 mL) were added to the yellow suspension. After stirring overnight at room temperature, the mixture was concentrated in vacuo. The residue was dissolved in DMF (1.5 mL), [(+)-Biotin-N-Hydroxysuccinimide-Ester] (20.0 mg, 58,7 µmol) and DIPEA (20.5µL, 117 µmol) was added. After stirring overnight at room temperature, the brownish mixture was concentrated in vacuo and purified by flash chromatography, followed by RP-flash chromatography to obtain the title compound (9 mg, 9.5 µmol, 18 % yield).

^{1}H NMR (600 MHz, DMSO): δ 9.01 – 8.84 (m, 1H), 8.21 (s, 1H), 7.84 – 7.67 (m, 4H), 7.39 – 7.29 (m, 2H), 7.19 (s, 1H), 6.93 – 6.80 (m, 2H), 6.37 (d, *J* = 37.7 Hz, 2H), 5.34 – 5.21 (m, 3H), 4.67 (dd, *J* = 27.6, 16.3 Hz, 1H), 4.49 (dd, *J* = 67.0, 16.8 Hz, 1H), 4.31 – 4.09 (m, 6H), 3.81 (d, *J* = 49.1 Hz, 2H), 3.22 – 3.01 (m, 3H), 2.94 (s, 2H), 2.78 (t, *J* = 27.0 Hz, 2H), 2.62 -2.55 (m, 1H), 2.03 (s, 2H), 1.68 – 1.41 (m, 9H), 1.27-1.10 (m 10H).

LCMS (method A, ESI): R_t = 3.582 min, m/z = 949.40 $[M + H]^+$.

HRMS: calculated = 949,39355 $[M + H]^+$, found = 949,3956 $[M + H]^+$.

***S*)-3-((*1H*-imidazol-1-yl)methyl)-*N*-(1-(6,7-dihydrothieno[3,2-*c*]pyridin-5(*4H*)-yl)-1-oxo-3-(1-propyl-*1H*-1,2,3-triazol-4-yl)propan-2-yl)-5-(1-ethyl-3-(trifluoromethyl)-*1H*-pyrazol-4-yl)benzamide [17b]**

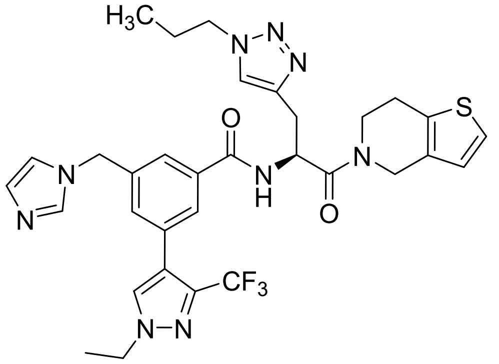

1-Iodopropane (23.3 µL, 0.24 mmol) and sodium azide (19.5 mg, 0.3 mmol) were dissolved in acetone (2.4 mL) and stirred overnight at 50 °C under light exclusion. Copper sulphate pentahydrate (2.7 mg, 10.7 µmol) and sodium L-ascorbate (4.2 mg, 21.4 µmol) were suspended in Water (1 mL). **16** (31.0 mg, 53.4 µmol) in 1,4-dioxane (9 mL) and the freshly prepared 1-azidopropane in acetone were added to the yellow suspension. After stirring for 48 h at 35 °C the mixture was concentrated in vacuo purified by flash chromatography, followed by RP-flash chromatography to obtain the title compound (12 mg, 18 µmol, 34 % yield).

^{1}H NMR (600 MHz, DMSO): δ 8.92 (dd, *J* = 61.4, 8.0 Hz, 1H), 8.21 (d, *J* = 5.9 Hz, 1H), 7.82 – 7.71 (m, 4H), 7.40 – 7.29 (m, 3H), 6.85 (dd, *J* = 38.1, 5.1 Hz, 2H), 5.35 – 5.21 (m, 3H), 4.72 – 4.64 (m, 1H), 4.58 – 4.43 (m, 1H), 4.26 (q, *J* = 7.3 Hz, 2H), 4.17 (dt, *J* = 26.3, 6.8 Hz, 2H), 3.91 – 3.70 (m, 2H), 3.23 – 3.00 (m, 2H), 2.87 – 2.68 (m, 2H), 1.72 – 1.61 (m, 2H), 1.44 (t, *J* = 7.3 Hz, 3H), 0.69 (q, *J* = 7.2 Hz, 3H).

^{13}C NMR (151 MHz, DMSO): δ 169.56 (d, *J* = 15.9 Hz), 165.38 (d, *J* = 10.1 Hz), 142.68 (d, *J* = 33.6 Hz), 138.37 (s), 136.71 – 136.61 (m), 136.42 (s), 136.19 (s), 134.57 (d, *J* = 14.6 Hz), 132.40 (dd, *J* = 120.7, 83.1 Hz), 131.48 (s), 130.97 (d, *J* = 8.5 Hz), 130.11 (s), 126.80 (s), 125.94 (s), 125.34 (s), 124.98 (s), 123.64 (s), 122.79 (d, *J* = 11.9 Hz), 122.57 (s), 120.79 (s), 119.90 (s), 50.66 (d, *J* = 4.6 Hz), 49.48 (s), 47.25 (s), 44.98 (s), 42.96 (s), 42.52 (s), 27.93 (d, *J* = 22.0 Hz), 25.09 (s), 23.09 (d, *J* = 4.3 Hz), 15.07 (s), 10.56 (d, *J* = 5.3 Hz).

LCMS (method A, ESI): R_t = 3.837 min, m/z = 666.25 [M + H]$^+$.

HRMS: calculated = 666,25810 [M + H]$^+$, found = 666,2587 [M + H]$^+$.

(*S*)-3-((*1H*-imidazol-1-yl)methyl)-N-(1-(6,7-dihydrothieno[3,2-*c*]pyridin-5(*4H)*-yl)-1-oxo-5-(pyridin-2-yl)pent-4-yn-2-yl)-5-(1-ethyl-3-(trifluoromethyl)-*1H*-pyrazol-4-yl)benzamide [18]

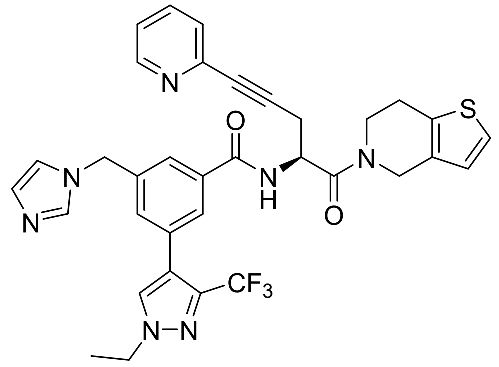

16 (59 mg, 102 µmol), 2-bromopyridine (10.2 µL, 106.7 µmol), bis(triphenylphosphine)palladium(II)-dichloride (3.6 mg, 5.1 µmol), copper(I) iodide (13.6 mg, 71.µmol), in TEA (0.5 mL) and 1,4-dioxane (2 mL) were stirred for 2 h at 60 °C. The mixture was concentrated in vacuo purified by flash chromatography, followed by RP-flash chromatography to obtain the title compound (37 mg, 56.3 µmol, 55 % yield).

^{1}H NMR (600 MHz, DMSO): δ 9.19 – 8.91 (m, 1H), 8.49 (s, 1H), 8.27 – 8.17 (m, *J* = 28.6 Hz, 2H), 7.89 – 7.68 (m, 3H), 7.43 – 7.14 (m, 5H), 6.94 – 6.79 (m, 1H), 5.66 – 5.20 (m, 3H), 4.82 – 4.43 (m, 2H), 4.24 (q, *J* = 7.2 Hz, 2H), 4.02 – 3.75 (m, 2H), 3.04 – 2.71 (m, 5H), 1.42 (dd, *J* = 9.3, 5.0 Hz, 3H).

^{13}C NMR (151 MHz, DMSO): δ 168.89 – 168.07 (m), 165.45 (d, *J* = 5.0 Hz), 149.78 (s), 142.56 (s), 136.46 (s), 136.30 – 136.16 (m), 134.52 – 134.41 (m), 134.38 (s), 132.45 (dd, *J* = 122.1, 65.7 Hz), 131.65 – 131.59 (m), 131.45 (s), 131.02 (s), 130.98 – 130.91 (m), 130.62 – 130.40 (m), 128.84 – 128.64 (m), 127.00 (s), 126.21 (s), 125.35 (s), 124.99 (s), 123.59 (d, *J* = 26.9 Hz), 122.53 (s), 120.75 (s), 119.76 (d, *J* = 21.9 Hz), 86.72 (d, *J* = 55.7 Hz), 48.72 (s), 48.48 (s), 47.25 (s), 45.19 (s), 43.06 (s), 42.68 (s), 25.14 (s), 22.23 (d, *J* = 28.3 Hz), 15.02 (d, *J* = 12.5 Hz).

LCMS (method A, ESI): R_t = 3.802 min, m/z = 658.15 [M + H]$^+$.

HRMS: calculated = 658,22066 [M + H]$^+$, found = 658,2225 [M + H]$^+$.

(*S*)-*N*-(1-(6,7-dihydrothieno[3,2-*c*]pyridin-5(*4H*)-yl)-1-oxopentan-2-yl)-3-((4,5-dimethyl-*1H*-imidazol-1-yl)methyl)-5-(1-ethyl-3-(trifluoromethyl)-*1H*-pyrazol-4-yl)benzamide [LH214]

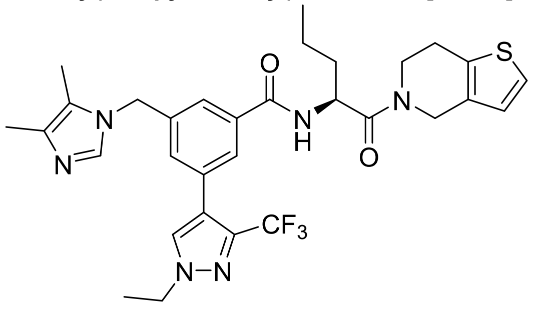

General procedure 2 was followed using (*S*)-3-bromo-*N*-(1-(6,7-dihydrothieno[3,2-*c*]pyridin-5(*4H*)-yl)-1-oxopentan-2-yl)-5-((4,5-dimethyl-*1H*-imidazol-1-yl)methyl)benzamide (66 mg, 124.7 µmol) and 1-ethyl-3-

(trifluoromethyl)-*1H*-pyrazol-4-yl)boronic acid (51.8 mg, 249 µmol) in 1,4-dioxane/H_2O (4:1) (2.5 mL) to obtain the title compound (10.6 mg, 17.3 µmol, 14 % yield).

^{1}H NMR (600 MHz, DMSO): δ 8.69 (dd, *J* = 56.8, 8.0 Hz, 1H), 8.21 (s, 1H), 7.82 (d, *J* = 26.4 Hz, 1H), 7.66 (d, *J* = 26.8 Hz, 1H), 7.57 (s, 1H), 7.33 (t, *J* = 4.8 Hz, 1H), 7.12 (d, *J* = 6.8 Hz, 1H), 6.89 (d, *J* = 5.0 Hz, 1H), 5.18 (s, 2H), 4.98 (d, *J* = 54.1 Hz, 1H), 4.77 – 4.39 (m, *J* = 214.3 Hz, 2H), 4.24 (q, *J* = 7.3 Hz, 2H), 3.95 – 3.73 (m, *J* = 125.5 Hz, 2H), 2.94 – 2.74 (m, *J* = 49.4, 33.4 Hz, 2H), 2.01 (s, 3H), 1.95 (s, 3H), 1.75 – 1.59 (m, *J* = 60.4 Hz, 2H), 1.45 – 1.29 (m, 5H), 0.93 – 0.83 (m, 3H).

LCMS (method A, ESI): R_t = 3.743 min, m/z = 613.30 [M + H]$^+$.

HRMS: calculated = 613,25671 [M + H]$^+$, found = 613,2573 [M + H]$^+$.

(*R*)-3-((1H-imidazol-1-yl)methyl)-*N*-(1-(6,7-dihydrothieno[3,2-*c*]pyridin-5(*4H*)-yl)-1-oxopentan-2-yl)-5-(1-ethyl-3-(trifluoromethyl)-*1H*-pyrazol-4-yl)benzamide [LH224]

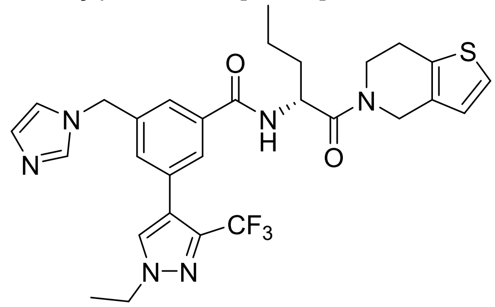

General procedure 2 was followed using (*R*)-3-((*1H*-imidazol-1-yl)methyl)-5-bromo-*N*-(1-(6,7-dihydrothieno[3,2-*c*]pyridin-5(4*H*)-yl)-1-oxopentan-2-yl)benzamide (22 mg, 43.9 µmol) and 1-ethyl-3-(trifluoromethyl)-*1H*-pyrazol-4-yl)boronic acid (18.3 mg, 88 µmol) in 1,4-dioxane/H_2O (4:1) (2 mL) to obtain the title compound (17 mg, 29.0 µmol, 66 % yield).

^{1}H NMR (600 MHz, DMSO): δ 8.76 – 8.60 (m, 1H), 8.20 (d, *J* = 3.6 Hz, 1H), 7.86 – 7.73 (m, 3H), 7.39 – 7.31 (m, 2H), 7.19 (s, 1H), 6.89 (dd, *J* = 11.4, 6.3 Hz, 2H), 5.27 (s, 2H), 5.04 – 4.92 (m, 1H), 4.76 – 4.38 (m, 2H), 4.24 (q, *J* = 7.3 Hz, 3H), 3.95 – 3.73 (m, 2H), 2.94 – 2.74 (m, 3H), 1.74 – 1.60 (m, 3H), 1.46 – 1.28 (m, 5H), 0.92 – 0.82 (m, *J* = 19.2, 7.3 Hz, 3H).

LCMS (method D, ESI): R_t = 6.281 min, m/z = 585.25 [M + H]$^+$.

HRMS: calculated = 585,22541 [M + H]$^+$, found = 585,2269 [M + H]$^+$.

(*R*)-*N*-(1-(6,7-dihydrothieno[3,2-*c*]pyridin-5(*4H*)-yl)-1-oxopentan-2-yl)-3-((4,5-dimethyl-*1H*-imidazol-1-yl)methyl)-5-(1-ethyl-3-(trifluoromethyl)-*1H*-pyrazol-4-yl)benzamide [LH222]

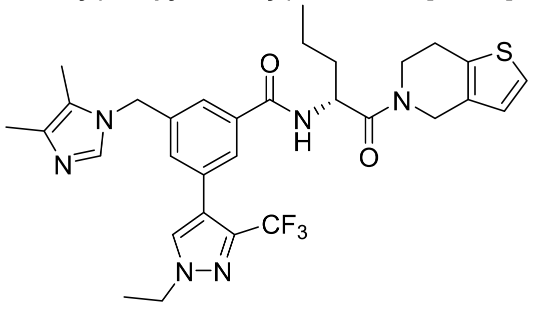

General procedure 2 was followed using (*R*)-3-bromo-*N*-(1-(6,7-dihydrothieno[3,2-*c*]pyridin-5(*4H*)-yl)-1-oxopentan-2-yl)-5-((4,5-dimethyl-*1H*-imidazol-1-yl)methyl)benzamide (28 mg, 52.9 µmol) and 1-ethyl-3-(trifluoromethyl)-*1H*-pyrazol-4-yl)boronic acid (22.0 mg, 105.8 µmol) in 1,4-dioxane/H_2O (4:1) (2 mL) to obtain the title compound (24 mg, 39.2 µmol, 75 % yield).

^{1}H NMR (600 MHz, DMSO): δ 9.02 (d, *J* = 8.7 Hz, 1H), 8.79 – 8.64 (m, 1H), 8.24 (d, *J* = 10.5 Hz, 1H), 7.97 – 7.70 (m, 2H), 7.33 (dd, *J* = 10.6, 5.8 Hz, 1H), 7.30 – 7.26 (m, 1H), 6.89 (d, *J* = 5.1 Hz, 1H), 5.48 (s, 2H), 5.03 – 4.95 (m, 1H), 4.78 – 4.37 (m, 2H), 4.25 (q, *J* = 7.3 Hz, 2H), 3.97 – 3.83 (m, 2H), 2.94 – 2.71 (m, 2H), 2.20 (s, 3H), 2.08 (s, 3H), 1.74 – 1.62 (m, 2H), 1.45 – 1.30 (m, 5H), 0.93 – 0.84 (m, 3H).

^{13}C NMR (151 MHz, DMSO): δ 170.73 (s), 165.54 (s), 135.46 (s), 135.26 – 135.02 (m), 134.02 (s), 132.57 (d, *J* = 12.4 Hz), 131.69 (s), 131.37 (s), 129.75 – 129.48 (m), 127.37 (s), 126.27 (s), 126.05 (s), 125.74 (s), 125.50 (s), 125.23 – 125.12 (m), 123.77 (s), 122.72 – 122.53 (m), 120.91 – 120.83 (m), 119.75 (s), 49.51 (s), 49.05 (s), 47.40 (s), 43.06 (s), 42.52 (s), 33.57 (s), 25.34 (s), 18.83 (s), 15.17 (s), 13.78 (s), 9.07 (s), 7.64 (s).

LCMS (method D, ESI): R_t = 6.486 min, m/z = 613.25 $[M + H]^+$.

HRMS: calculated = 613,25671 $[M + H]^+$, found = 613,258 $[M + H]^+$.

Analytical spectra and chromatograms of synthesized compounds

3

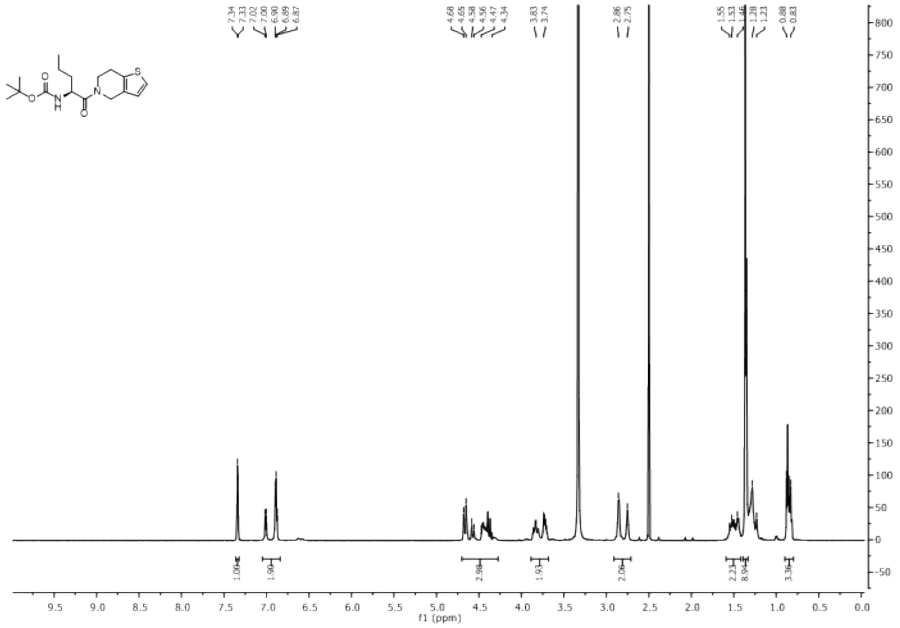

Figure S 1: ^{1}H-NMR of compound **3**.

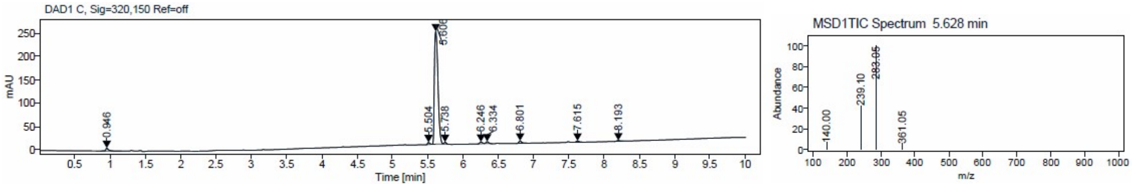

Figure S 2: LC/MS spectra of purified compound **3** at 320 nm and ESI mass spectrum.

4

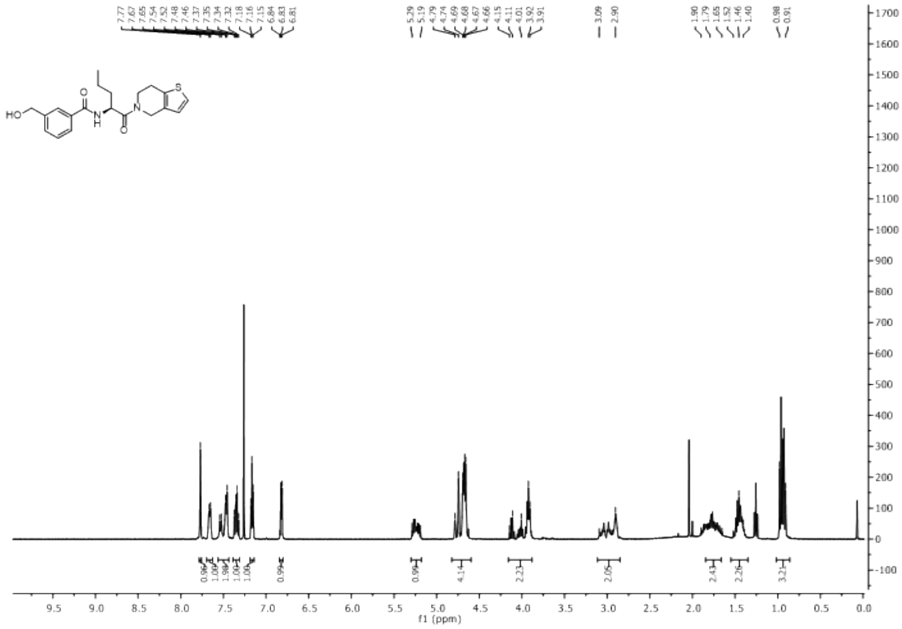

Figure S 3: ^{1}H-NMR of compound 4.

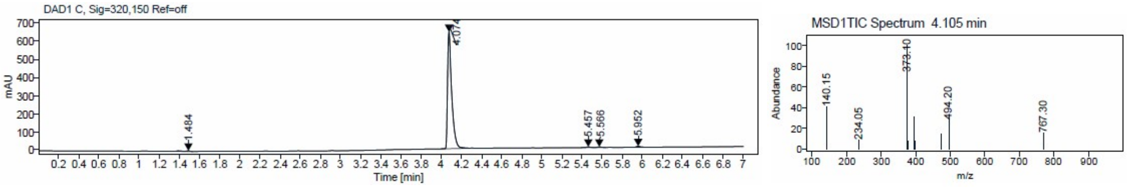

Figure S 4: LC/MS spectra of purified compound **4** at 320 nm and ESI mass spectrum.

5a

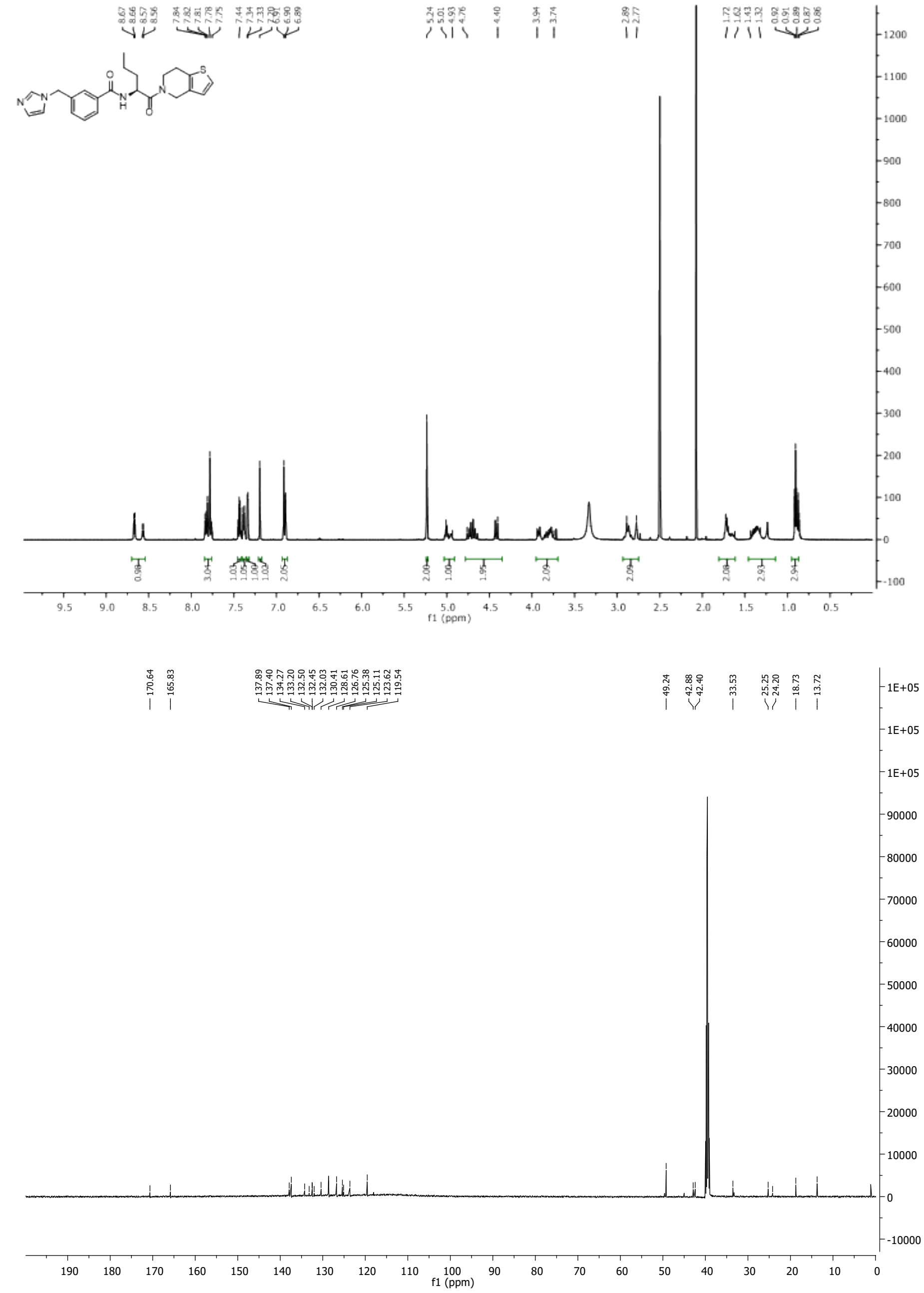

Figure S 5: ^{1}H- (top) and ^{13}C-NMR (bottom) of compound **5a**.

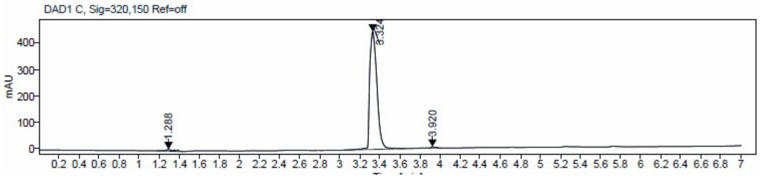

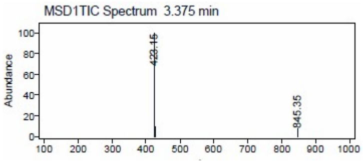

Figure S 6: LC/MS spectra of purified compound **5a** at 320 nm and ESI mass spectrum.

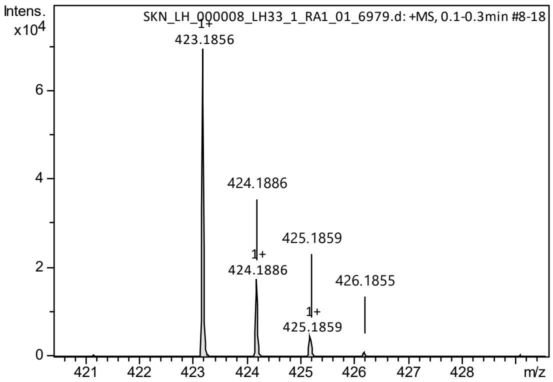

Figure S 7: High-resolution mass spectrum of compound **5a** with $[M+H]^{+}_{calc.}$= 423,18492 m/z.

5b

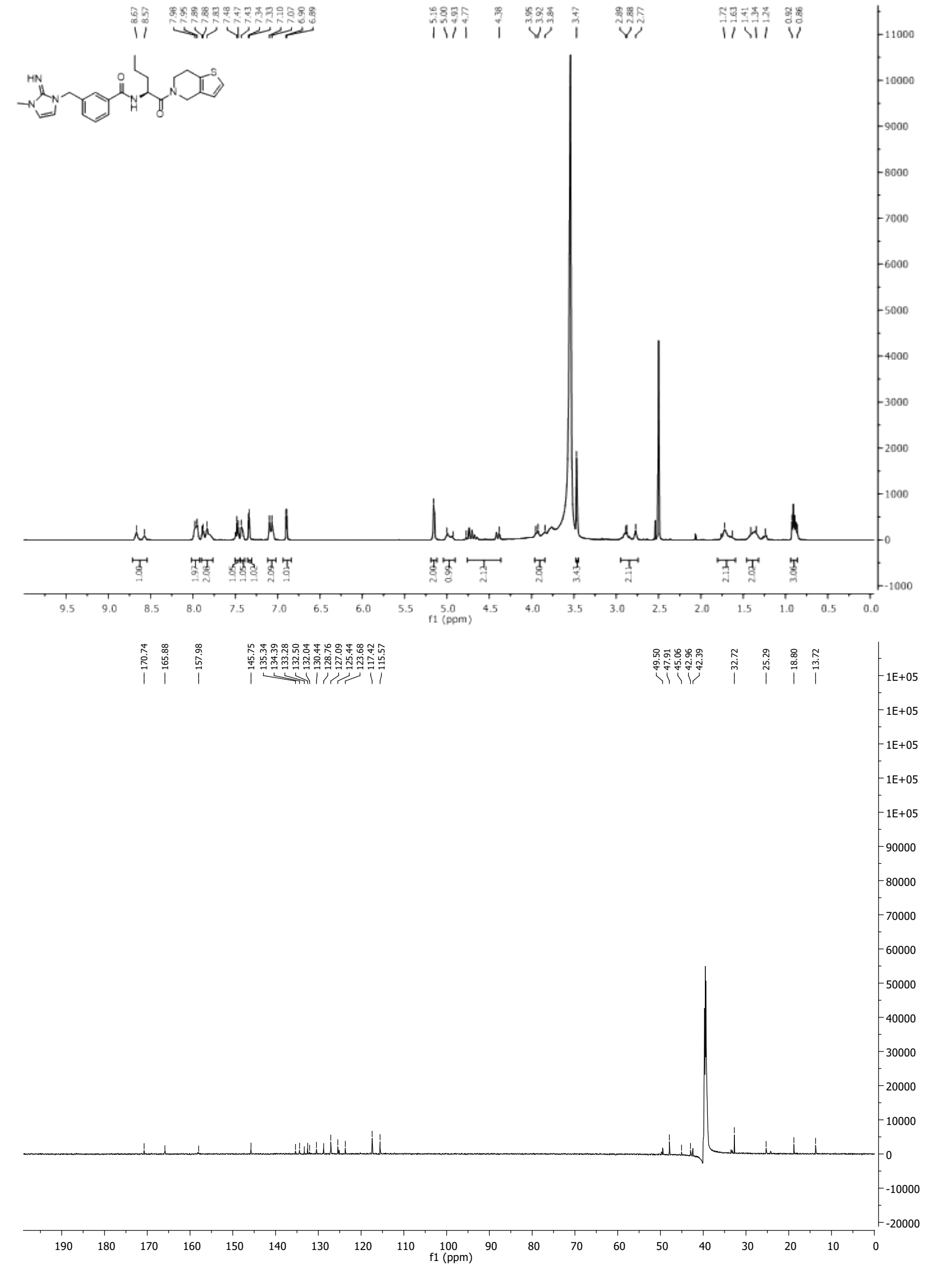

Figure S 81: ^{1}H- (top) and ^{13}C-NMR (bottom) of compound **5b**.

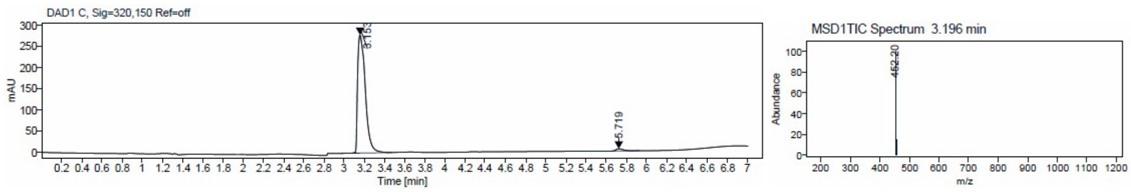

Figure S 9: LC/MS spectra of purified compound **5b** at 320 nm and ESI mass spectrum

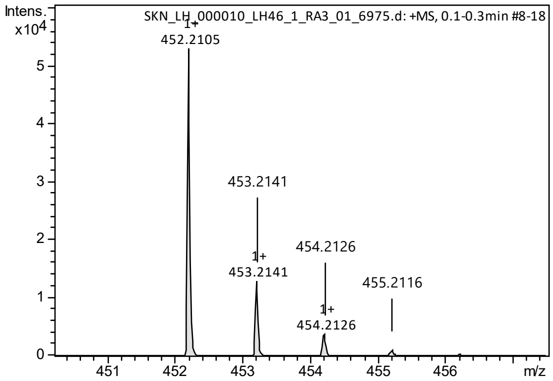

Figure S 10: High-resolution mass spectrum of compound **5b** with $[M+H]^{+}_{calc.}$= 452,21147m/z.

5c

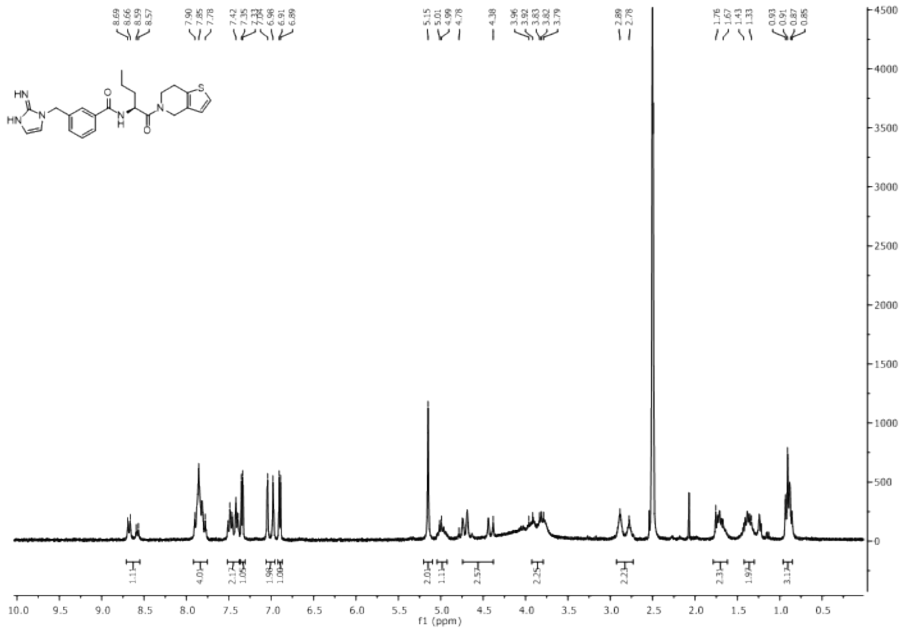

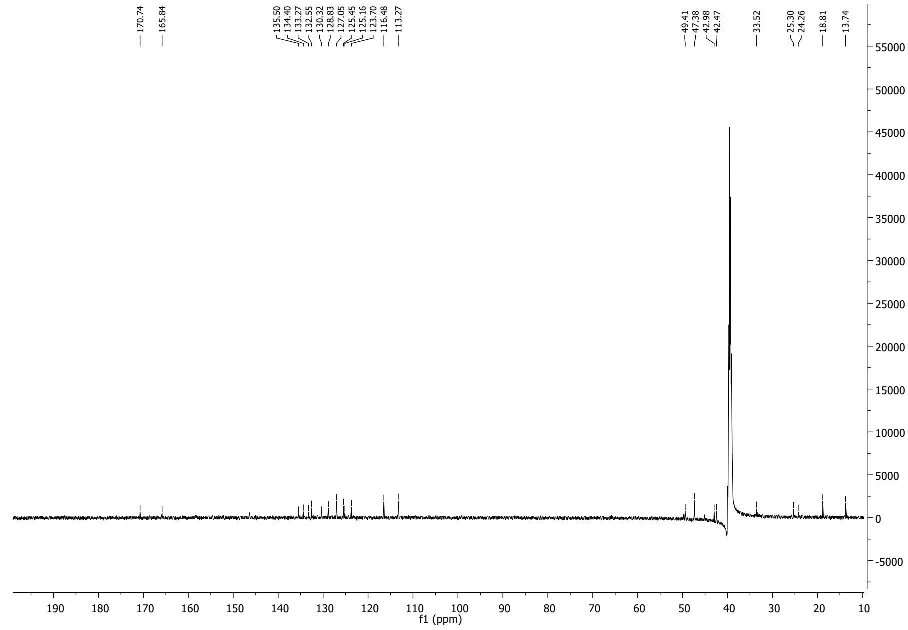

Figure S 11: ^{1}H- (top) and ^{13}C-NMR (bottom) of compound **5c**.

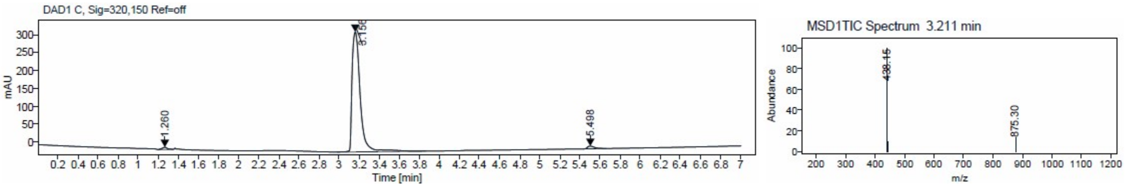

Figure S 12: LC/MS spectra of purified compound **5c** at 320 nm and ESI mass spectrum.

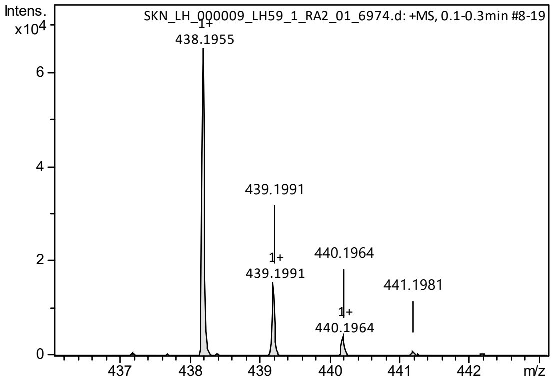

Figure S 13: High-resolution mass spectrum of compound **5c** with $[M+H]^{+}_{calc.}$= 438,19582 m/z.

11a

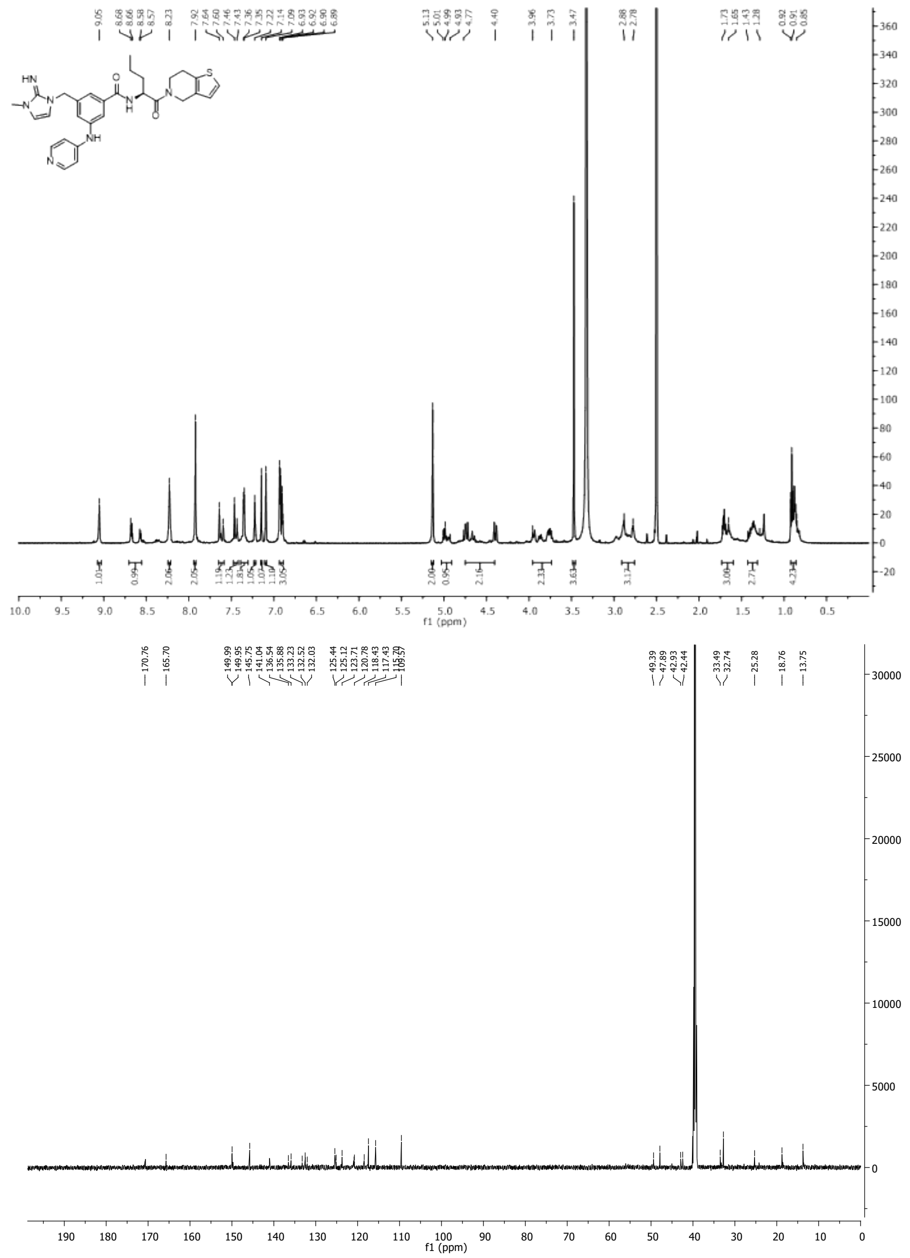

Figure S 14: ^{1}H- (top) and ^{13}C-NMR (bottom) of compound **11a**.

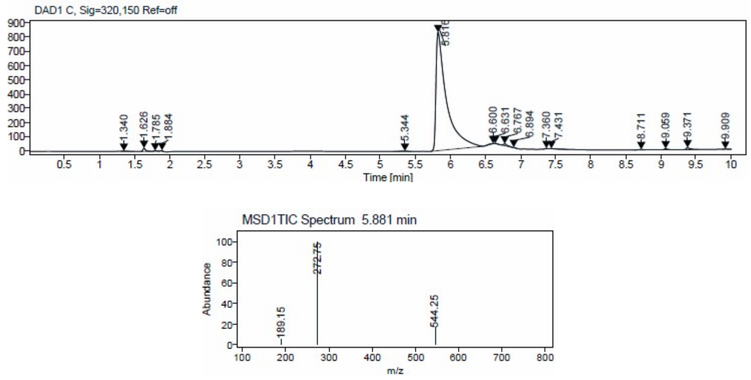

Figure S 15: LC/MS spectra of purified compound **11a** at 320 nm and ESI mass spectrum.

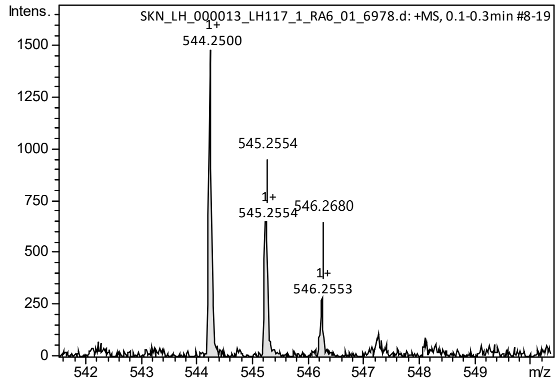

Figure S 16: High-resolution mass spectrum of compound **11a** with $[M+H]^+_{calc.}$ = 544,24892 m/z.

11b

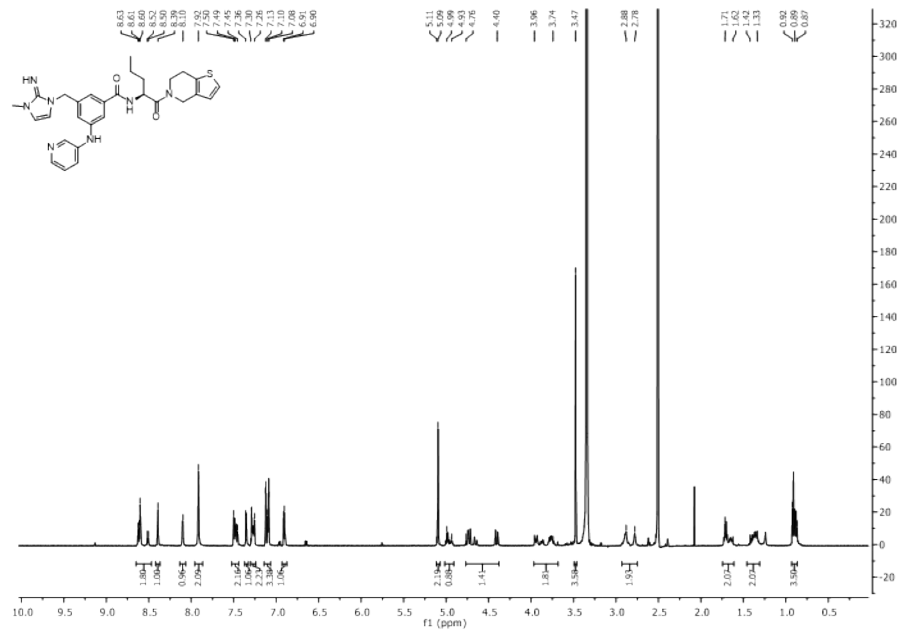

Figure S 17: H- NMR of compound **11b**.

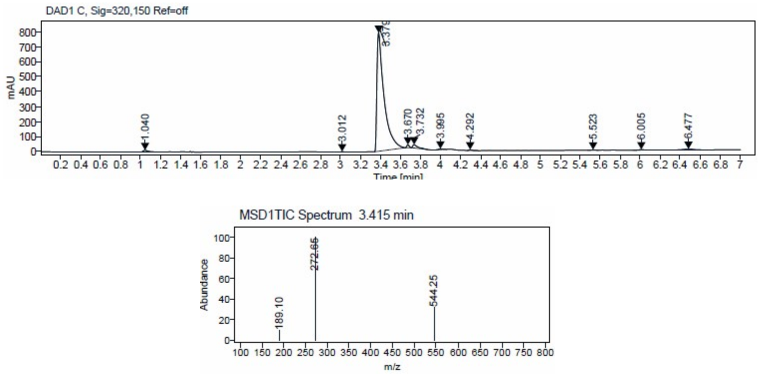

Figure S 18: LC/MS spectra of purified compound **11b** at 320 nm and ESI mass spectrum.

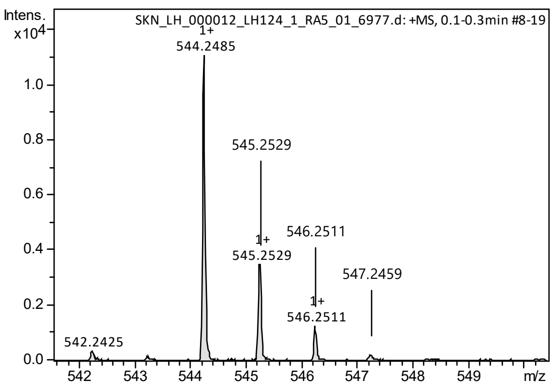

Figure S 19: High-resolution mass spectrum of compound **11b** with $[M+H]^+_{calc.}$= 544,24892 m/z.

11c

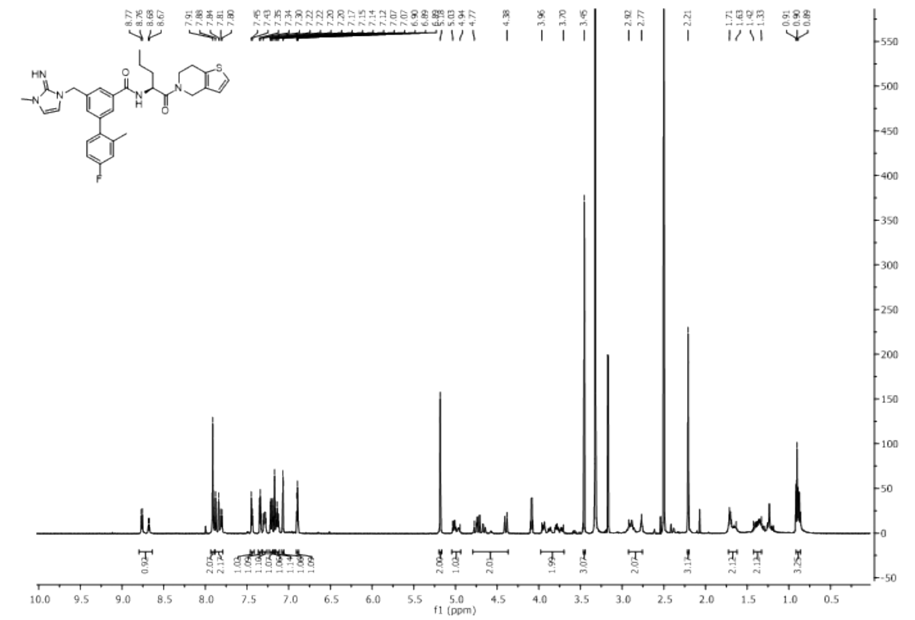

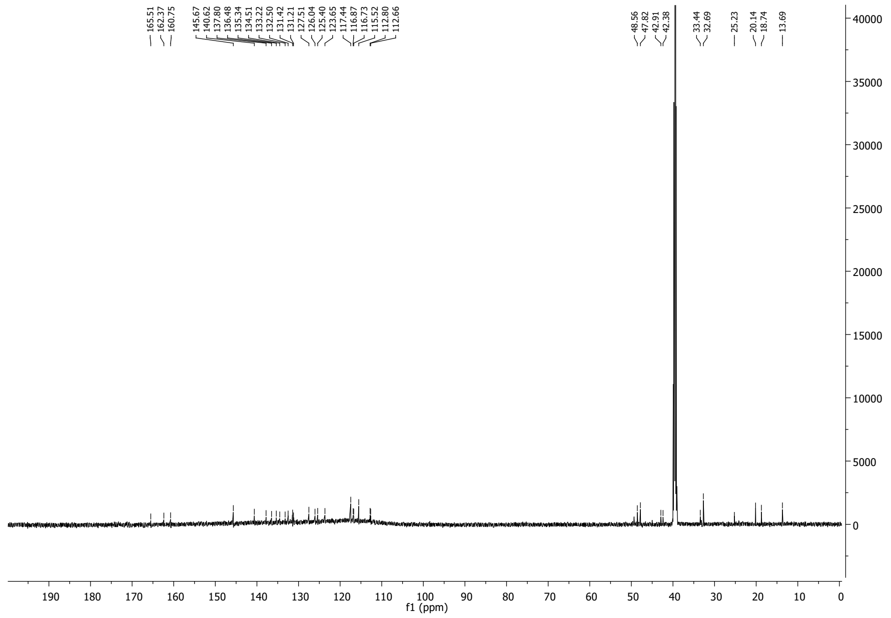

Figure S 20: ^{1}H- (top) and ^{13}C-NMR (bottom) of compound **11c**.

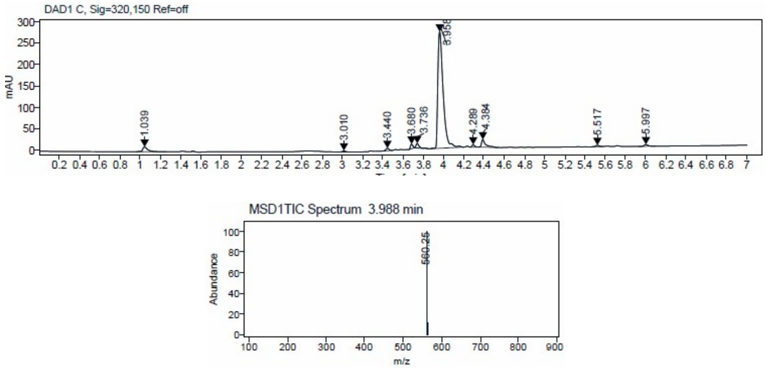

Figure S 21: LC/MS spectra of purified compound **11c** at 320 nm and ESI mass spectrum.

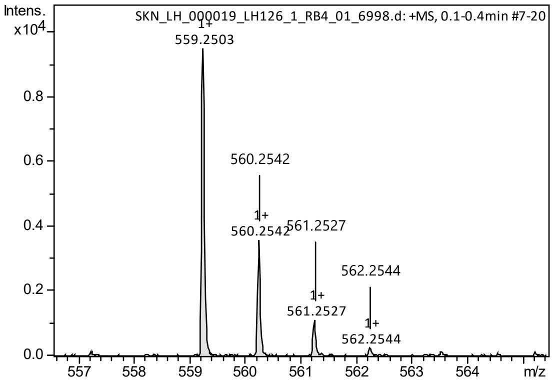

Figure S 22: High-resolution mass spectrum of compound **11c** with $[M+H]^{+}_{calc.}$= 560,24900 m/z.

11d

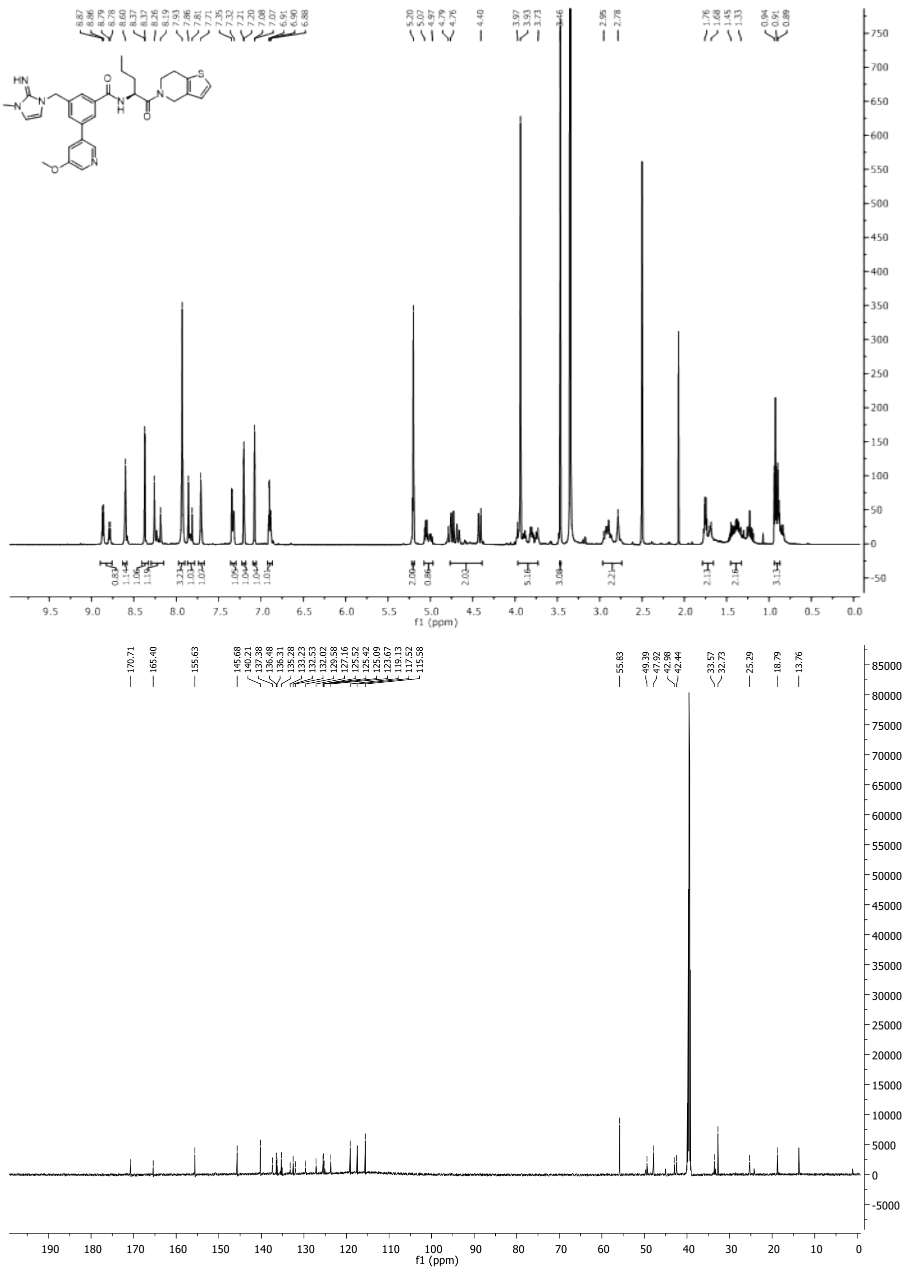

Figure S 23: ^{1}H- (top) and ^{13}C-NMR (bottom) of compound **11d**.

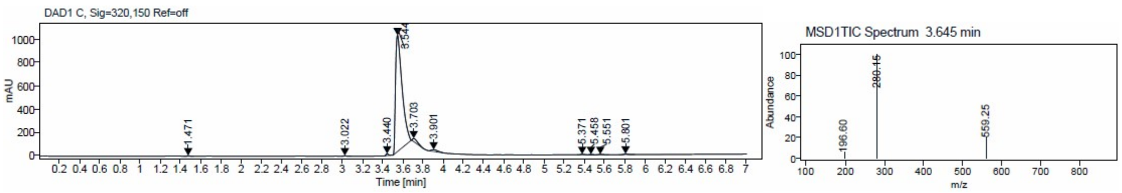

Figure S 24: LC/MS spectra of purified compound **11d** at 320 nm and ESI mass spectrum.

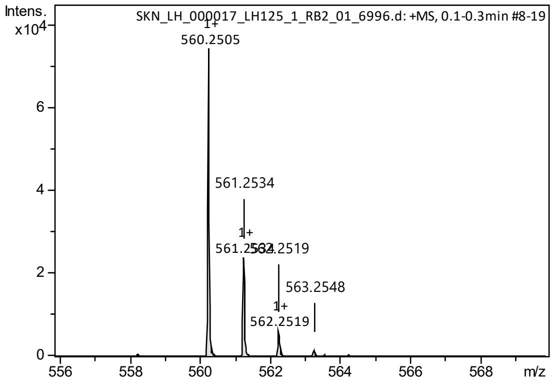

Figure S 25: High-resolution mass spectrum of compound **11d** with $[M+H]^{+}_{calc.}$= 559,24859 m/z.

11e

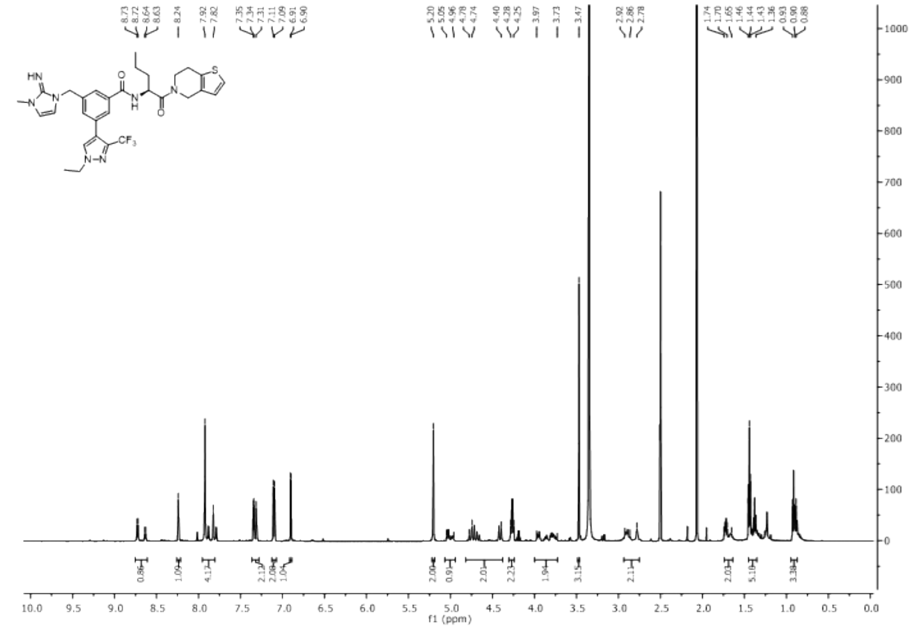

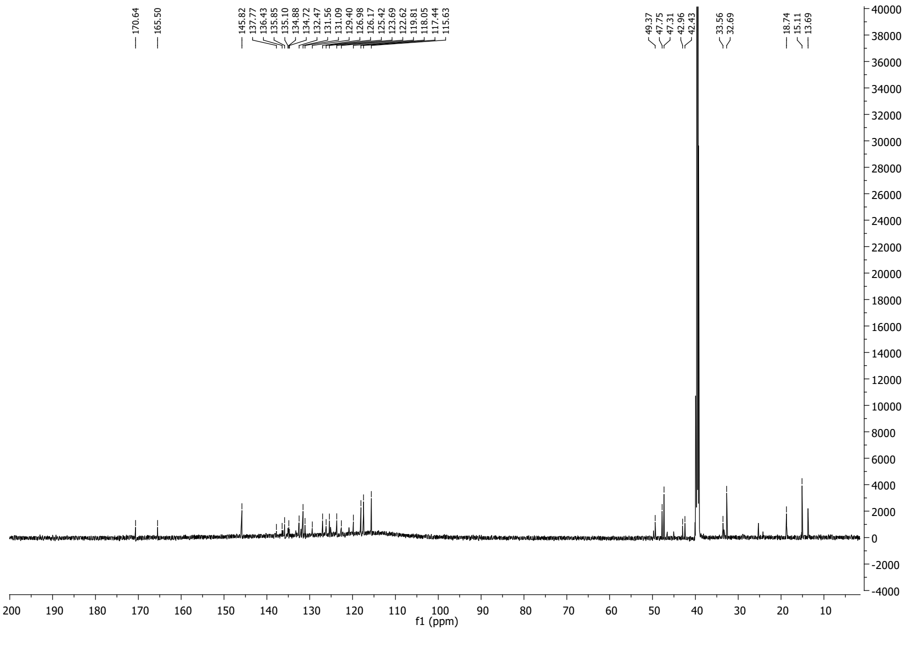

Figure S 26: ^{1}H- (top) and ^{13}C-NMR (bottom) of compound **11e**.

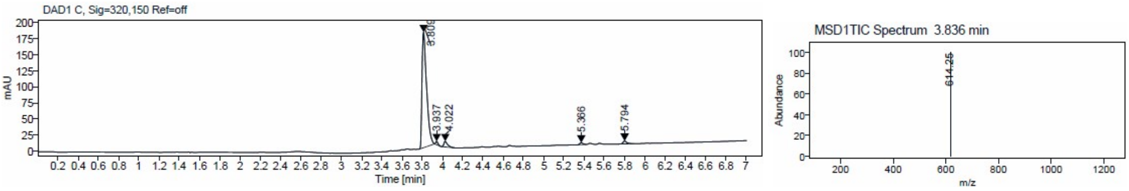

Figure S 27: LC/MS spectra of purified compound **11e** at 320 nm and ESI mass spectrum.

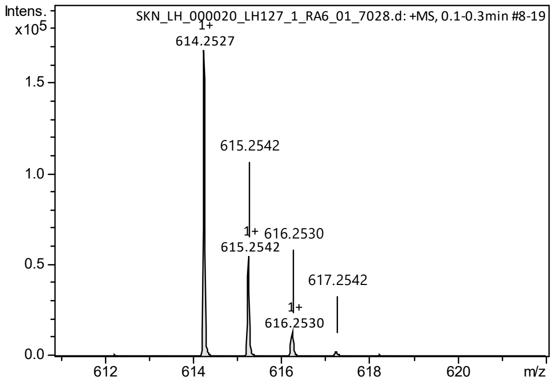

Figure S 28: High-resolution mass spectrum of compound **11e** with $[M+H]^{+}_{calc.}$= 614,25196 m/z.

11f

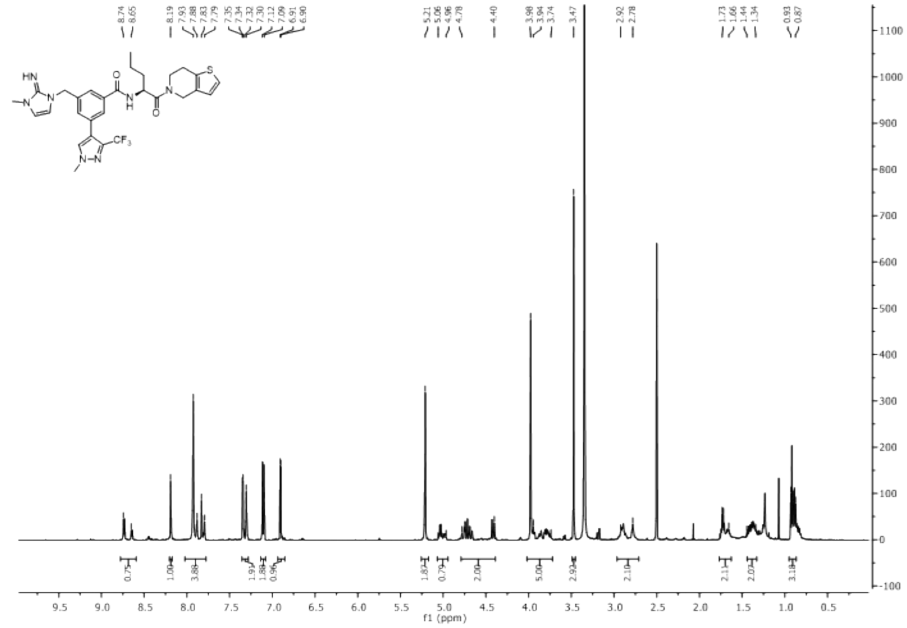

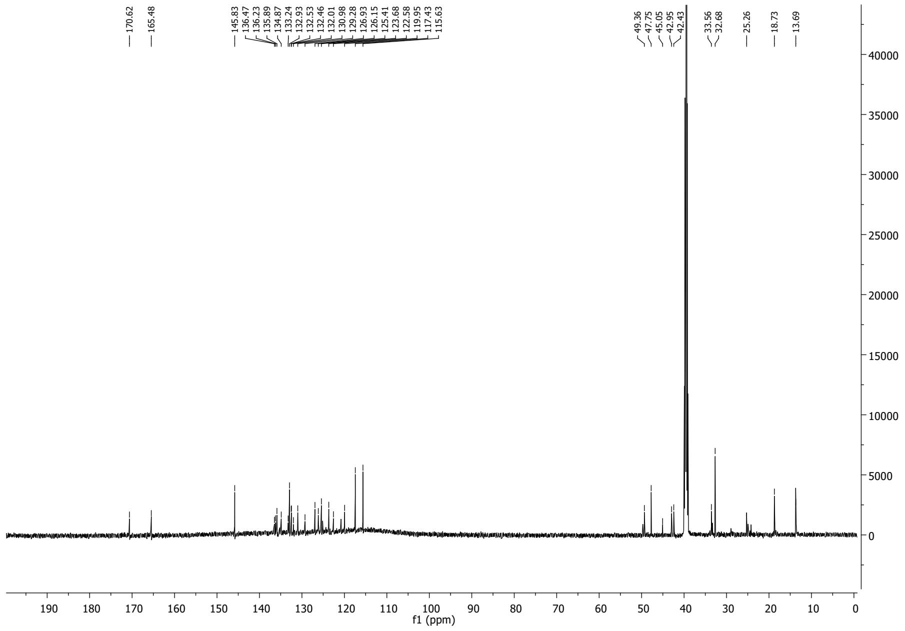

Figure S 29: ^{1}H- (top) and ^{13}C-NMR (bottom) of compound **11f**

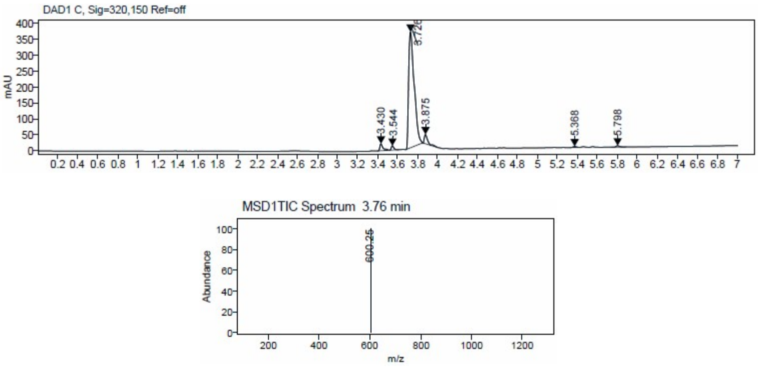

Figure S 30: LC/MS spectra of purified compound **11f** at 320 nm and ESI mass spectrum.

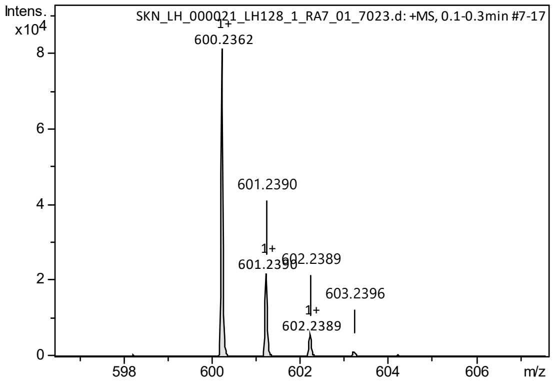

Figure S 31: High-resolution mass spectrum of compound **11f** with $[M+H]^{+}_{calc.}$= 600,23631 m/z.

11g

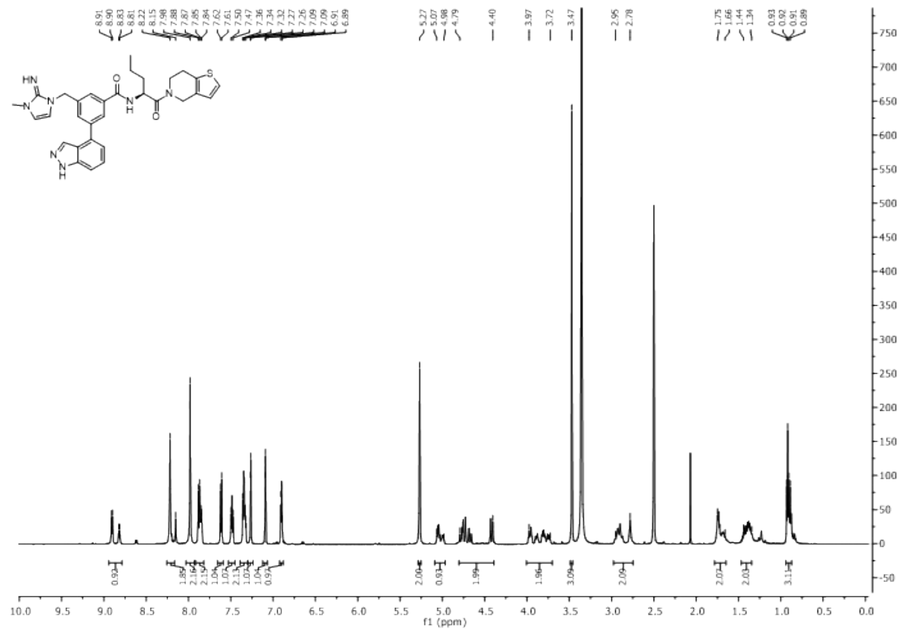

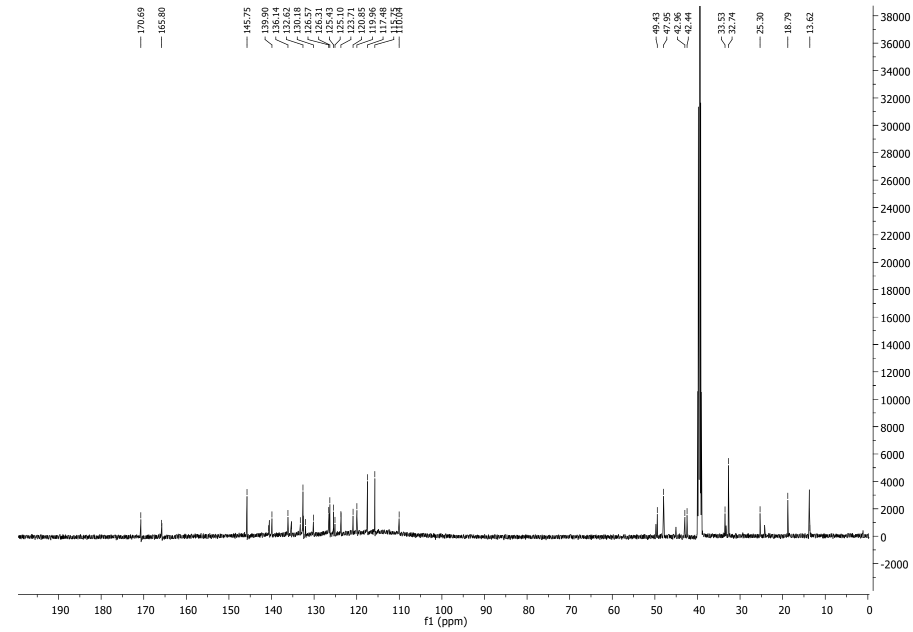

Figure S 32: ^{1}H- (top) and *C-NMR (bottom) of compound **11g**.

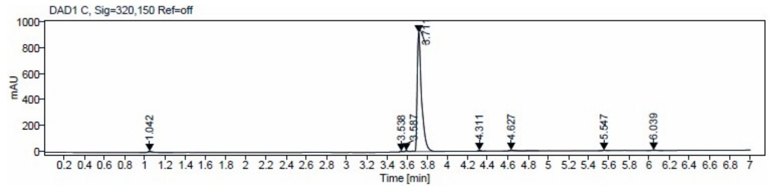

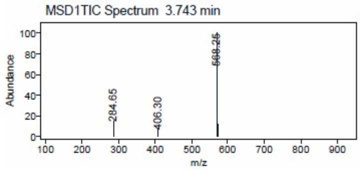

Figure S 33: LC/MS spectra of purified compound **11g** at 320 nm and ESI mass spectrum.

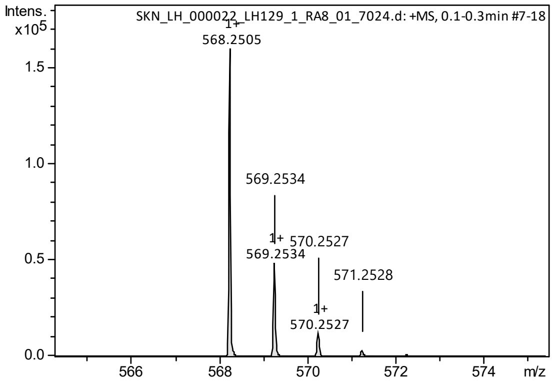

Figure S 34: High-resolution mass spectrum of compound **11g** with $[M+H]^{+}_{calc.}$= 568,24892 m/z.

11h

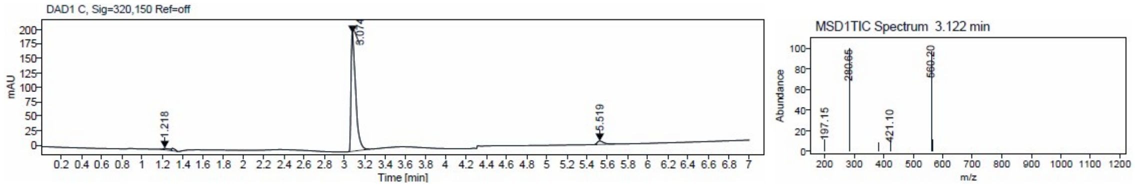

Figure S 35: LC/MS spectra of purified compound **11h** at 320 nm and ESI mass spectrum.

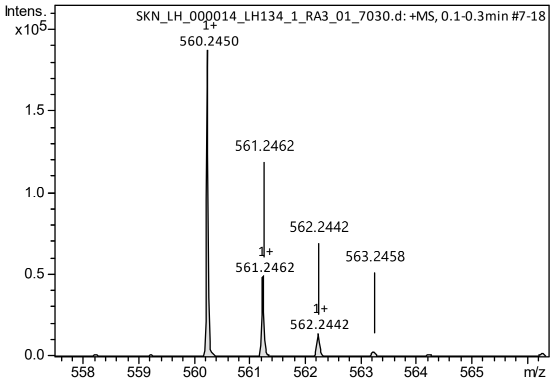

Figure S 36: High-resolution mass spectrum of compound **11h** with $[M+H]^{+}_{calc.}$= 560,24384 m/z.

11i

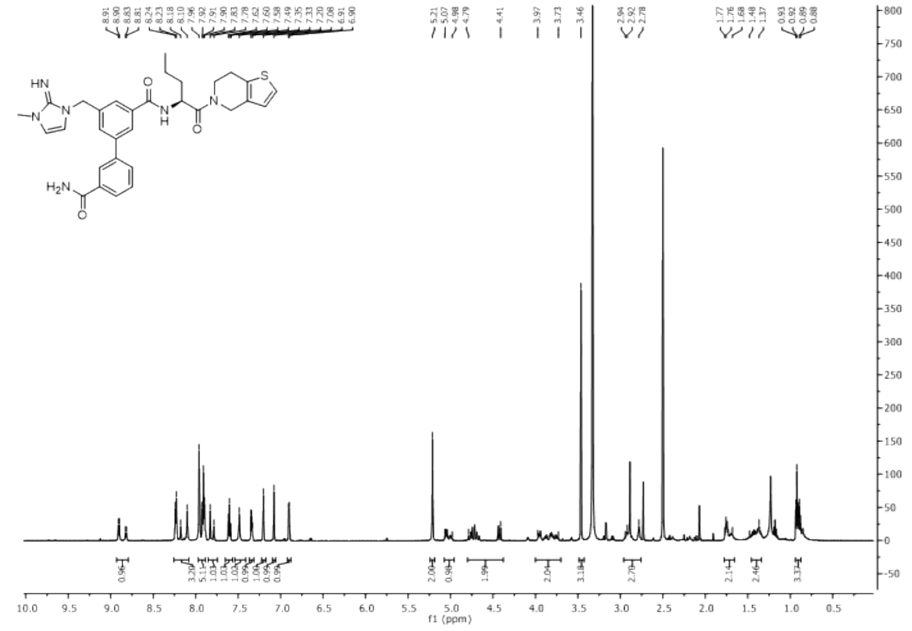

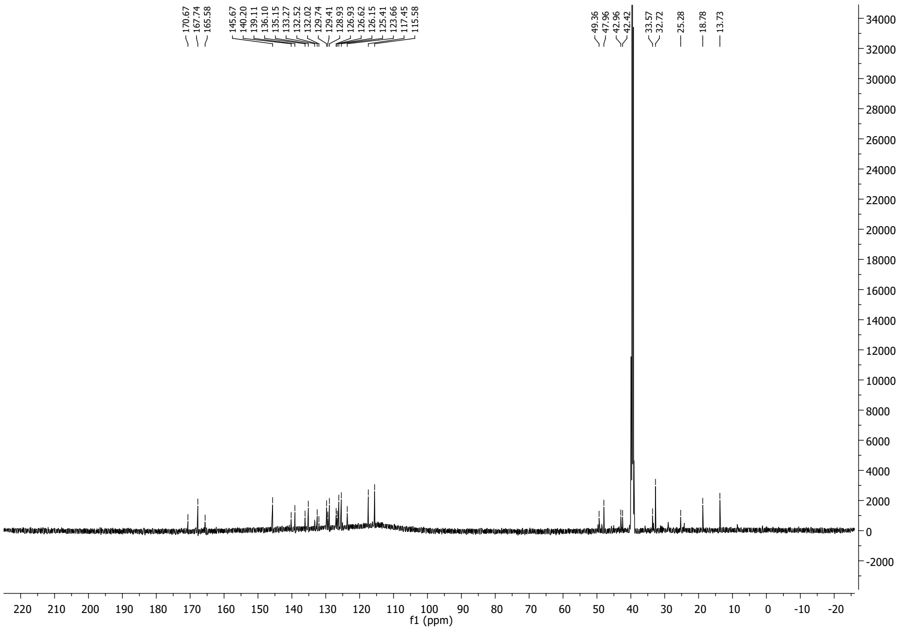

Figure S 37: ^{1}H- (top) and ^{13}C-NMR (bottom) of compound **11i**.

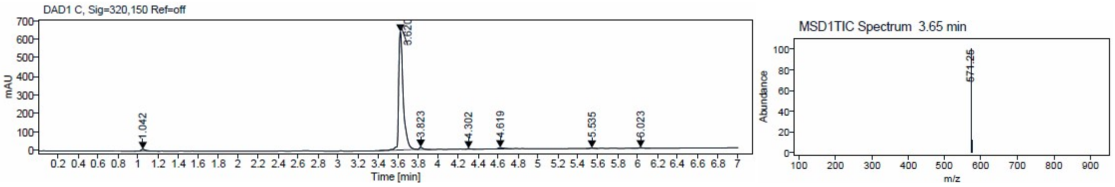

Figure S 38: LC/MS spectra of purified compound **11i** at 320 nm and ESI mass spectrum.

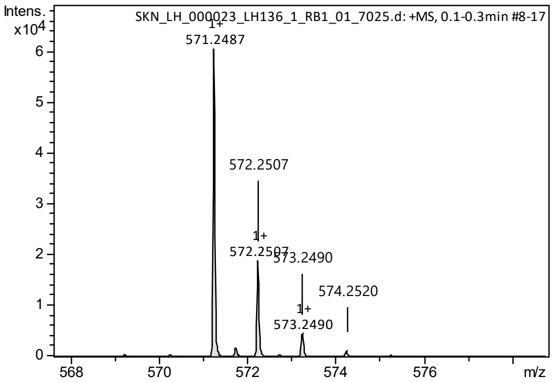

Figure S 39: High-resolution mass spectrum of compound **11i** with $[M+H]^{+}_{calc.}$= 571,24859 m/z

11j

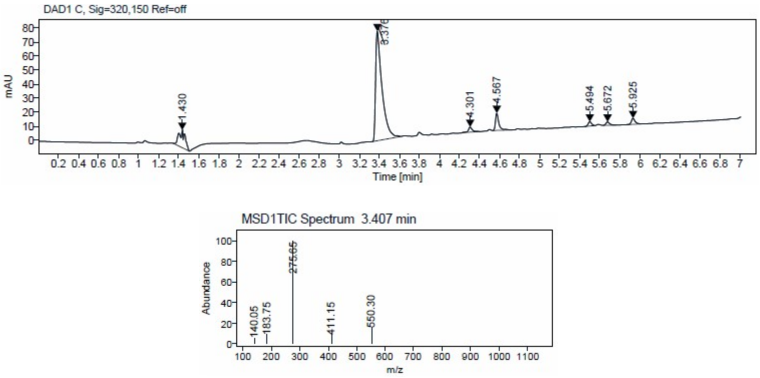

Figure S 40: LC/MS spectra of purified compound **11j** at 320 nm and ESI mass spectrum.

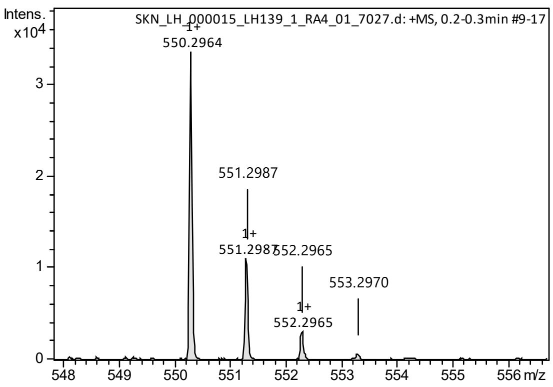

Figure S 41: High-resolution mass spectrum of compound **11j** with $[M+H]^{+}_{calc.}$= 550,29587 m/z

11k

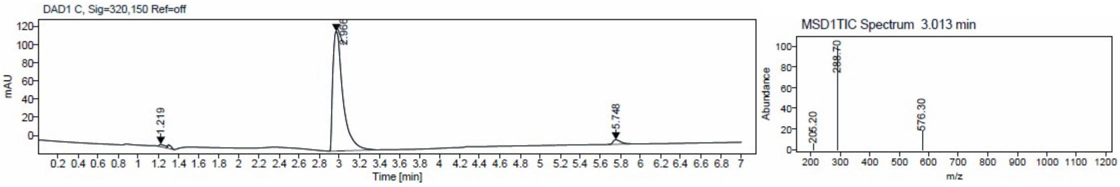

Figure S 42: LC/MS spectra of purified compound **11k** at 320 nm and ESI mass spectrum.

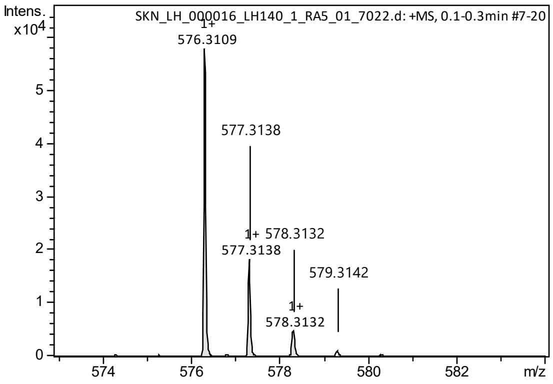

Figure S 43: High-resolution mass spectrum of compound **11k** with $[M+ H]^{+}_{calc.}$= 576,31152m/z

11l

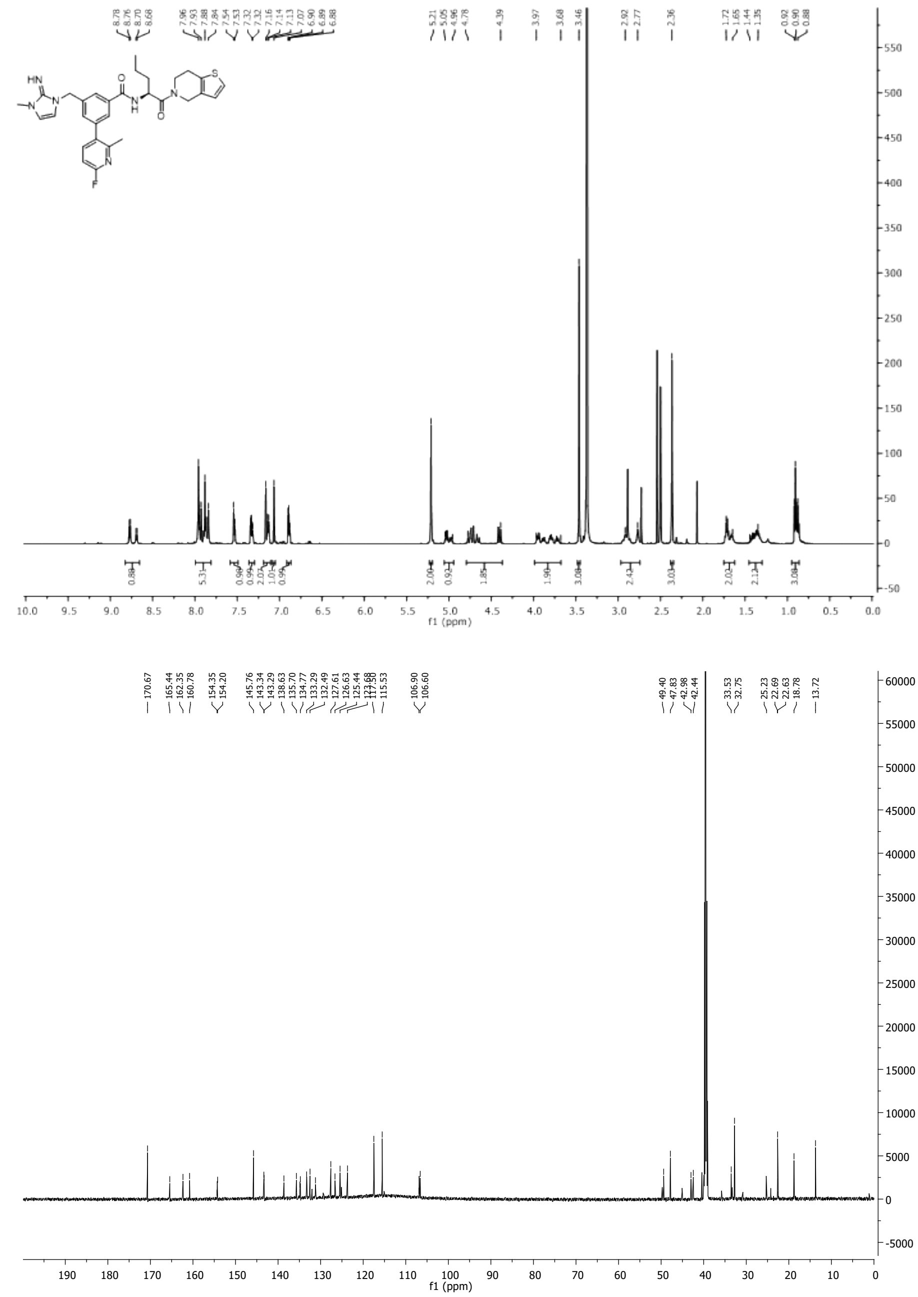

Figure S 44: ^{1}H- (top) and ^{13}C-NMR (bottom) of compound **11l**.

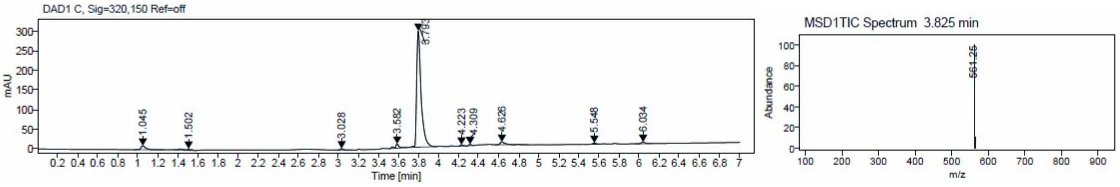

Figure S 45: LC/MS spectra of purified compound **11l** at 320 nm and ESI mass spectrum.

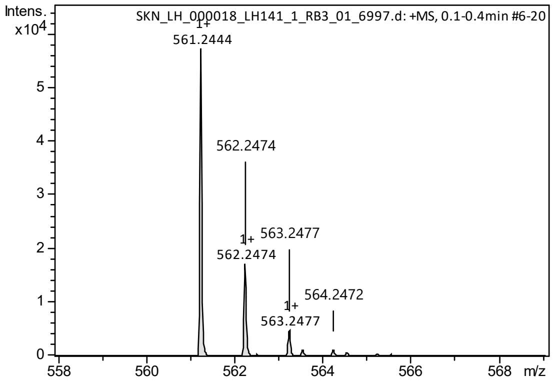

Figure S 46: High-resolution mass spectrum of compound **11l** with $[M+H]^{+}_{calc.}$= 561,24425 m/z.

11n

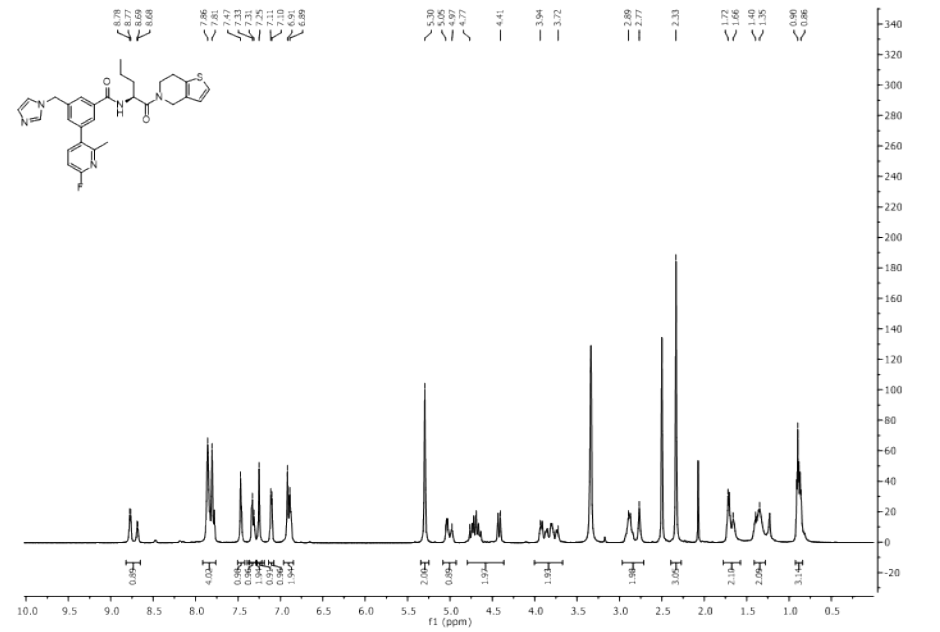

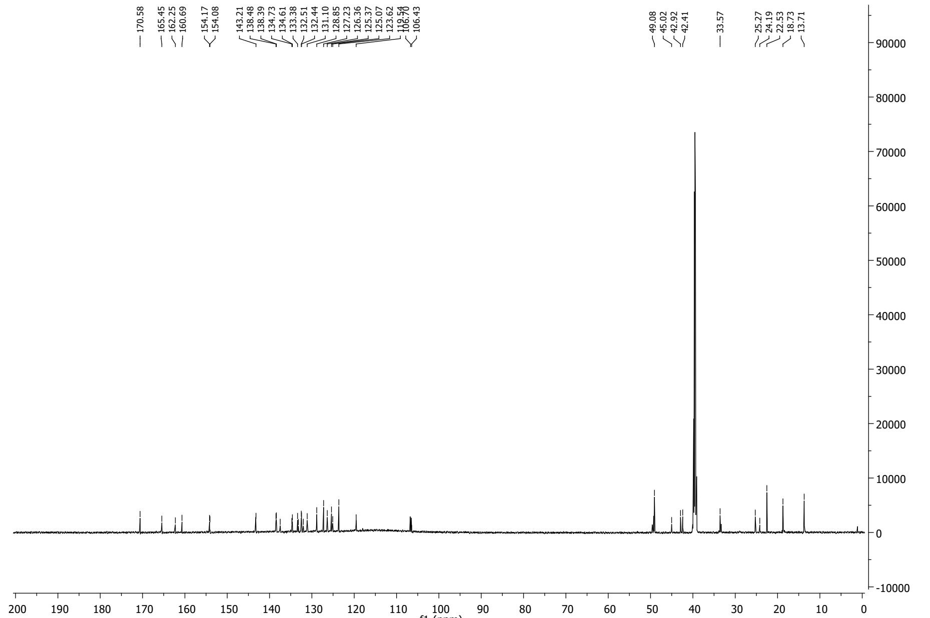

Figure S 47: ^{1}H- (top) and ^{13}C-NMR (bottom) of compound **11n**.

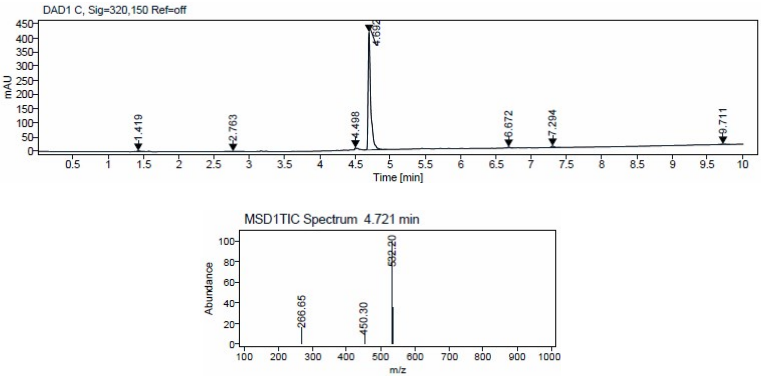

Figure S 48: LC/MS spectra of purified compound **11n** at 320 nm and ESI mass spectrum.

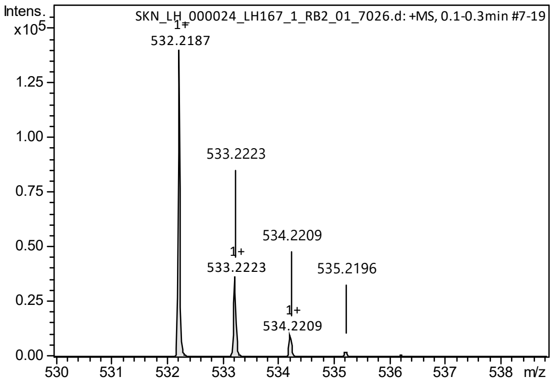

Figure S 49: High-resolution mass spectrum of compound **11n** with $[M+H]^+_{calc.}$= 532,21770 m/z.

11o (LH168)

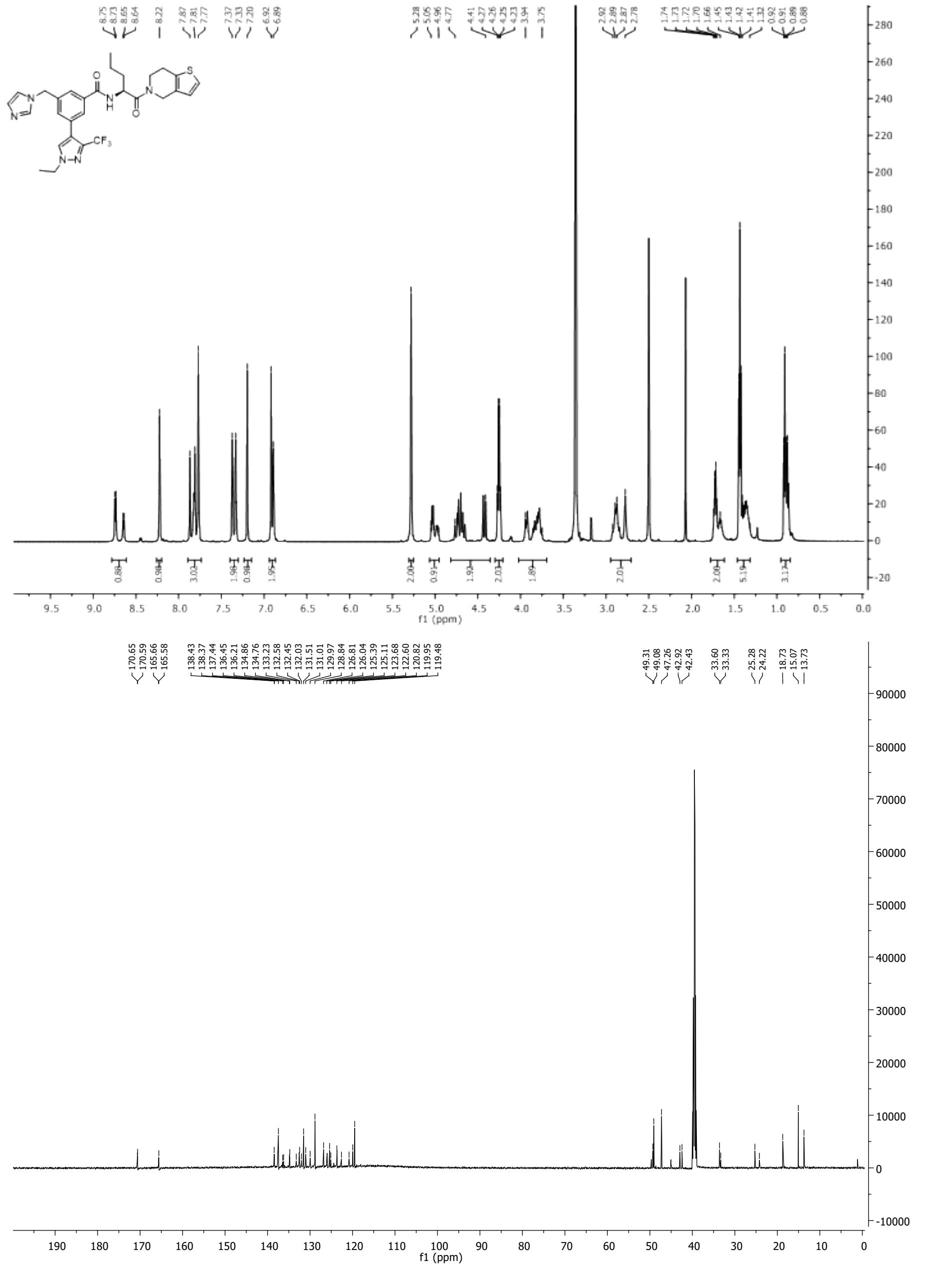

Figure S 50: ^{1}H- (top) and ^{13}C-NMR (bottom) of compound 11o.

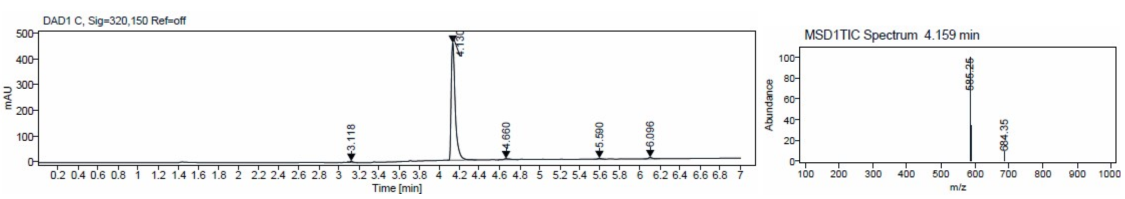

Figure S 51: LC/MS spectra of purified compound 11o at 320 nm and ESI mass spectrum.

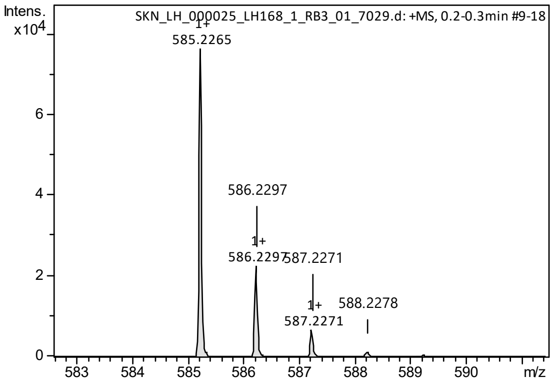

Figure S 52: High-resolution mass spectrum of compound 11o with $[M+H]^{+}_{calc.}$= 585,22541 m/z.

11p

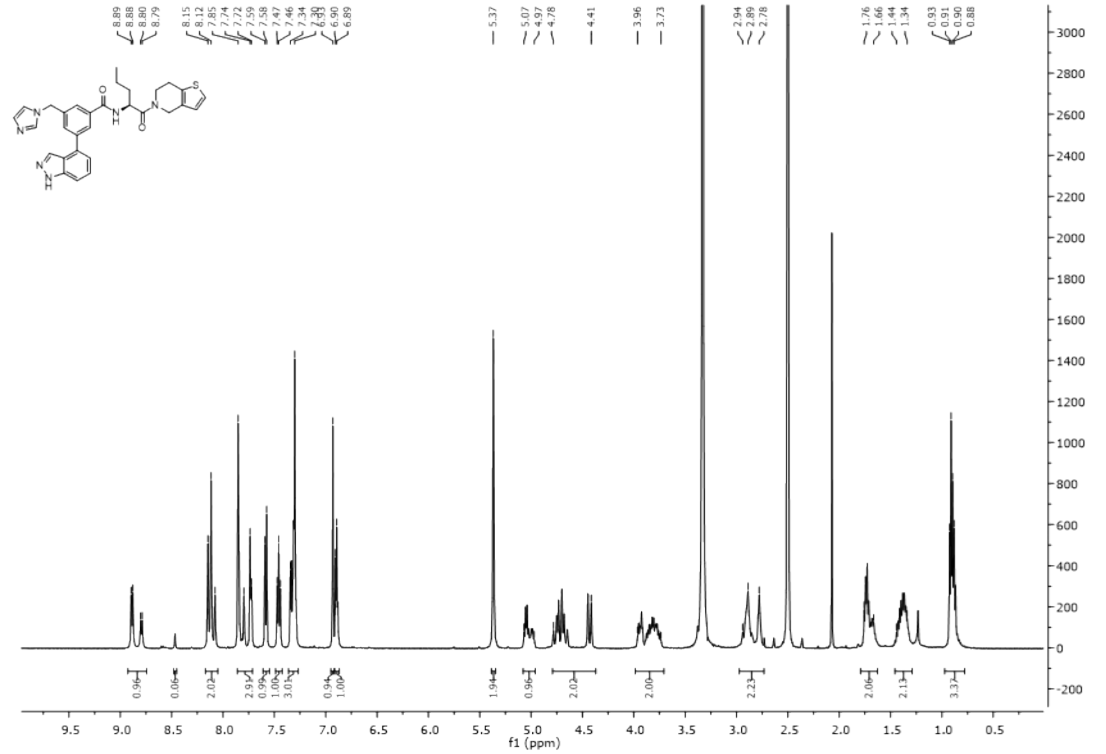

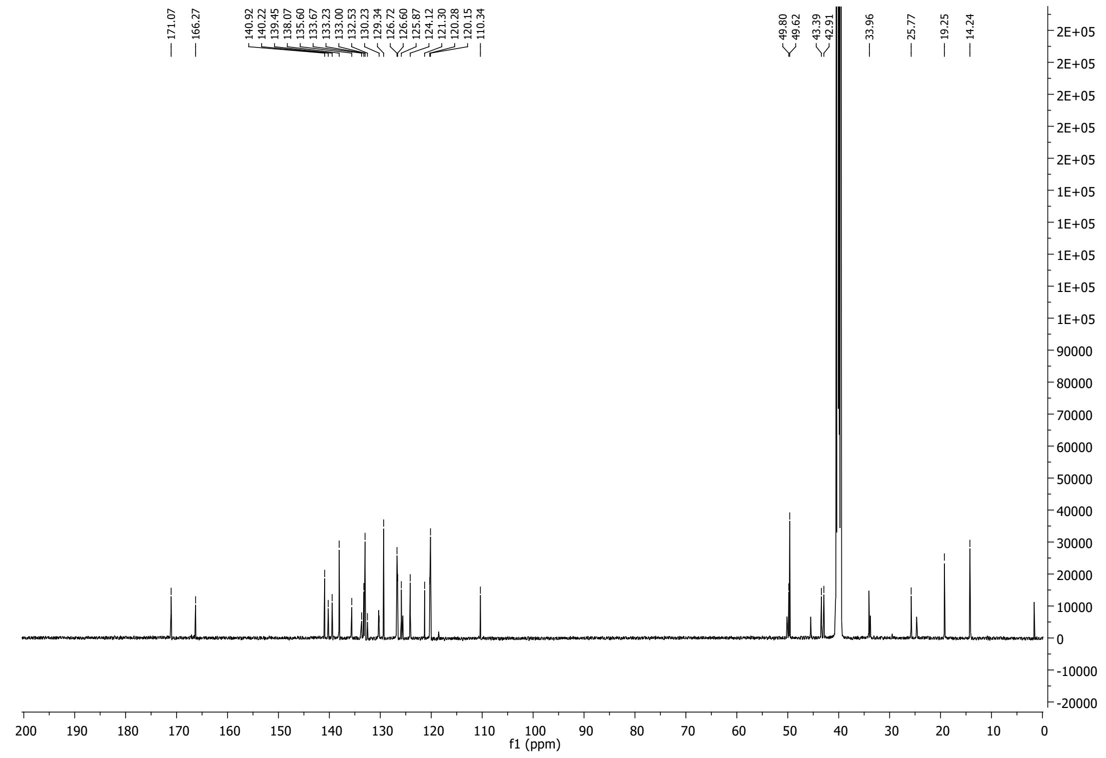

Figure S 53: ^{1}H- (top) and ^{13}C-NMR (bottom) of compound 11p.

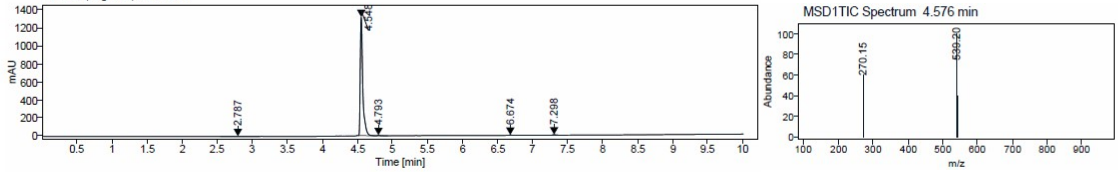

Figure S 54: LC/MS spectra of purified compound **11p** at 320 nm and ESI mass spectrum.

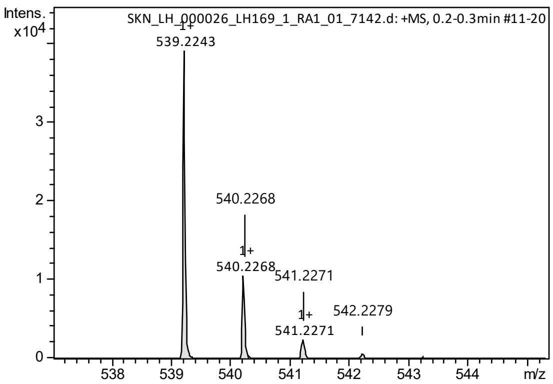

Figure S 55: High-resolution mass spectrum of compound **11p** with $[M+H]^+_{calc.}$= 539,22237 m/z.

11m

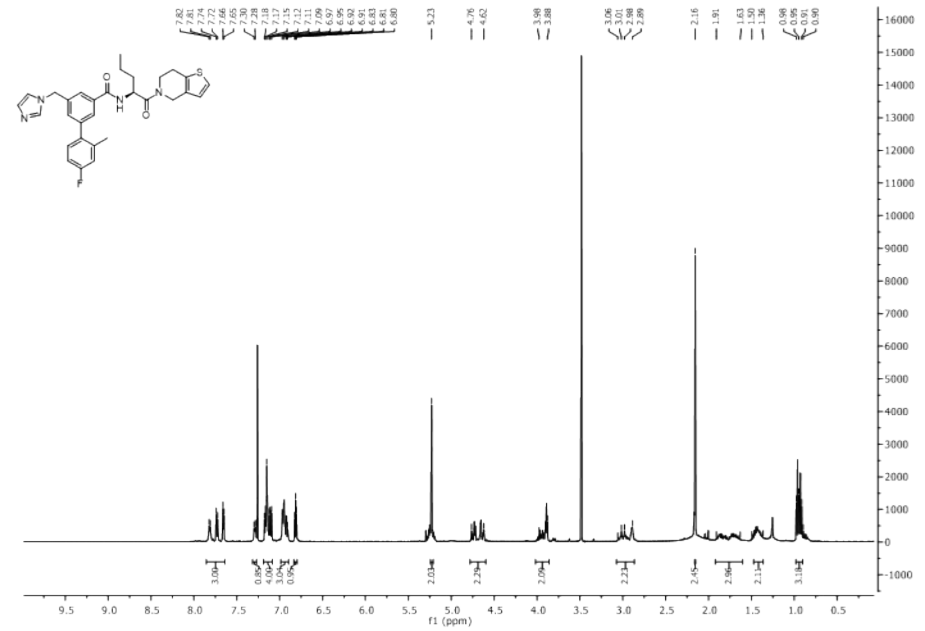

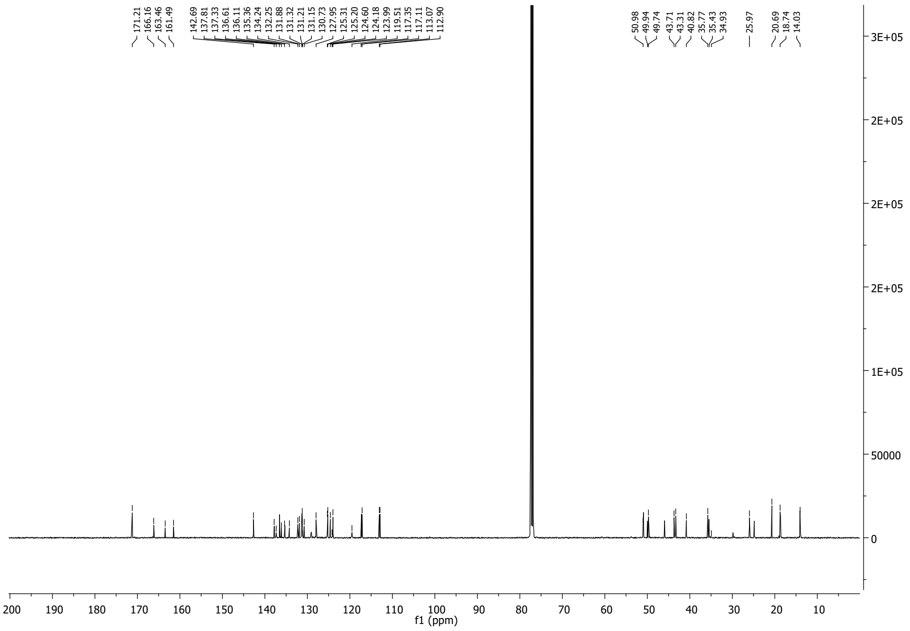

Figure S 56: ^{1}H- (top) and ^{13}C-NMR (bottom) of compound **11m**.

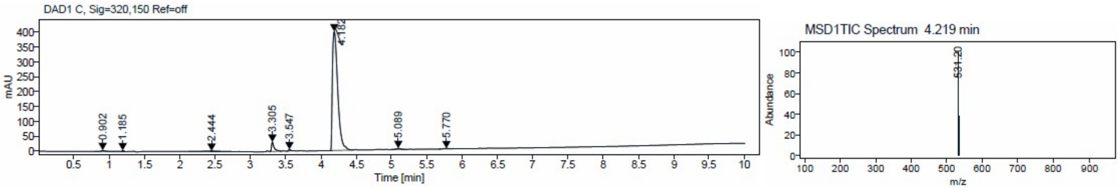

Figure S 57: LC/MS spectra of purified compound **11m** at 320 nm and ESI mass spectrum.

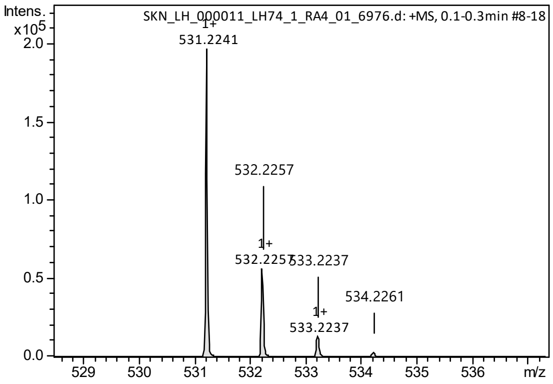

Figure S 58: High-resolution mass spectrum of compound **11m** with $[M+H]^{+}_{calc.}$= 531,22245 m/z.

13

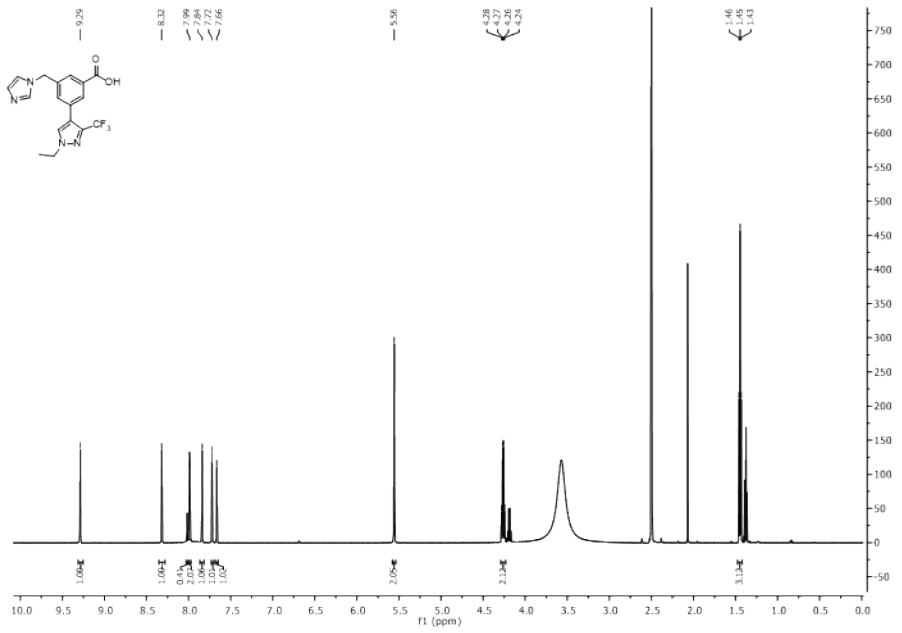

Figure S 59: ^{1}H-NMR of compound **13**.

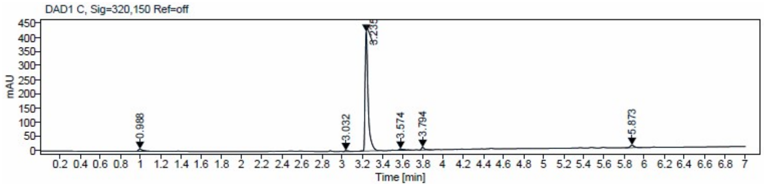

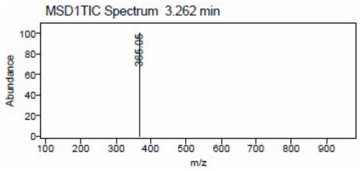

Figure S 60: LC/MS spectra of purified compound **13** at 320 nm and ESI mass spectrum.

15

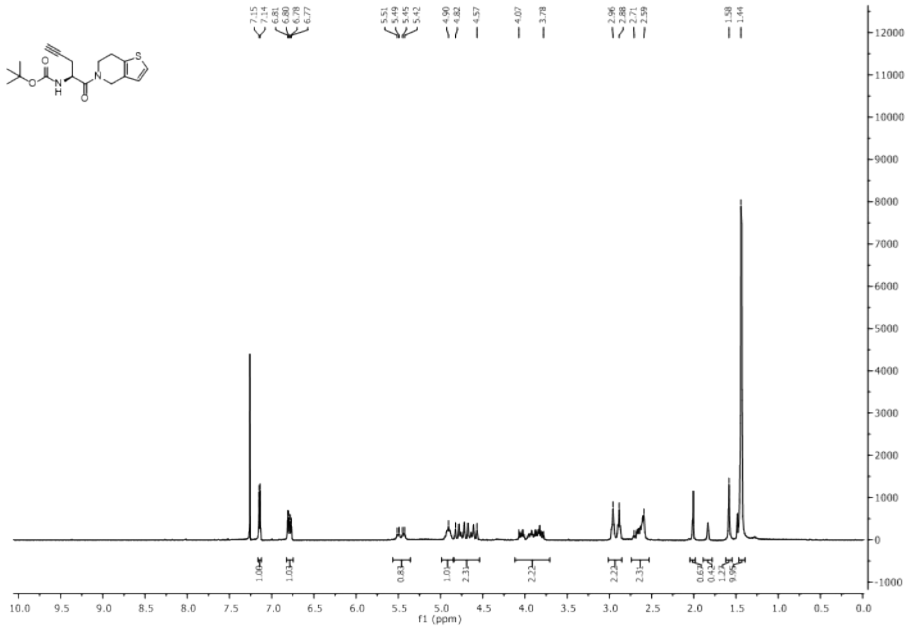

Figure S 61: ^{1}H-NMR of compound **15**.

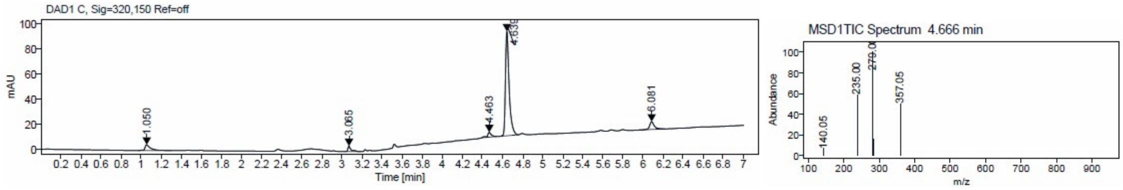

Figure S 62: LC/MS spectra of purified compound **15** at 320 nm and ESI mass spectrum.

16

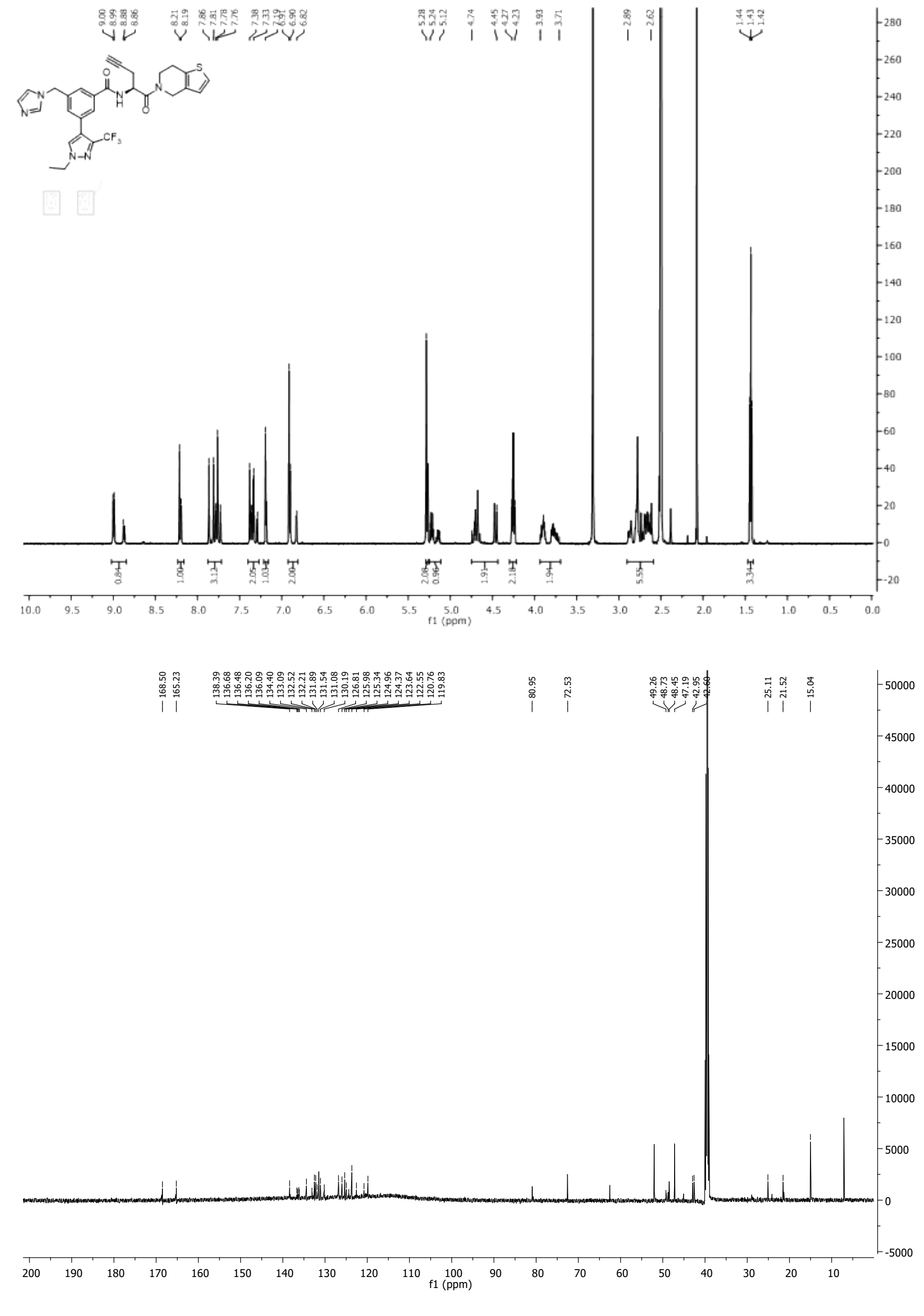

Figure S 63: ^{1}H- (top) and ^{13}C-NMR (bottom) of compound **16**.

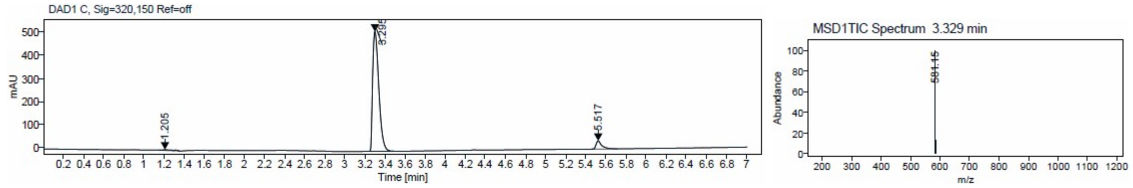

Figure S 64: LC/MS spectra of purified compound **16** at 320 nm and ESI mass spectrum.

LH222

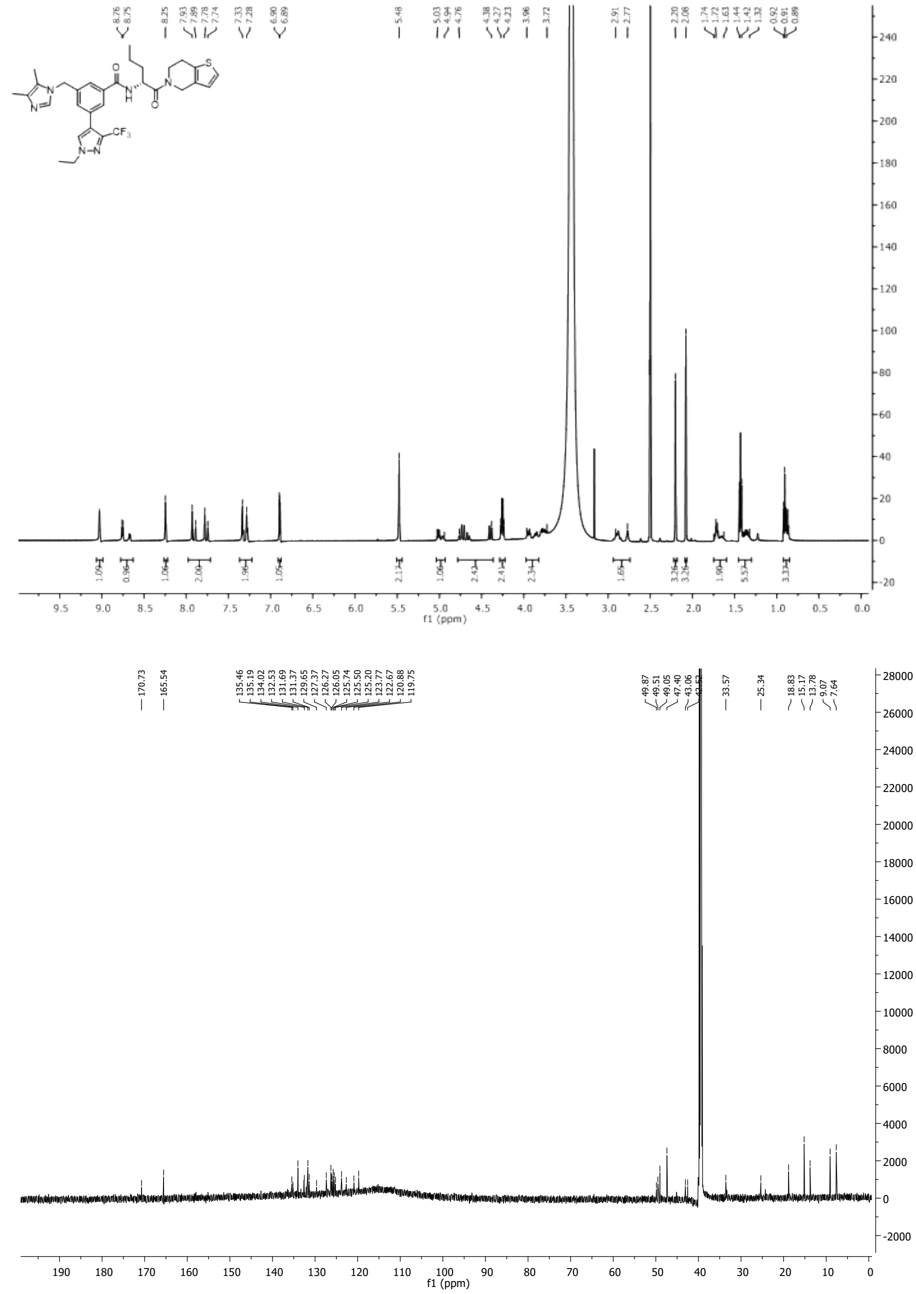

Figure S 65: ^{1}H- (top) and ^{13}C-NMR (bottom) of compound **LH222**.

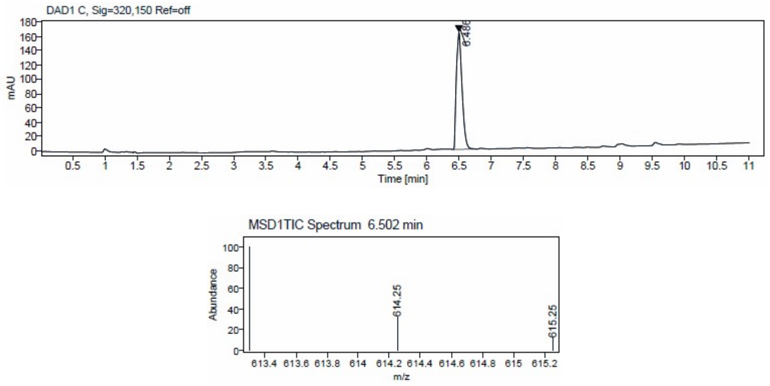

Figure S 66: LC/MS spectra of purified compound **LH222** at 320 nm and ESI mass spectrum.

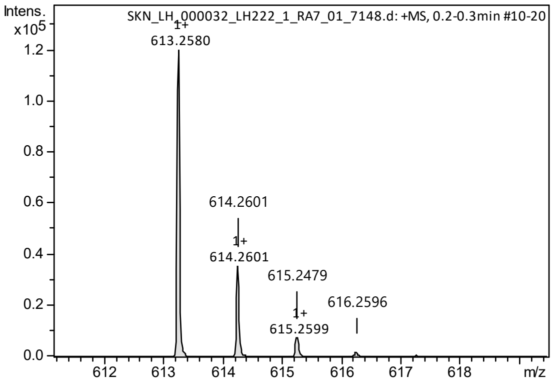

Figure S 67: High-resolution mass spectrum of compound **LH222** with $[M+H]^{+}_{calc.}$= 613,25671 m/z.

7

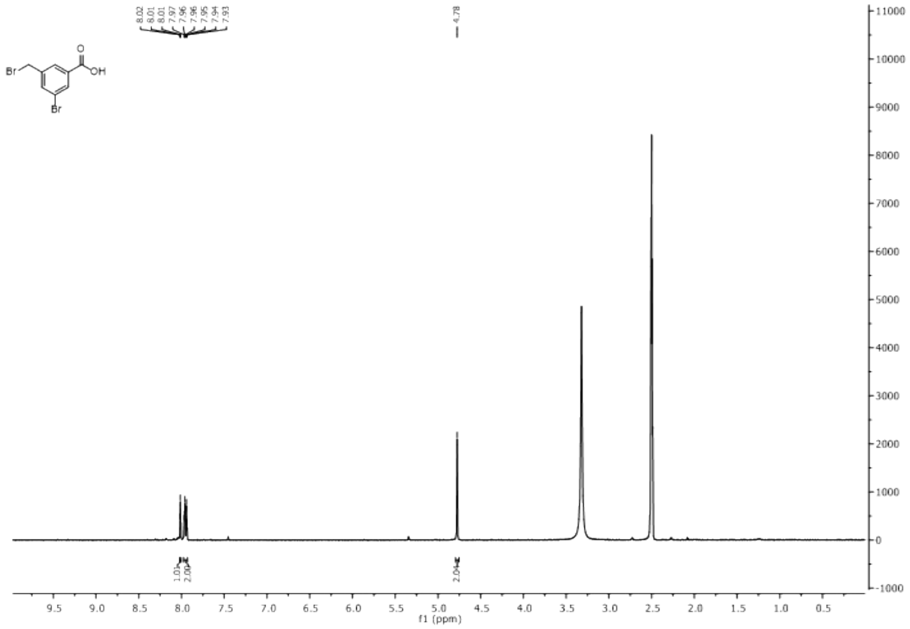

Figure S 68: ^{1}H-NMR of compound **7**.

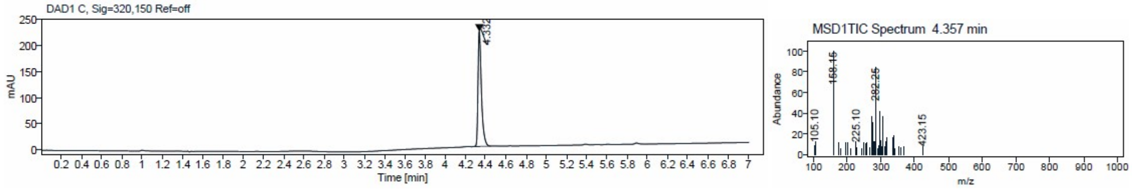

Figure S 69: LC/MS spectra of purified compound **7** at 320 nm and *ESI mass spectrum.*

17a

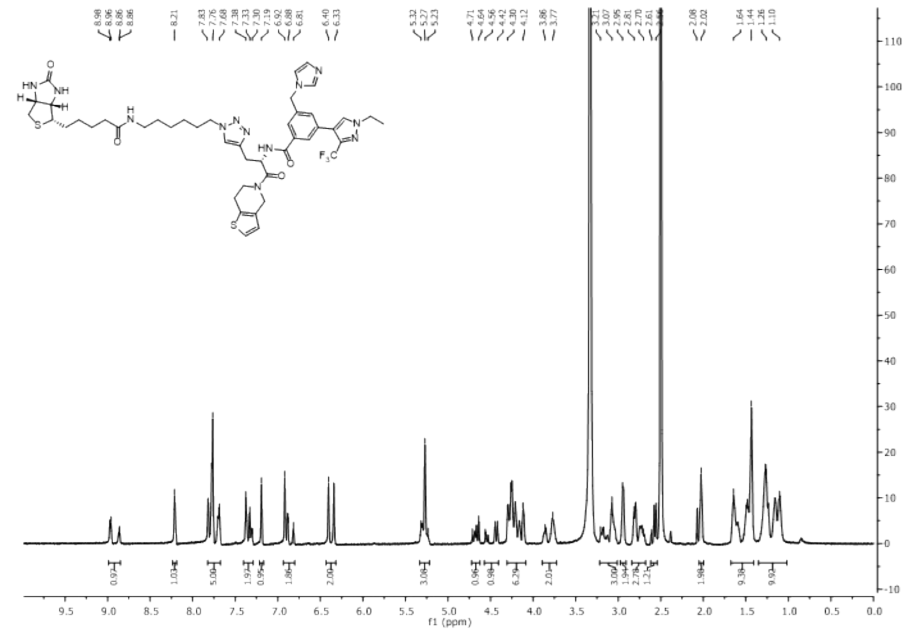

Figure S 70: ^{1}H-NMR of compound **17a**.

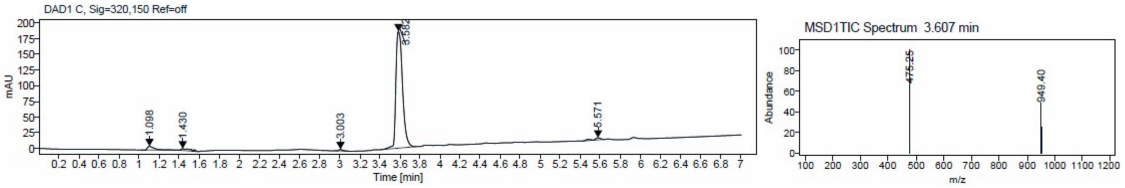

Figure S 71: LC/MS spectra of purified compound **17a** at 320 nm and ESI mass spectrum.

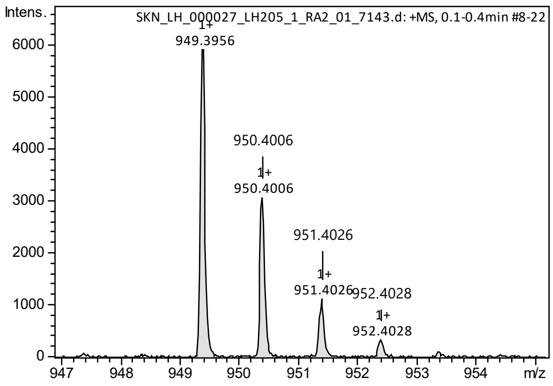

Figure S 72: High-resolution mass spectrum of compound **17a** with $[M+H]^+_{calc.}$= 949,39355 m/z.

17b

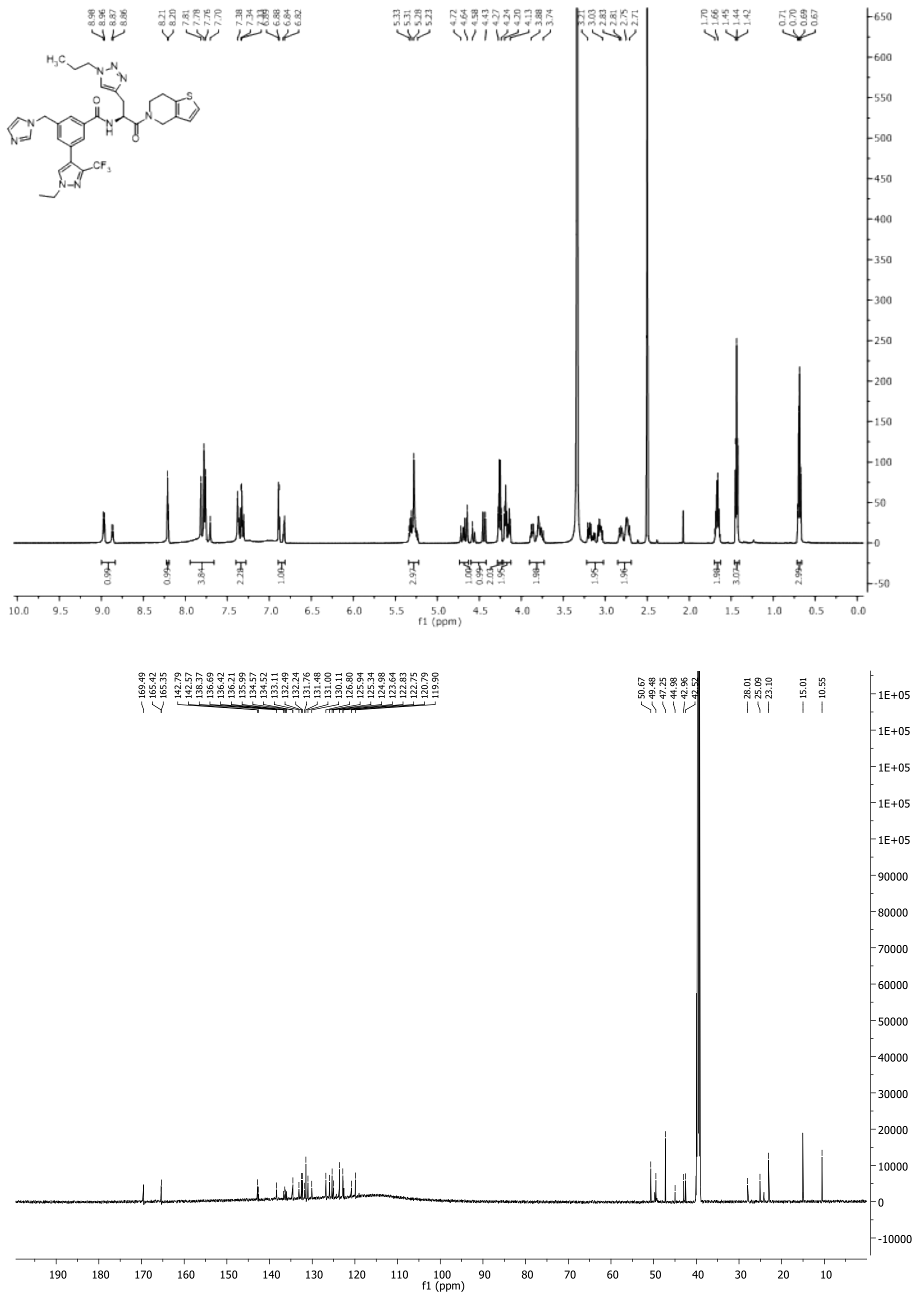

Figure S 73: ^{1}H- (top) and ^{13}C-NMR (bottom) of compound **17b**.

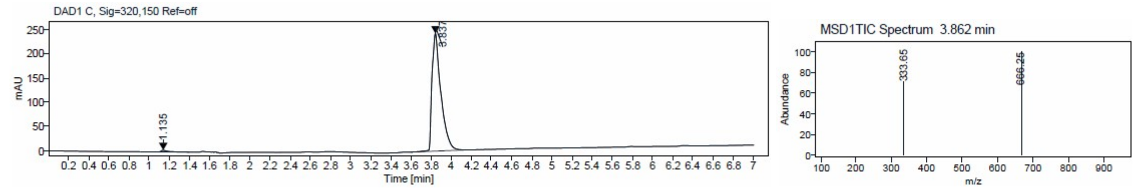

Figure S 74: LC/MS spectra of purified compound **17b** at 320 nm and ESI mass spectrum.

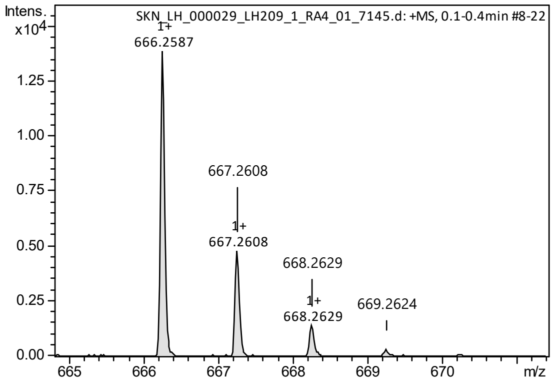

Figure S 75: High-resolution mass spectrum of compound **17b** with $[M+H]^{+}_{calc.}$= 666,25810 m/z.

18

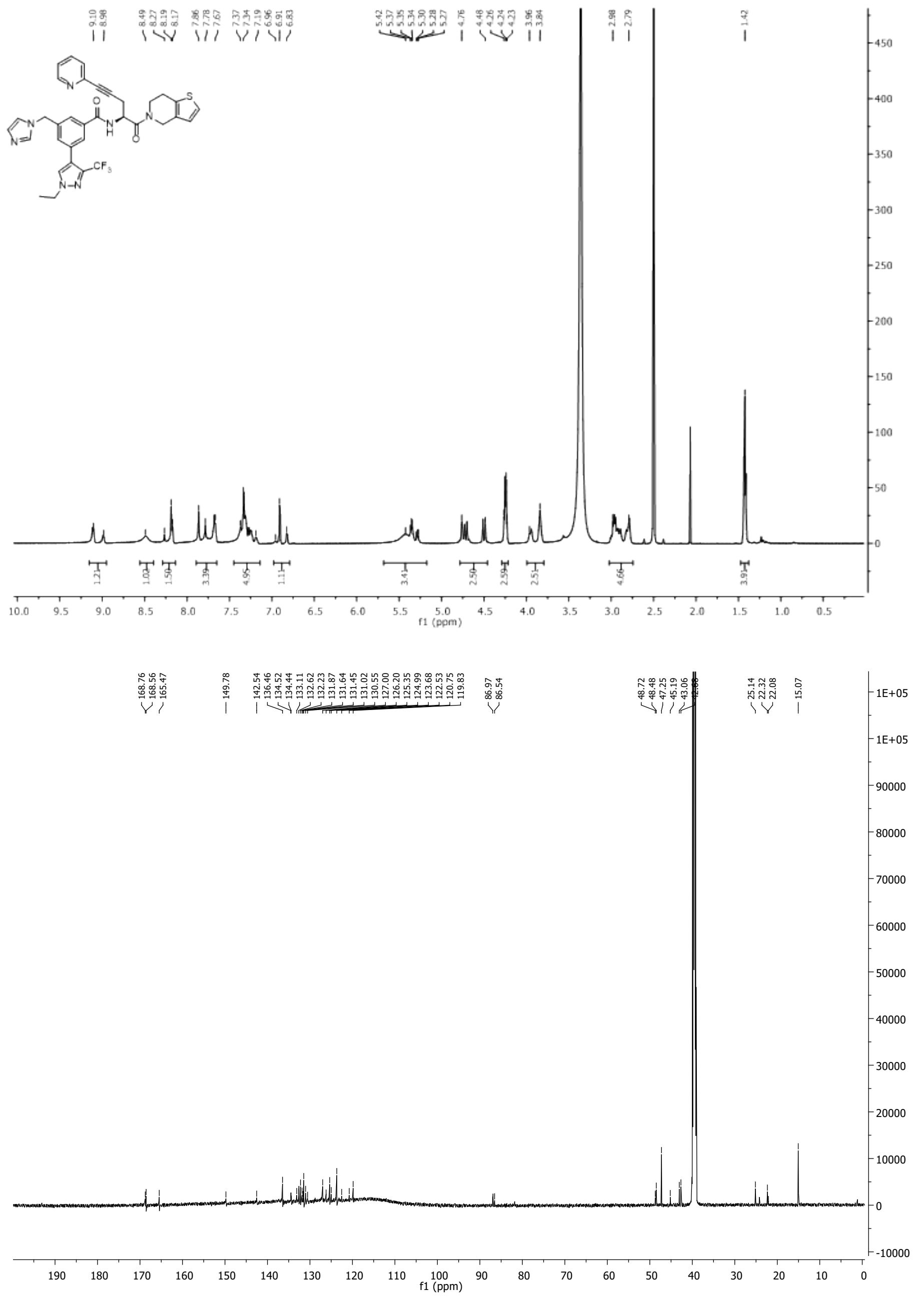

Figure S 76: ^{1}H- (top) and ^{13}C-NMR (bottom) of compound **18**.

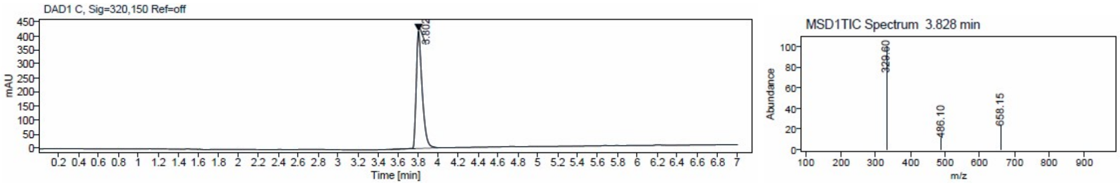

Figure S 77: LC/MS spectra of purified compound **18** at 320 nm and ESI mass spectrum.

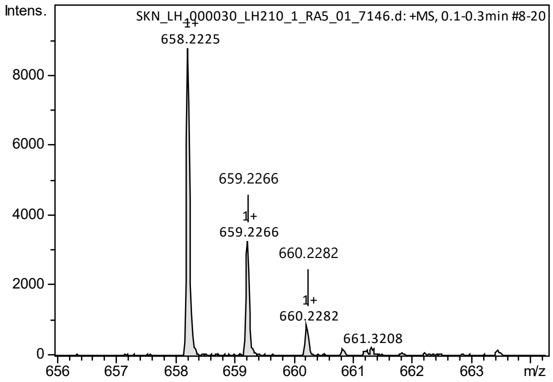

Figure S 78: High-resolution mass spectrum of compound **18** with $[M+H]^+_{calc.}$= 658,22066 m/z.

8a

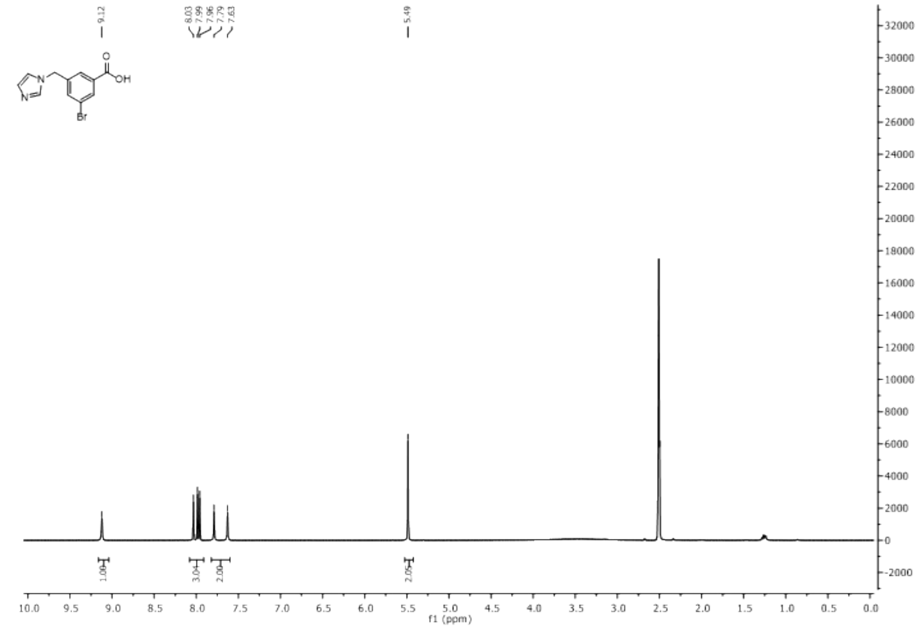

Figure S 79: ^{1}H-NMR of compound **8a**.

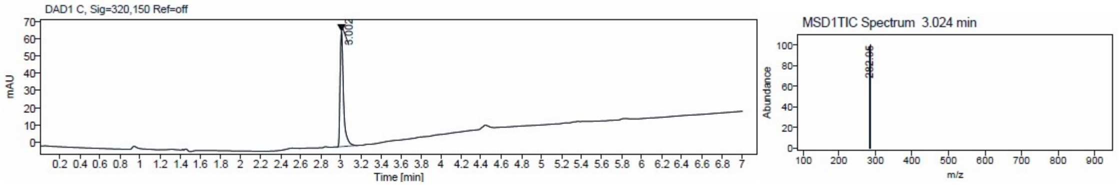

Figure S 80: LC/MS spectra of purified compound **8a** at 320 nm and ESI mass spectrum.

10a

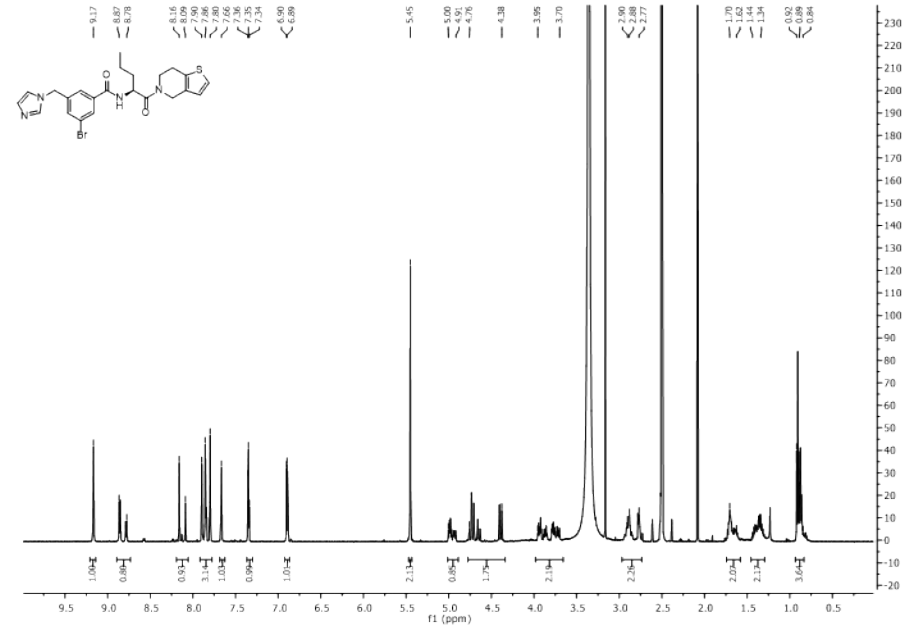

Figure S 81: ^{1}H-NMR of compound **10a**.

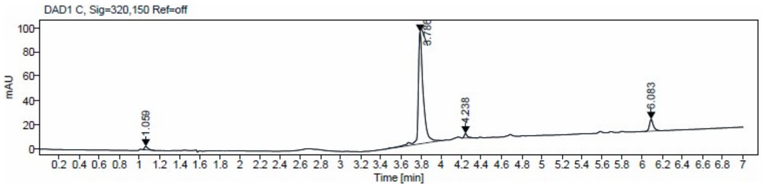

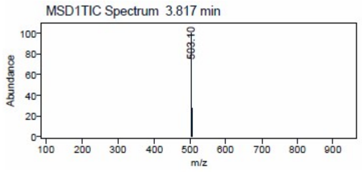

Figure S 82: LC/MS spectra of purified compound **10a** at 320 nm and ESI mass spectrum.

8b

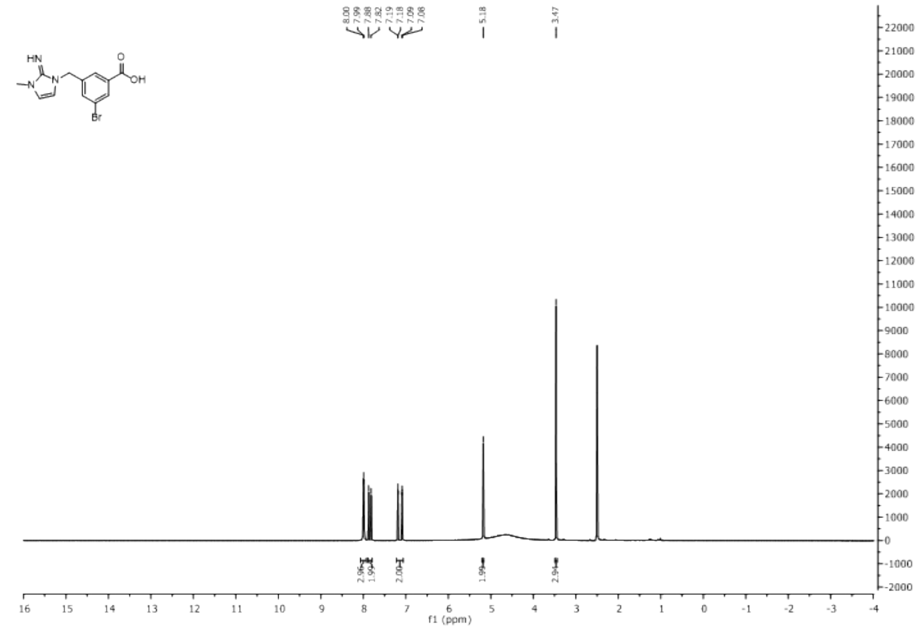

Figure S 83: ^{1}H-NMR of compound **8b**.

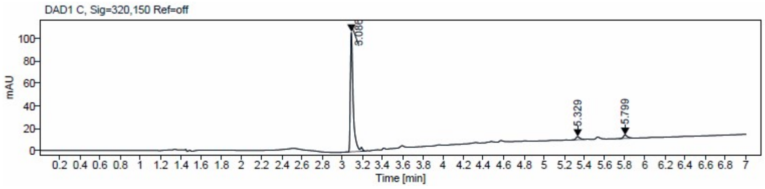

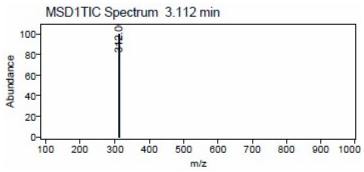

Figure S 84: LC/MS spectra of purified compound **8b** at 320 nm and ESI mass spectrum.

10b

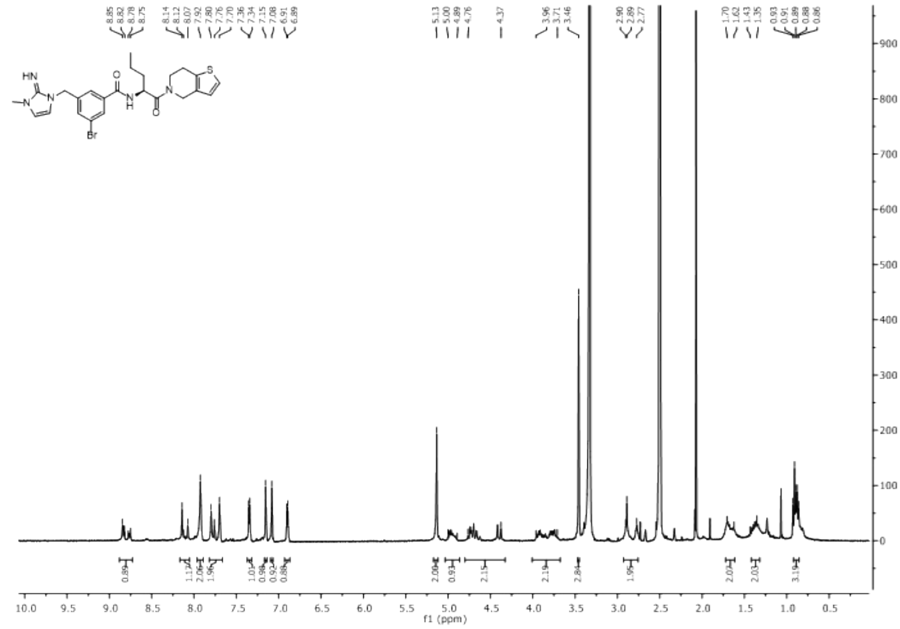

Figure S 85: ^{1}H-NMR of compound **10b**.

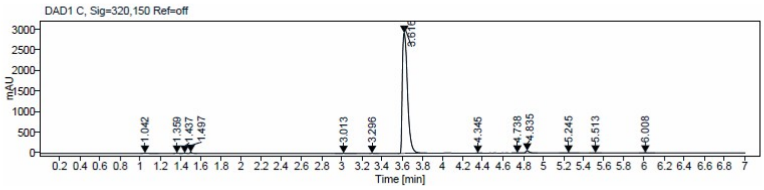

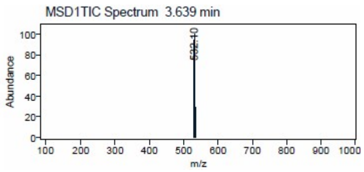

Figure S 86: LC/MS spectra of purified compound **10b** at 320 nm and ESI mass spectrum.

LH214

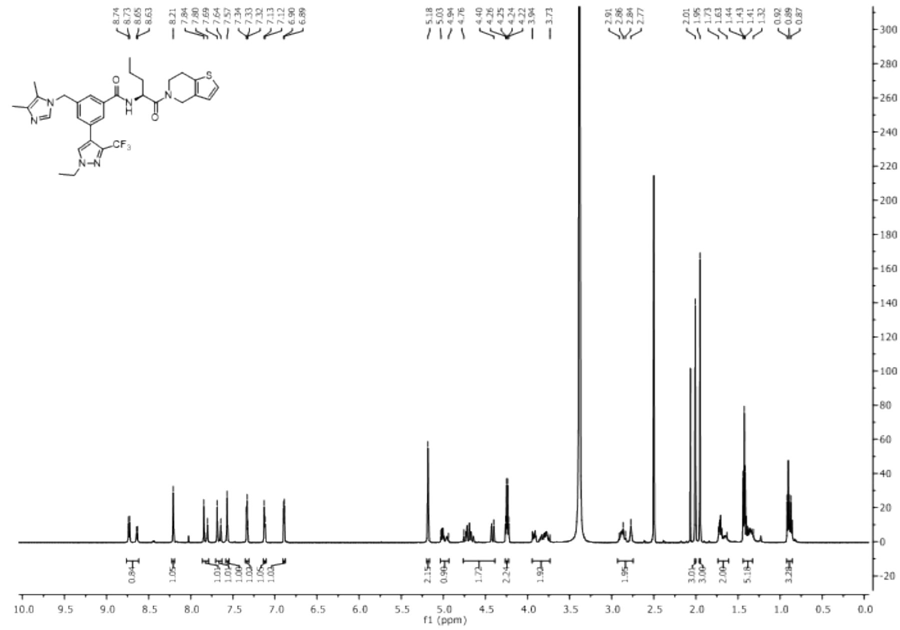

Figure S 87: ^{1}H-NMR of compound (LH214).

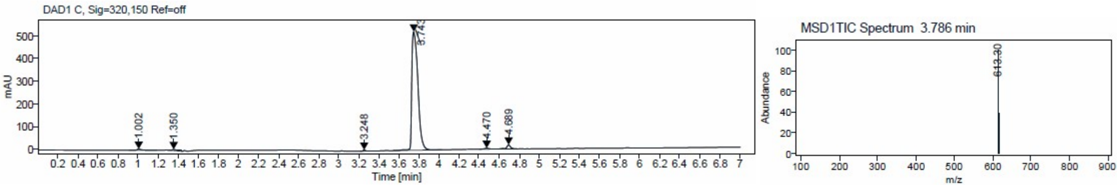

Figure S 88: LC/MS spectra of purified compound (LH214) at 320 nm and ESI mass spectrum.

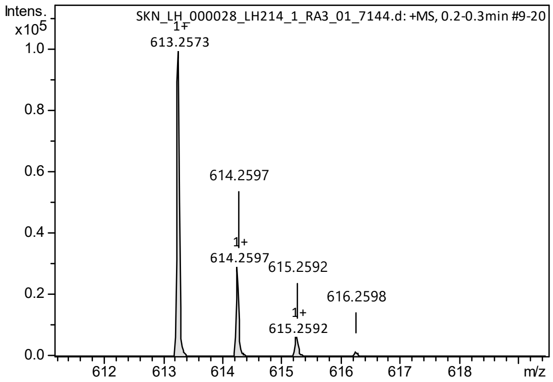

Figure S 89: High-resolution mass spectrum of compound (LH214) with $[M+H]^+_{calc.}$= 539,22237 m/z.

LH224

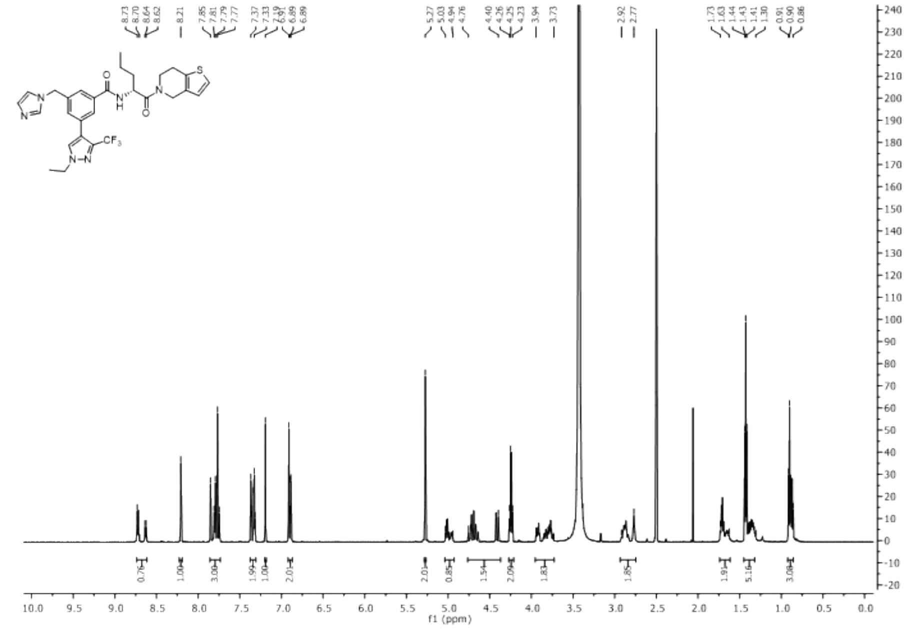

Figure S 90: ^{1}H-NMR of compound (LH224).

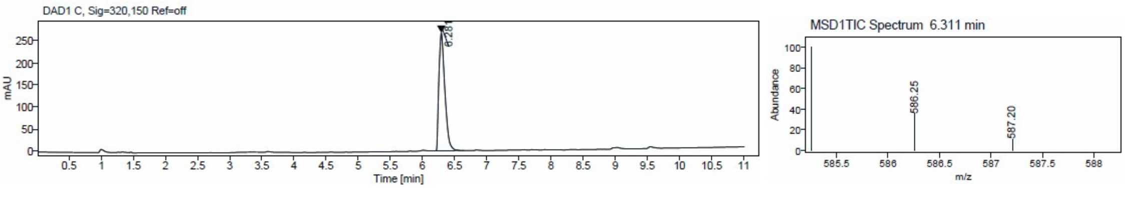

Figure S 91: LC/MS spectra of purified compound (LH224) at 320 nm and ESI mass spectrum.

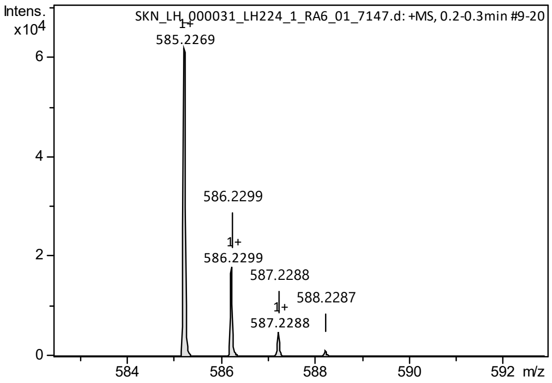

Figure S 92: High-resolution mass spectrum of compound (LH224) with $[M+H]^{+}_{calc.}$= 539,22237 m/z.

4. References

1 A. Allali-Hassani, M. M. Szewczyk, D. Ivanochko, S. L. Organ, J. Bok, J. S. Y. Ho, F. P. H. Gay, F. Li, L. Blazer, M. S. Eram, L. Halabelian, D. Dilworth, G. M. Luciani, E. Lima-Fernandes, Q. Wu, P. Loppnau, N. Palmer, S. Z. A. Talib, P. J. Brown, M. Schapira, P. Kaldis, R. C. O'Hagan, E. Guccione, D. Barsyte-Lovejoy, C. H. Arrowsmith, J. M. Sanders, S. D. Kattar, D. J. Bennett, B. Nicholson and M. Vedadi, *Nat. Commun.*, 2019, **10**, 5759.

2 D. Bhattacharya, A. Shi Ming Li, B. Paul, U. Ghosh Dastidar, V. Santhakumar, D. Sarkar, I. Chau, F. Li, T. Ghosh, M. Vedadi and A. Talukdar, *Eur. J. Med. Chem.*, 2023, **260**, 115713.

3 M. Rafiee, G. Sigismondo, M. Kalxdorf, L. Förster, B. Brügger, J. Béthune and J. Krijgsveld, *Mol. Syst. Biol.*, 2020, **16**, e9370.

4 M. P. Schwalm, K. Saxena, S. Müller and S. Knapp, *Nat. Protoc.*, 2024, **19**, 2317–2357.

5 J. Dopfer, J. D. Vasta, S. Müller, S. Knapp, M. B. Robers and M. P. Schwalm, *Nat. Commun.*, 2024, **15**, 5646.

6 W. Minor, M. Cymborowski, Z. Otwinowski and M. Chruszcz, *Acta Crystallogr. D Biol. Crystallogr.*, 2006, **62**, 859–866.

7 A. J. McCoy, R. W. Grosse-Kunstleve, P. D. Adams, M. D. Winn, L. C. Storoni and R. J. Read, *J. Appl. Crystallogr.*, 2007, **40**, 658–674.

8 P. Emsley and K. Cowtan, *Acta Crystallogr. D Biol. Crystallogr.*, 2004, **60**, 2126–2132.

9 G. N. Murshudov, A. A. Vagin and E. J. Dodson, *Acta Crystallogr. D Biol. Crystallogr.*, 1997, **53**, 240–255.

10 M. D. Winn, C. C. Ballard, K. D. Cowtan, E. J. Dodson, P. Emsley, P. R. Evans, R. M. Keegan, E. B. Krissinel, A. G. W. Leslie, A. McCoy, S. J. McNicholas, G. N. Murshudov, N. S. Pannu, E. A. Potterton, H. R. Powell, R. J. Read, A. Vagin and K. S. Wilson, *Acta Crystallogr. D Biol. Crystallogr.*, 2011, **67**, 235–242.

11 V. B. Chen, W. B. Arendall, J. J. Headd, D. A. Keedy, R. M. Immormino, G. J. Kapral, L. W. Murray, J. S. Richardson and D. C. Richardson, *Acta Crystallogr. D Biol. Crystallogr.*, 2010, **66**, 12–21.
